# Supplementary material for: Integrating spatial and single-cell transcriptomics data using deep generative models with SpatialScope
Source: Nat Commun. 2023 Nov 29;14:7848. doi: 10.1038/s41467-023-43629-w (PMC10687049; doi:10.1038/s41467-023-43629-w)
Supplement: Supplementary file 1 — Supplementary Information [file 41467_2023_43629_MOESM1_ESM.pdf]

Supplementary of “Integrating spatial and single-cell transcriptomics data using deep generative models with SpatialScope”

Contents

1 Supplementary Figures **2**

2 Supplementary Methods **83**

2.1 Nucleus segmentation. 83
2.2 Cell type identification. 84
2.2.1 Model setting 84
2.2.2 Spot-specific effect α_i accounting for platform differences 85
2.2.3 Gene-specific effect γ_g accounting for platform difference 85
2.2.4 Cell type identification 86
2.3 Score-based generative models 88
2.4 SpatialScope: a conditional score-based generative model for single-cell reference data 90
2.5 Network Architectures 90
2.6 Hyper-parameters 91
2.7 Correction of the batch effects between single-cell reference and ST data . . . 91
2.8 The comparison between SpatialScope and RCTD 92
2.9 Simulation design 95
2.9.1 Benchmarking datasets 95
2.9.2 Different utilities between SpatialScope (Cell type identification) and RCTD. 97
2.9.3 The baseline method “StarDist+RCTD” 97
2.9.4 Leveraging spatial information 97
2.9.5 Missing cell types in single-cell reference 98
2.9.6 Inconsistent cell number in gene expression decomposition task 99
2.9.7 Hyperparameters sensitivity analysis for training the score-based generative model 100
2.9.8 The comparison of conditional and unconditional score function 102
2.9.9 Unbalanced cell types in single-cell reference data 102
2.9.10 Unbalanced cell numbers within the spots 103
2.9.11 Performance under different grid sizes 104
2.9.12 The impact of abundance and variability for gene expression imputation 104

2.9.13 The generalization ability of gene expression imputation for gene panels from diverse tissues 106

1 Supplementary Figures

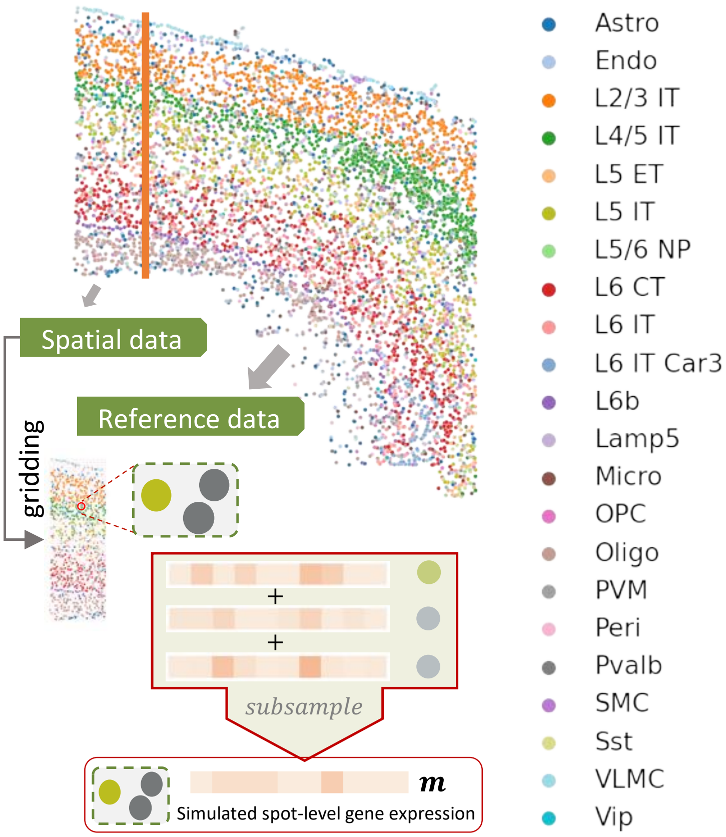

Figure S1: The process of generating a simulated dataset using MERFISH dataset. MERFISH cells are divided into two subsets. The left part is used to make pseudo-spots by aggregating the cells within each grid, and the right part is regarded as a paired scRNA-seq reference. Uniform gridding is performed and cells in squares are aggregated to generate simulated spots.

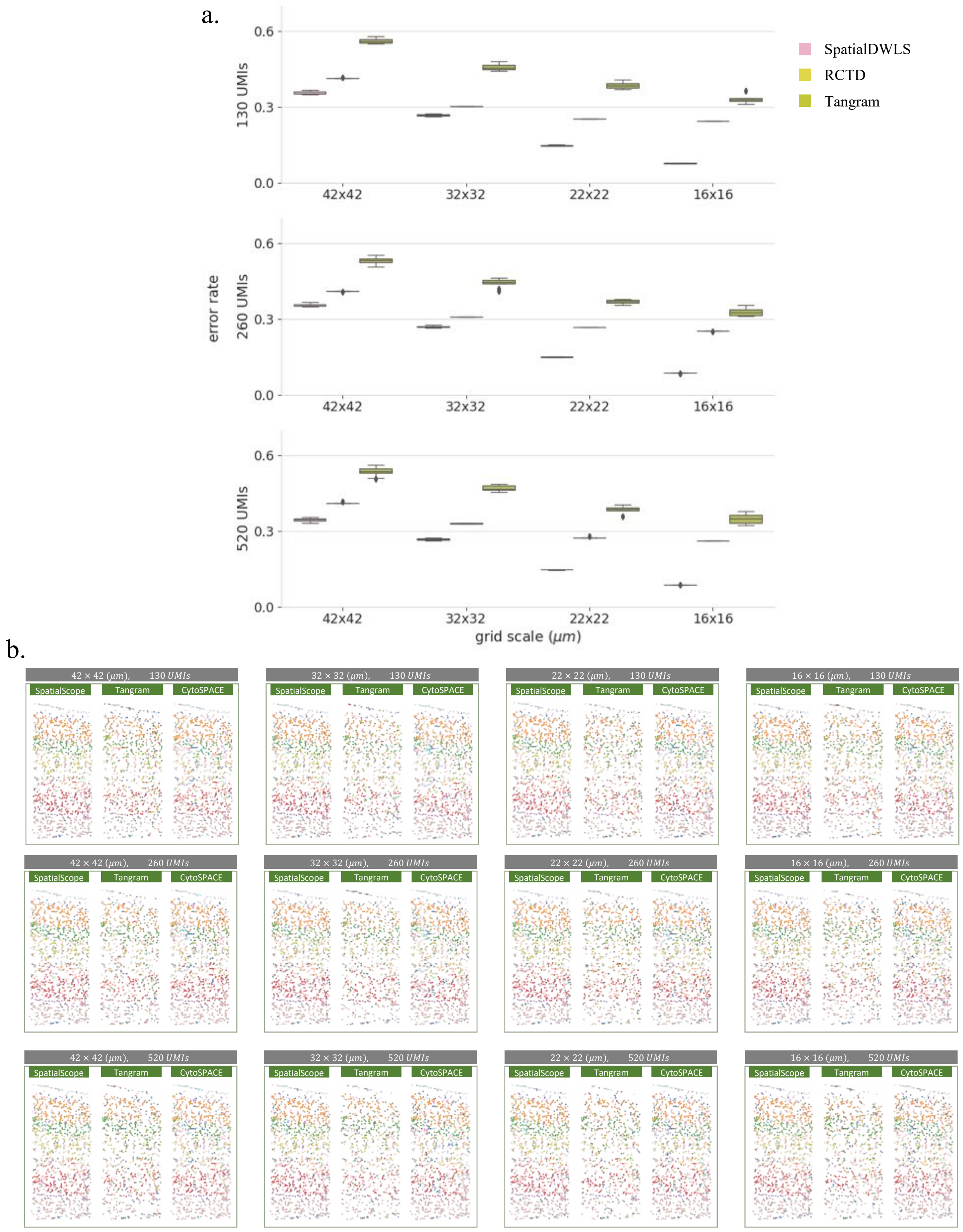

Figure S2: Comparison of cell type identification performance under more simulation settings. **a**, Summary of cell type identification results by error rates of the compared methods under different combination scenarios of grid scales and UMIs. Each box plot ranges from the third and first quartiles with the median as the horizontal line, while whiskers represent 1.5 times the interquartile range from the lower and upper bounds of the box. $n = 10$ is the number of experiments replicates for all grid scales. **b**, The cell type identification results of SpatialScope, Tangram and CytoSPACE. Source data are provided as a Source Data file.

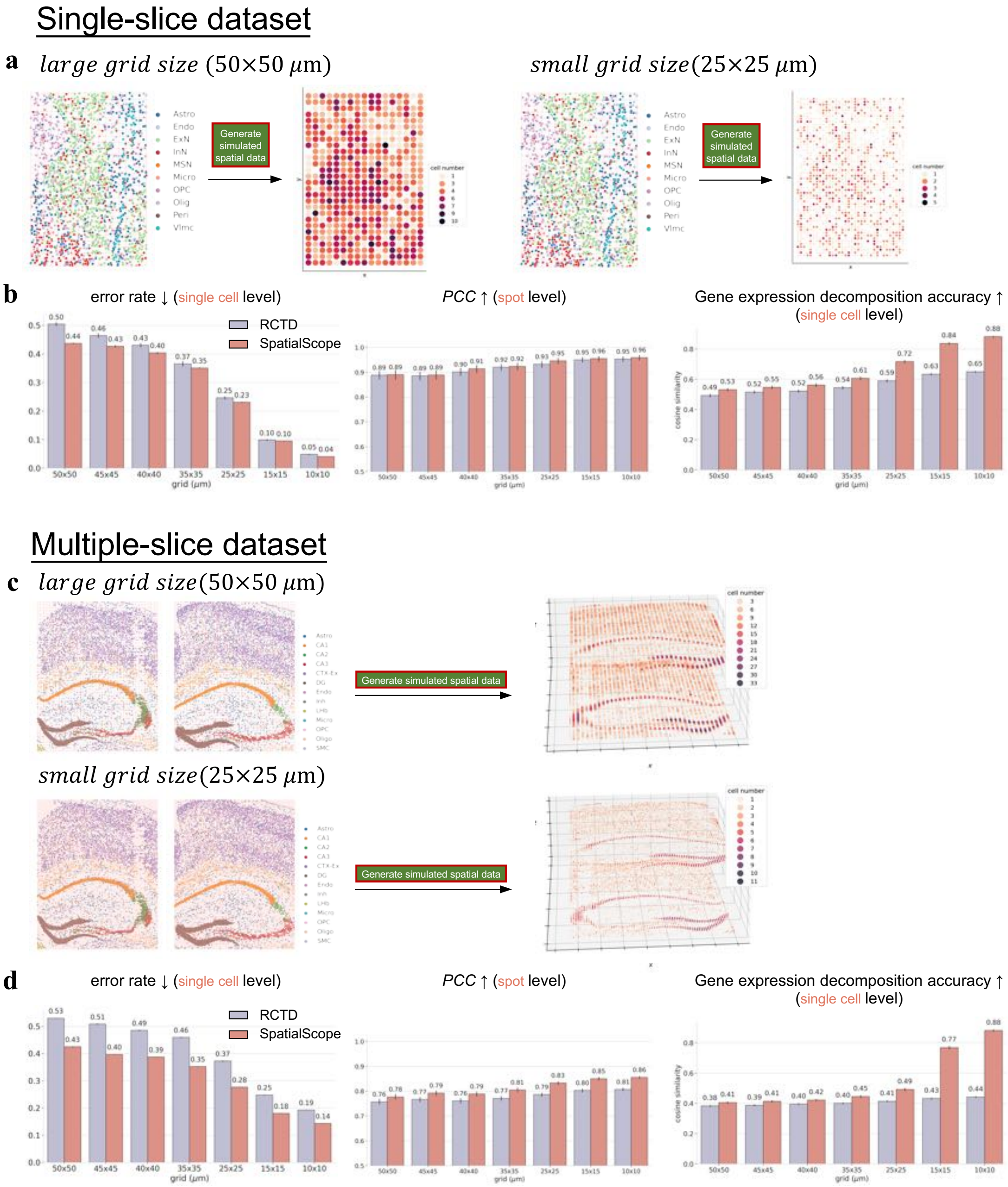
Single-slice dataset
a
large grid size (50×50 μm)
small grid size (25×25 μm)
Astro
Endo
ExN
InN
MSN
Micro
OPC
Olig
Peri
Vlmc
Generate simulated spatial data
cell number
b
error rate ↓ (single cell level)
RCTD
SpatialScope
grid (μm)
PCC ↑ (spot level)
Gene expression decomposition accuracy ↑ (single cell level)
cosine similarity
50x50
45x45
40x40
35x35
25x25
15x15
10x10
Multiple-slice dataset
c
large grid size (50×50 μm)
small grid size (25×25 μm)
Generate simulated spatial data
cell number
d
error rate ↓ (single cell level)
RCTD
SpatialScope
PCC ↑ (spot level)
Gene expression decomposition accuracy ↑ (single cell level)

Figure S3 (previous page): Performance comparison of SpatialScope and RCTD on simulated spatial transcriptomic data with different spot size. **a**, Schematic diagram for generating simulated ST data with large grid size (left) and small grid size (right) for single-slice data. **b**, Bar plot of error rate (left) across various grid scales from methods SpatialScope and RCTD on cell type identification task for single-slice data. Data are presented as mean values ±95% confidence intervals; $n = 10$ is the number of experiment replicates. Bar plot of or PCC (middle) across various grid scales from methods SpatialScope and RCTD on cell type identification task for single-slice data. Data are presented as mean values ±95% confidence intervals; $n = 468, 563, 683, 838, 1196, 1602, 1745$ is the number of spots for grid scales $50 \times 50, 45 \times 45, 40 \times 40, 35 \times 35, 25 \times 25, 15 \times 15, 10 \times 10$ respectively. Bar plot of cosine similarity across various grid scales from methods SpatialScope and RCTD on gene expression decomposition task for single-slice data (right). Data are presented as mean values ±95% confidence intervals; $n = 1768$ is the number of cells for grid scales $50 \times 50, 45 \times 45, 40 \times 40, 35 \times 35, 25 \times 25, 15 \times 15, 10 \times 10$ respectively. **c**, Schematic diagram for generating simulated ST data with large grid size (upper) and small grid size (lower) for multiple-slice data. **d**, Bar plot of error rate (left) across various grid scales from methods SpatialScope and RCTD on cell type identification task for multiple-slice data. Data are presented as mean values ±95% confidence intervals; $n = 10$ is the number of experiment replicates. Bar plot of PCC (middle) across various grid scales from methods SpatialScope and RCTD on cell type identification task for multiple-slice data. Data are presented as mean values ±95% confidence intervals; $n = 3623, 4345, 5229, 6407, 9744, 15010, 18199$ is the number of spots for grid scales $50 \times 50, 45 \times 45, 40 \times 40, 35 \times 35, 25 \times 25, 15 \times 15, 10 \times 10$ respectively. Bar plot of cosine similarity across various grid scales from methods SpatialScope and RCTD on gene expression decomposition task for multiple-slice data (right). Data are presented as mean values ±95% confidence intervals; $n = 1947, 1983, 1946, 2044, 1945, 1963, 1944$ is subsampled number of cells for grid scales $50 \times 50, 45 \times 45, 40 \times 40, 35 \times 35, 25 \times 25, 15 \times 15, 10 \times 10$ respectively. Source data are provided as a Source Data file.

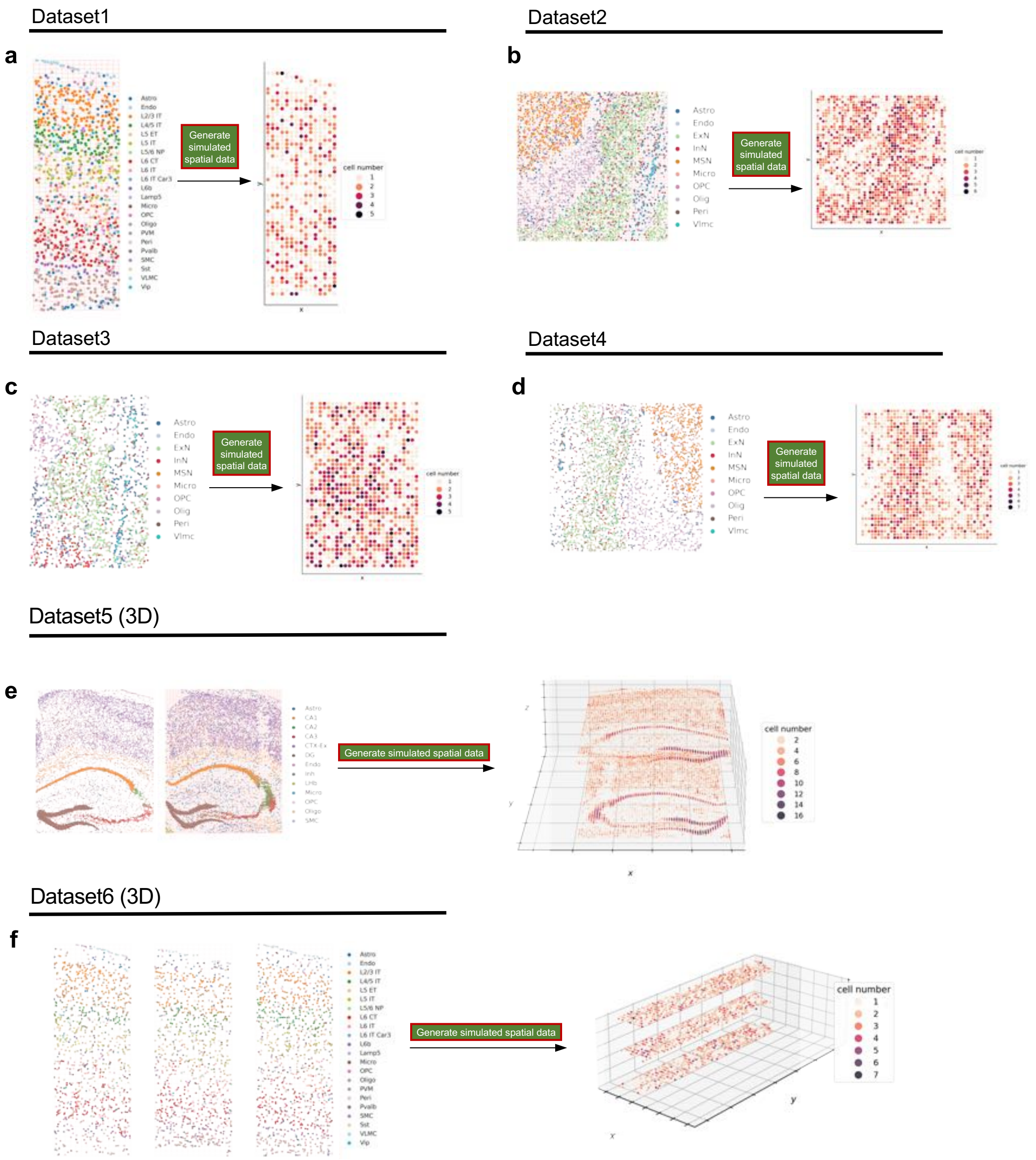

Figure S4: Benchmarking Datasets. Four single-slice and two multiple-slice ST datasets with annotated cell types for every single cell are used to generate simulated low-resolution spatial data. Figure shows the process of simulating spatial ST data. A spatial scatter plot displays the ground truth cell types at single-cell resolution (**a,e,f** left, **b,c,d** upper). Red dashed lines indicate the grids for aggregating cells to spots. Different color represents different cell types. After the aggregation, a scatter plot shows the simulated spots and the cell number of spots, and the Leiden clustering result (**a,e,f** right, **b,c,d** lower). Detailed descriptions of these benchmarking datasets are given in Supplementary Note 2.9.1.

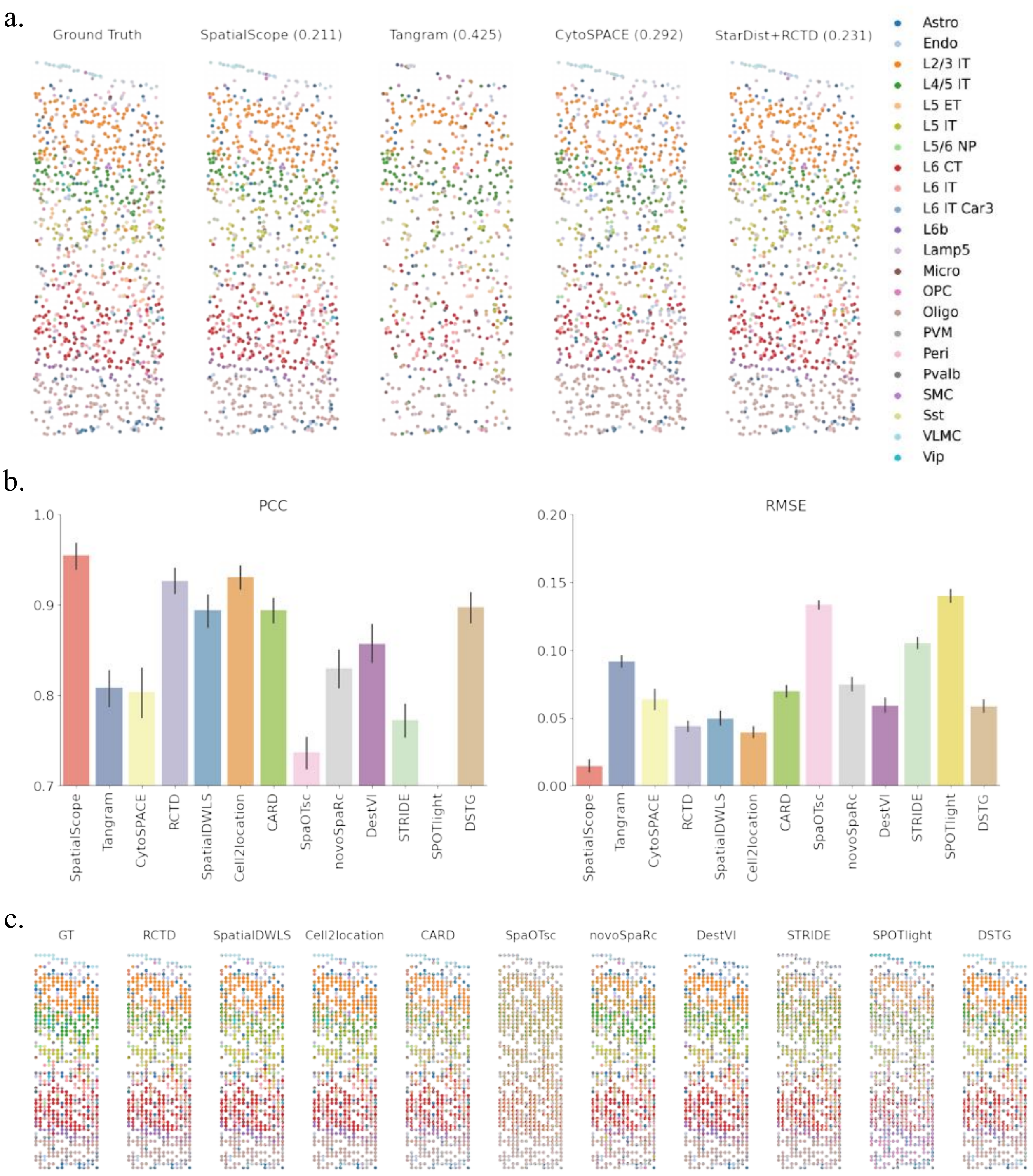

Figure S5: Comparison of cell type identification/deconvolution for Dataset 1. a, A spatial scatter plot displays identified single cell types on each cell location from ground truth and different methods. Each grid represents a simulated spot containing multiple cells. The value in brackets is the error rate of cell type recognition for each method. **b**, Summary of cell type deconvolution performance of the compared methods using PCC/RMSE between the inferred cell-type composition from different methods and the ground truth. Error bars represent the 95% confidence interval of PCC/RMS evaluated on $n = 599$ simulated spots. **c**, A spatial scatter pie plot displays ground truth and inferred cell-type composition on each spatial location from different methods. Source data are provided as a Source Data file.

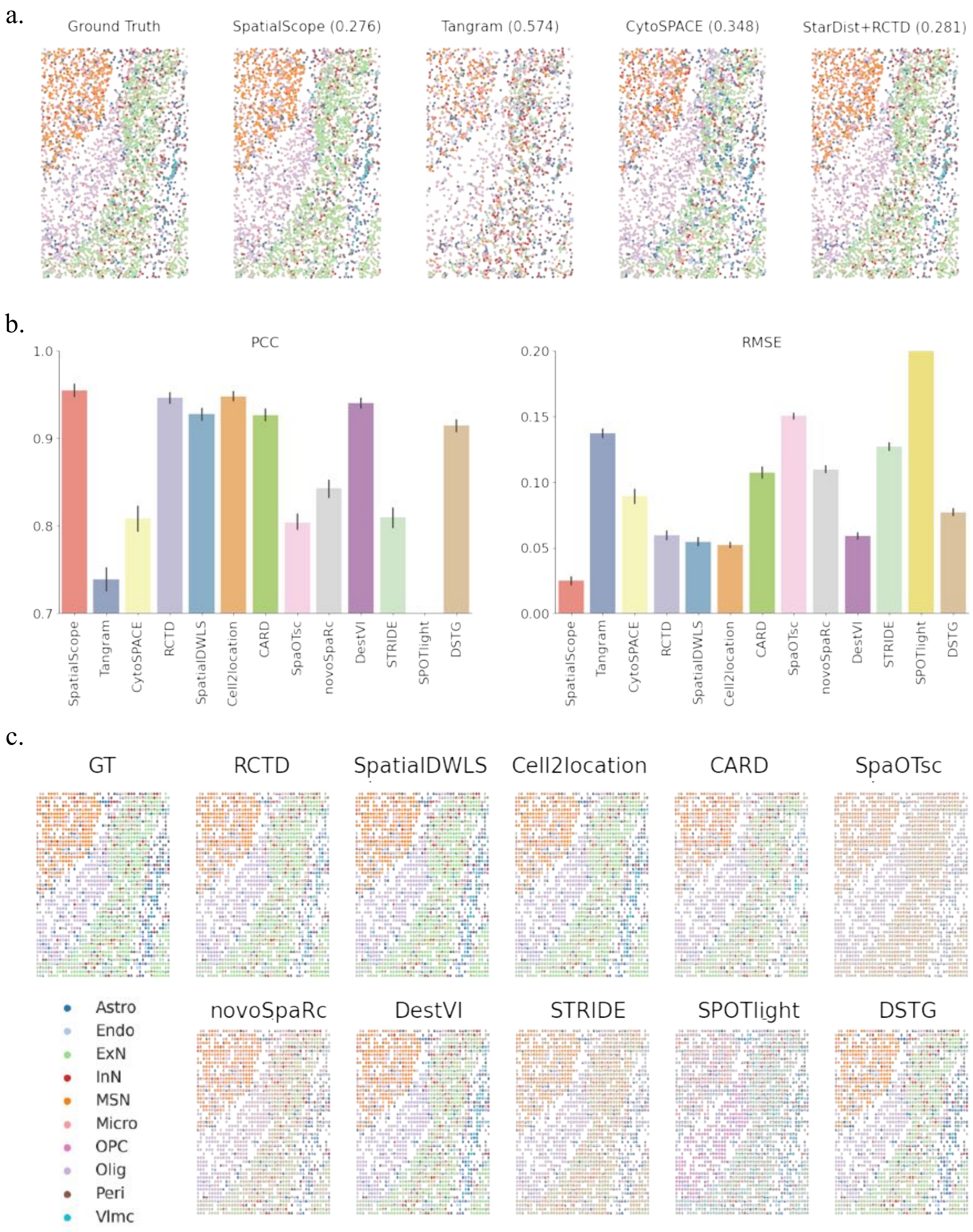

Figure S6: Comparison of cell type identification/deconvolution for Dataset 2. a, A spatial scatter plot displays identified single cell types on each cell location from ground truth and different methods. Each grid represents a simulated spot containing multiple cells. The value in brackets is the error rate of cell type recognition for each method. **b**, Summary of cell type deconvolution performance of the compared methods using PCC/RMSE between the inferred cell-type composition from different methods and the ground truth. Error bars represent the 95% confidence interval of PCC/RMSE evaluated on $n = 1753$ simulated spots. **c**, A spatial scatter pie plot displays ground truth and inferred cell-type composition on each spatial location from different methods. Source data are provided as a Source Data file.

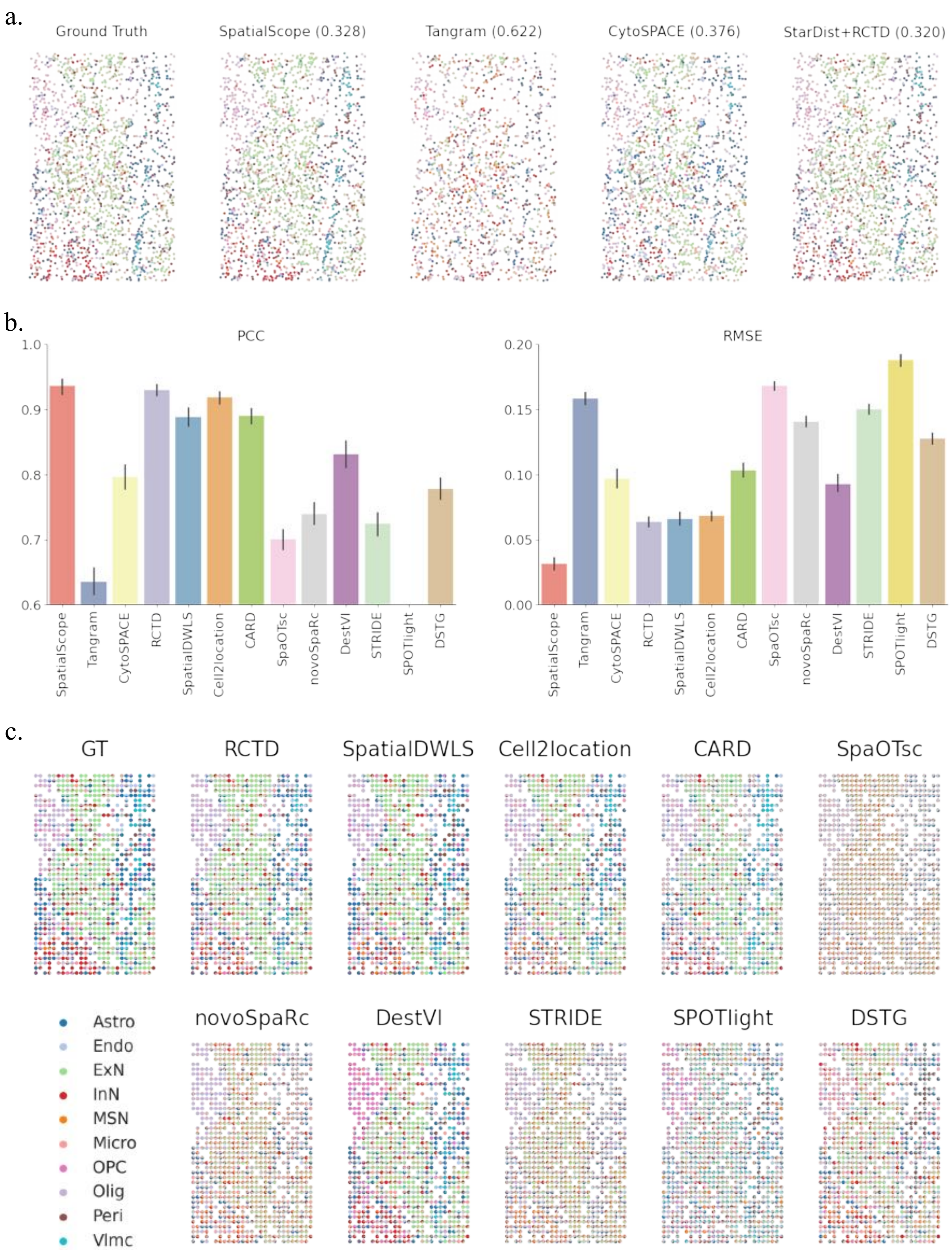

Figure S7: Comparison of cell type identification/deconvolution for Dataset 3. a, A spatial scatter plot displays identified single cell types on each cell location from ground truth and different methods. Each grid represents a simulated spot containing multiple cells. The value in brackets is the error rate of cell type recognition for each method. **b**, Summary of cell type deconvolution performance of the compared methods using PCC/RMSE between the inferred cell-type composition from different methods and the ground truth. Error bars represent the 95% confidence interval of PCC/RMSE evaluated on $n = 901$ simulated spots. **c**, A spatial scatter pie plot displays ground truth and inferred cell-type composition on each spatial location from different methods. Source data are provided as a Source Data file.

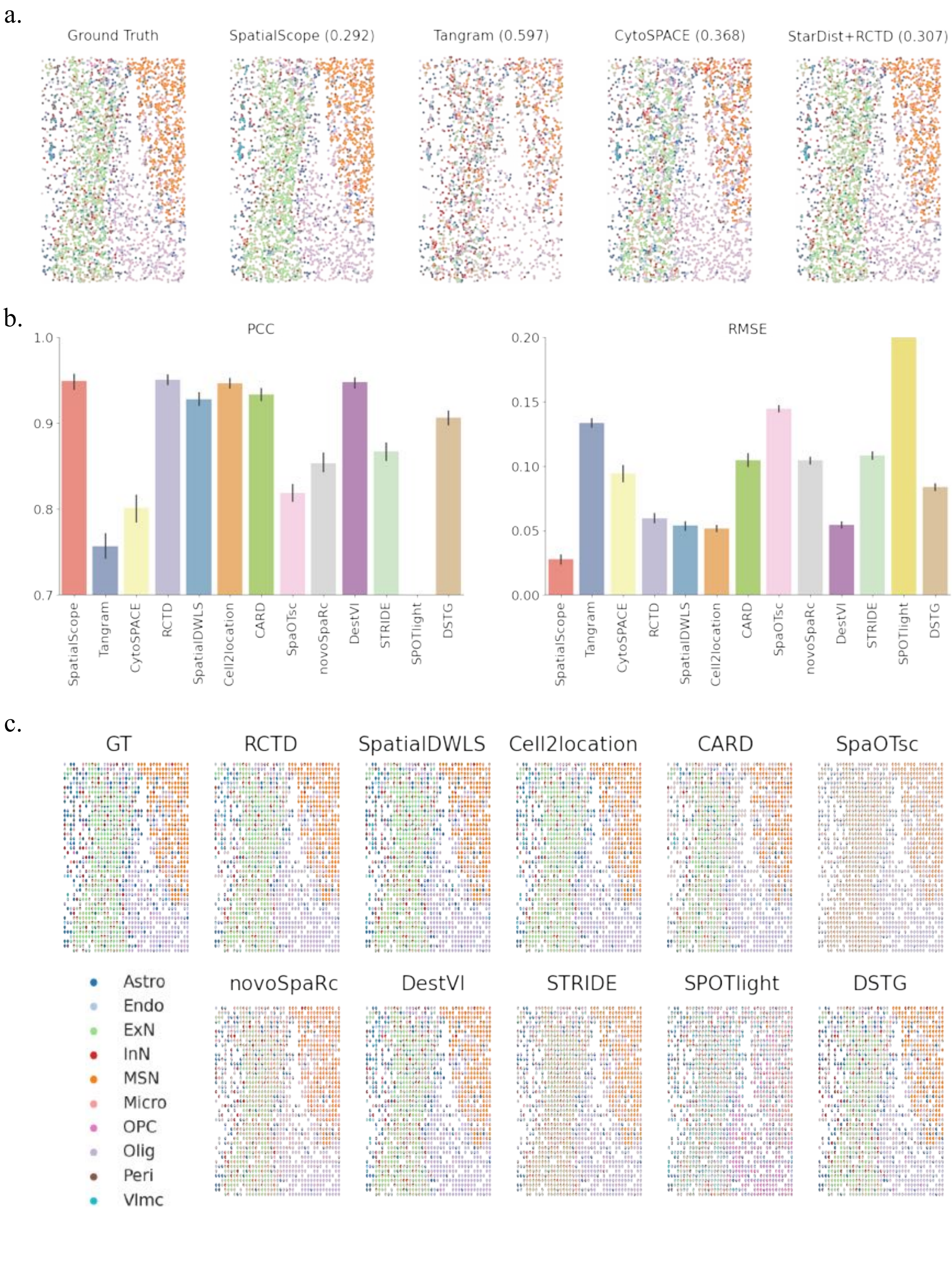
a.
Ground Truth
SpatialScope (0.292)
Tangram (0.597)
CytoSPACE (0.368)
StarDist+RCTD (0.307)
b.
PCC
1.0
0.9
0.8
0.7
RMSE
0.20
0.15
0.10
0.05
0.00
SpatialScope
Tangram
CytoSPACE
RCTD
SpatialDWLS
Cell2location
CARD
SpaOTsc
novoSpaRc
DestVI
STRIDE
SPOTlight
DSTG
c.
GT
RCTD
SpatialDWLS
Cell2location
CARD
SpaOTsc
novoSpaRc
DestVI
STRIDE
SPOTlight
DSTG
Astro
Endo
ExN
InN
MSN
Micro
OPC
Olig
Peri
Vlmc

Figure S8 (previous page): Comparison of cell type identification/deconvolution for Dataset 4. **a**, A spatial scatter plot displays identified single cell types on each cell location from ground truth and different methods. Each grid represents a simulated spot containing multiple cells. The value in brackets is the error rate of cell type recognition for each method. **b**, Summary of cell type deconvolution performance of the compared methods using PCC/RMSE between the inferred cell-type composition from different methods and the ground truth. Error bars represent the 95% confidence interval of PCC/RMSE evaluated on $n = 1359$ simulated spots. **c**, A spatial scatter pie plot displays ground truth and inferred cell-type composition on each spatial location from different methods. Source data are provided as a Source Data file.

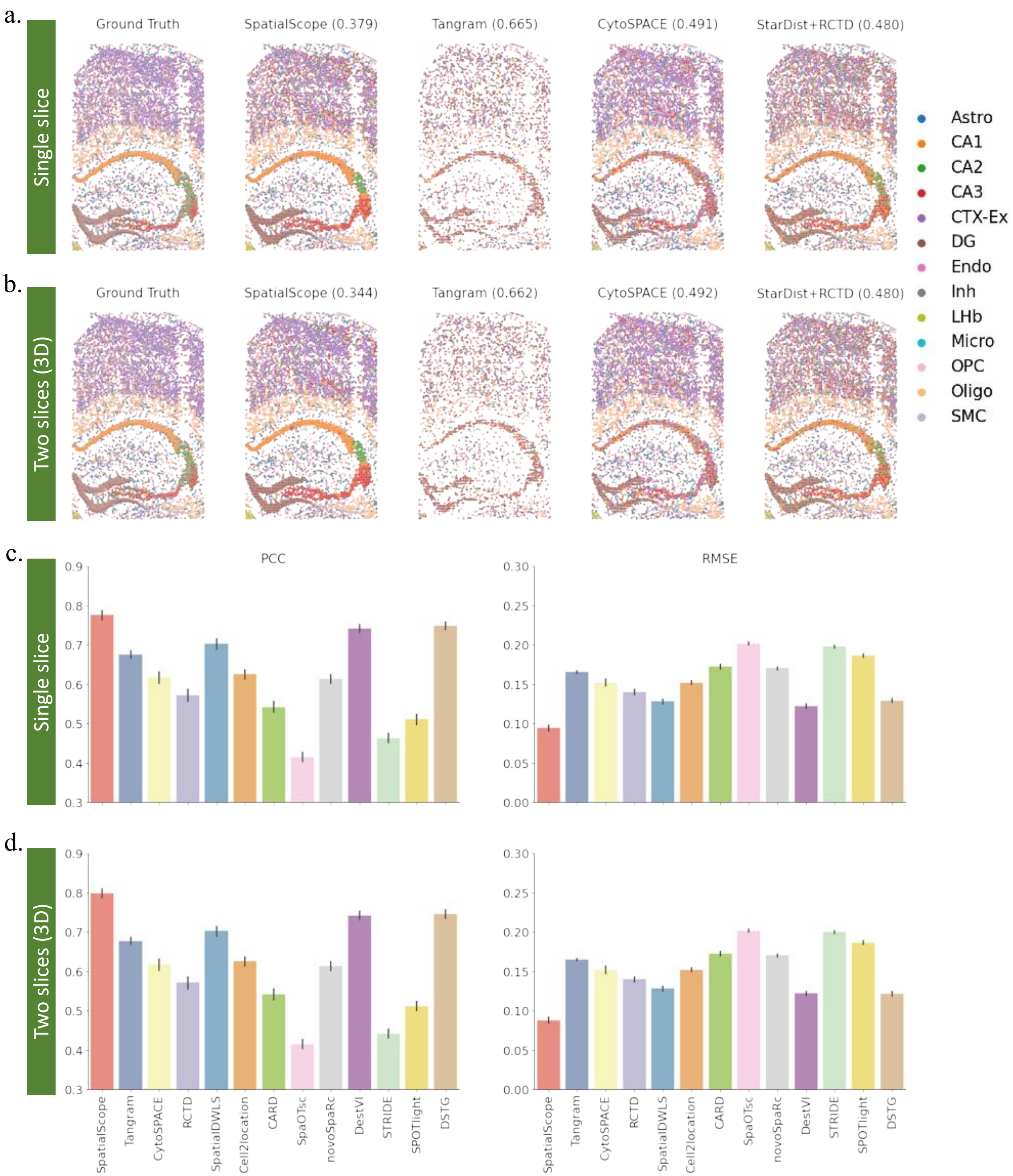

Figure S9: Comparison of cell type identification/deconvolution for slice 1 of Dataset 5. Spatial scatter plots display identified single cell types on each cell location from ground truth and different methods when single (**a**) or multiple (**b**) slices were used as input. Each grid represents a simulated spot containing multiple cells. The value in brackets is the error rate of cell type recognition for each method. Summary of cell type deconvolution performance of the compared methods using PCC/RMSE between the inferred cell-type composition from different methods and the ground truth when single (**c**) or multiple (**d**) slices were used as input. Error bars represent the 95% confidence interval of PCC/RMSE evaluated on $n = 3307$ simulated spots. Source data are provided as a Source Data file.

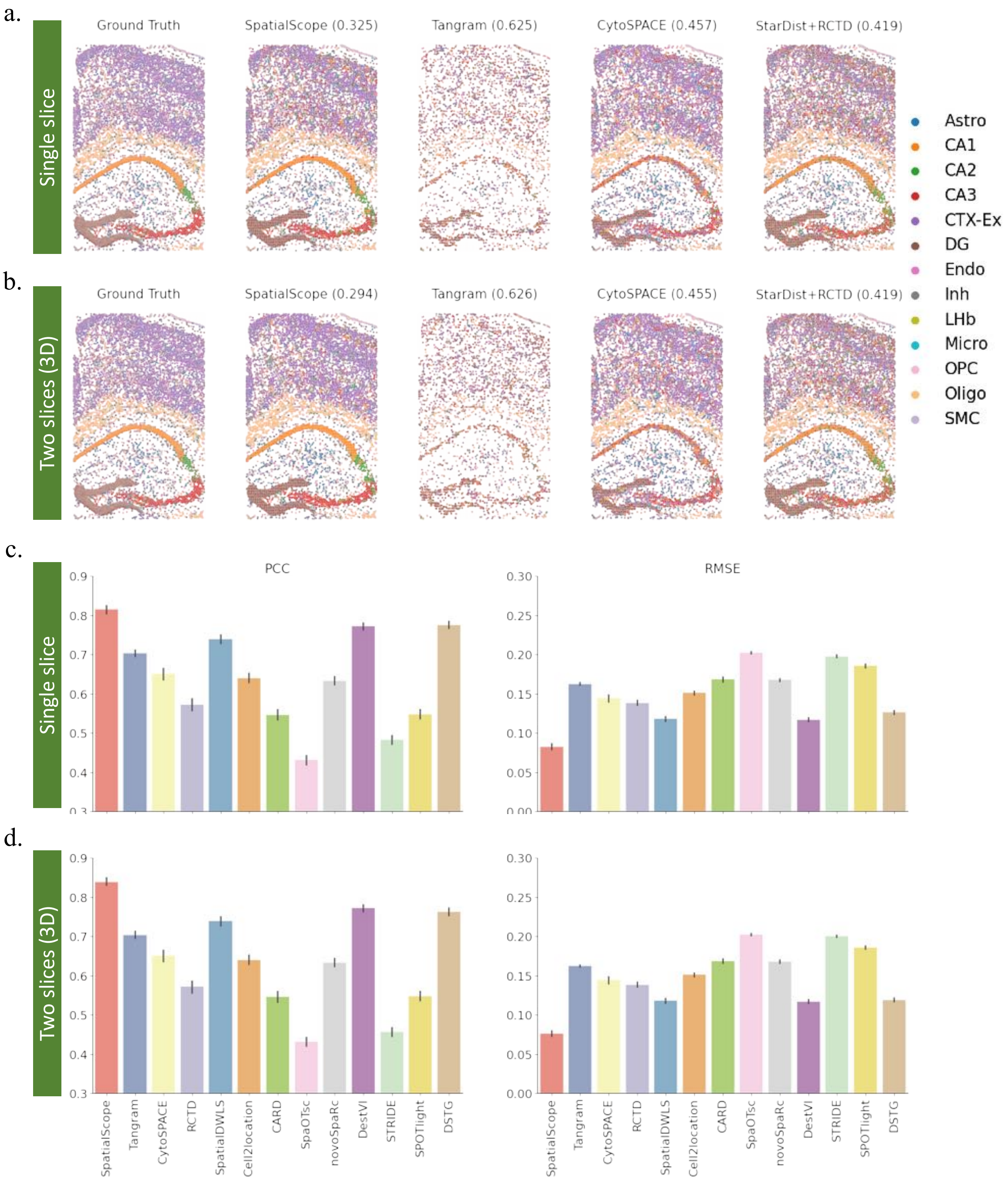

Figure S10: Comparison of cell type identification/deconvolution for slice 2 of Dataset 5. Spatial scatter plots display identified single cell types on each cell location from ground truth and different methods when single (**a**) or multiple (**b**) slices were used as input. Each grid represents a simulated spot containing multiple cells. The value in brackets is the error rate of cell type recognition for each method. Summary of cell type deconvolution performance of the compared methods using PCC/RMSE between the inferred cell-type composition from different methods and the ground truth when single (**c**) or multiple (**d**) slices were used as input. Error bars represent the 95% confidence interval of PCC/RMSE evaluated on $n = 3485$ simulated spots. Source data are provided as a Source Data file.

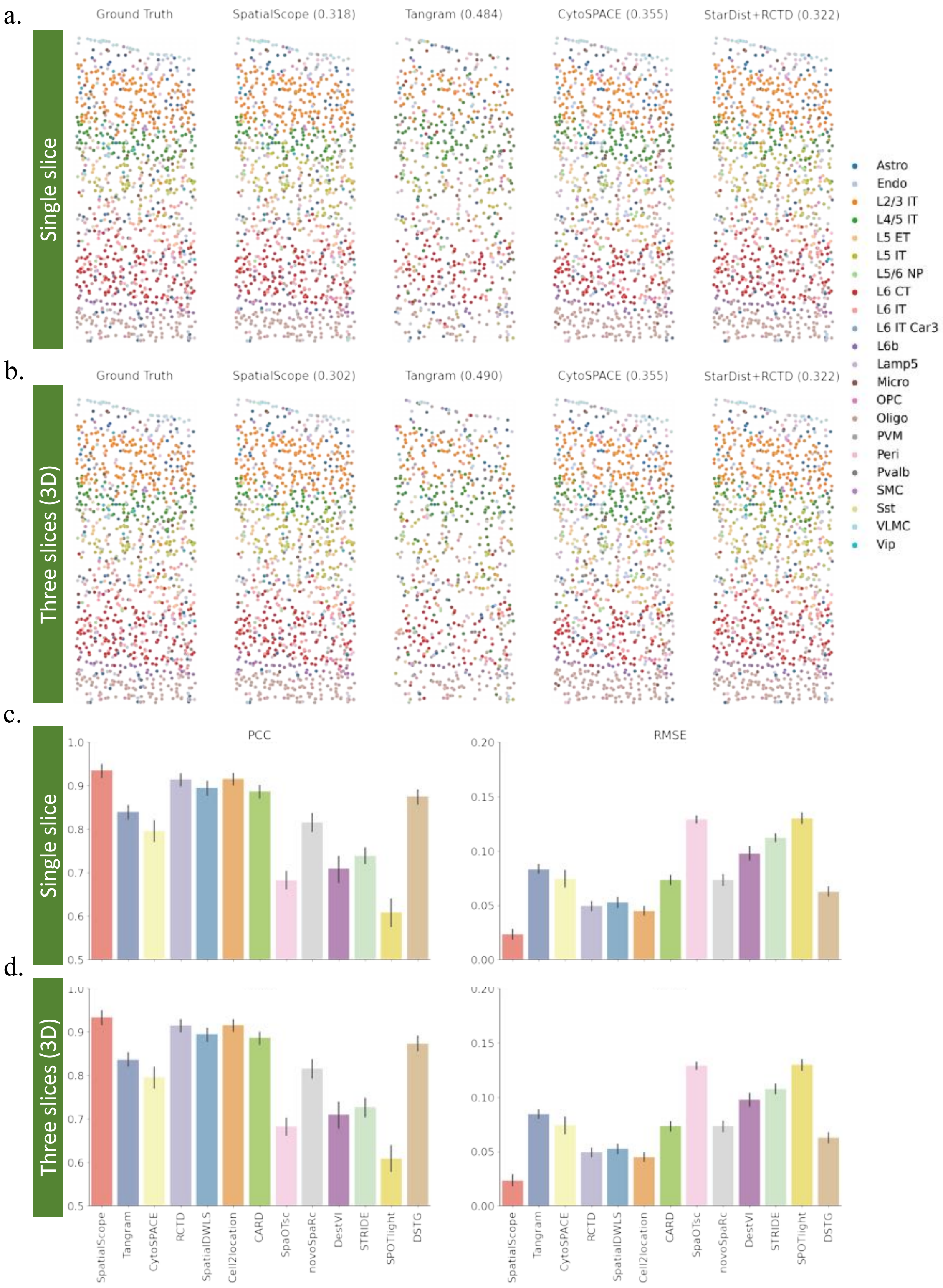

Figure S11: Comparison of cell type identification/deconvolution for slice 1 of Dataset 6. Spatial scatter plots display identified single cell types on each cell location from ground truth and different methods when single (**a**) or multiple (**b**) slices were used as input. Each grid represents a simulated spot containing multiple cells. The value in brackets is the error rate of cell type recognition for each method. Summary of cell type deconvolution performance of the compared methods using PCC/RMSE between the inferred cell-type composition from different methods and the ground truth when single (**c**) or multiple (**d**) slices were used as input. Error bars represent the 95% confidence interval of PCC/RMSE evaluated on $n = 505$ simulated spots. Source data are provided as a Source Data file.

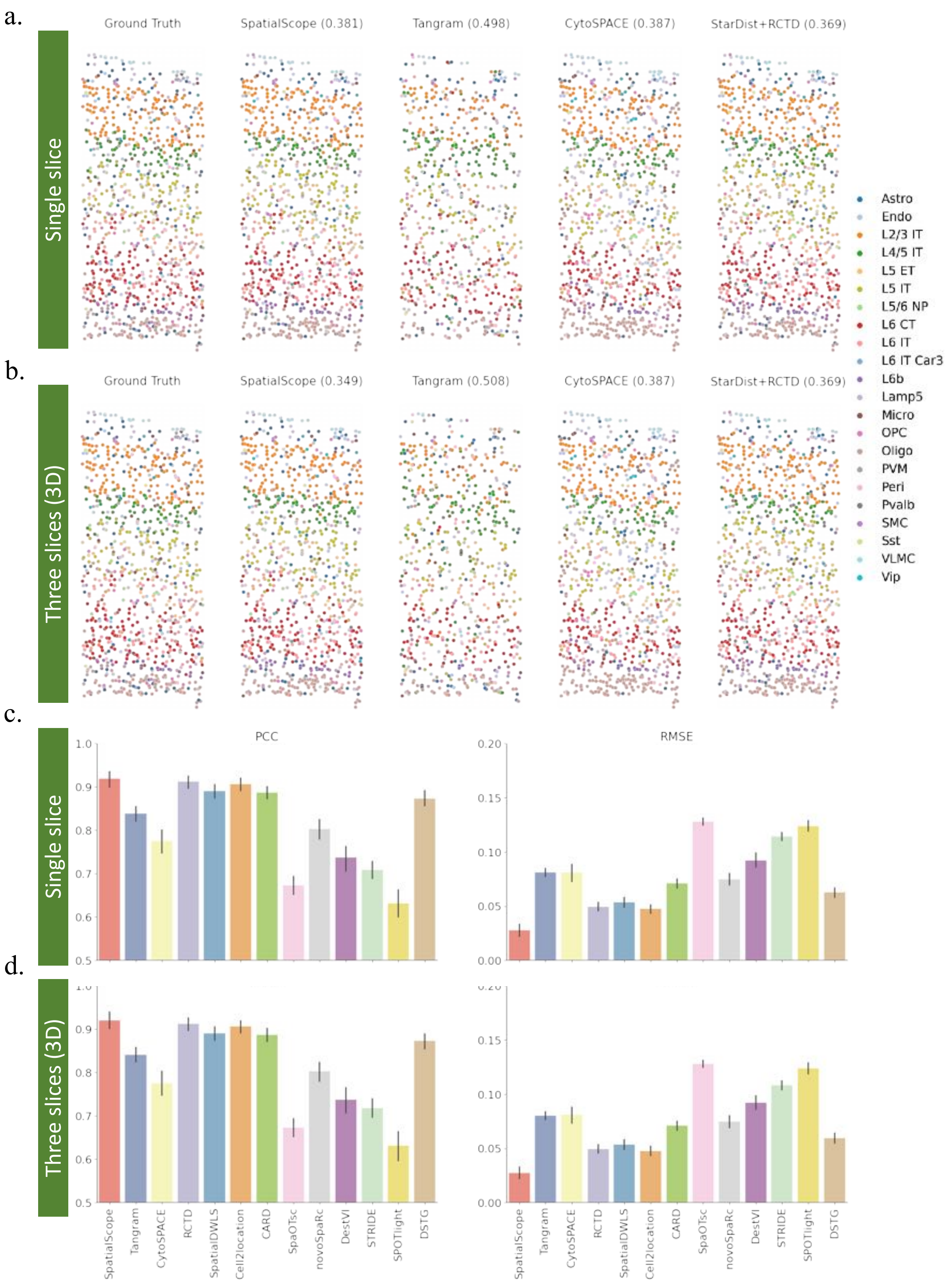

Figure S12: Comparison of cell type identification/deconvolution for slice 2 of Dataset 6. Spatial scatter plots display identified single cell types on each cell location from ground truth and different methods when single (**a**) or multiple (**b**) slices were used as input. Each grid represents a simulated spot containing multiple cells. The value in brackets is the error rate of cell type recognition for each method. Summary of cell type deconvolution performance of the compared methods using PCC/RMSE between the inferred cell-type composition from different methods and the ground truth when single (**c**) or multiple (**d**) slices were used as input. Error bars represent the 95% confidence interval of PCC/RMSE evaluated on $n = 486$ simulated spots. Source data are provided as a Source Data file.

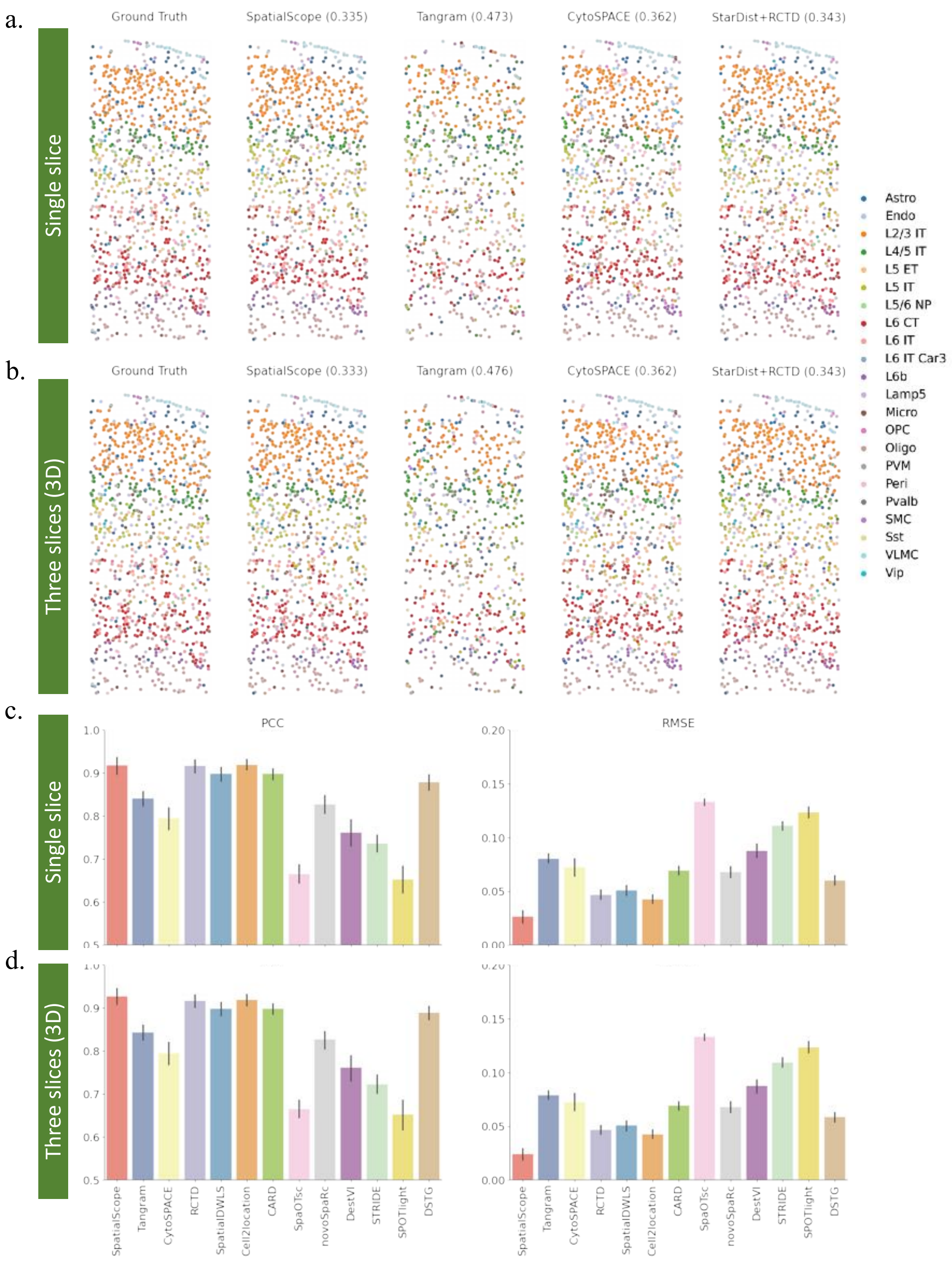

Figure S13: Comparison of cell type identification/deconvolution for slice 3 of Dataset 6. Spatial scatter plots display identified single cell types on each cell location from ground truth and different methods when single (**a**) or multiple (**b**) slices were used as input. Each grid represents a simulated spot containing multiple cells. The value in brackets is the error rate of cell type recognition for each method. Summary of cell type deconvolution performance of the compared methods using PCC/RMSE between the inferred cell-type composition from different methods and the ground truth when single (**c**) or multiple (**d**) slices were used as input. Error bars represent the 95% confidence interval of PCC/RMSE evaluated on $n = 503$ simulated spots. Source data are provided as a Source Data file.

Cell type identification

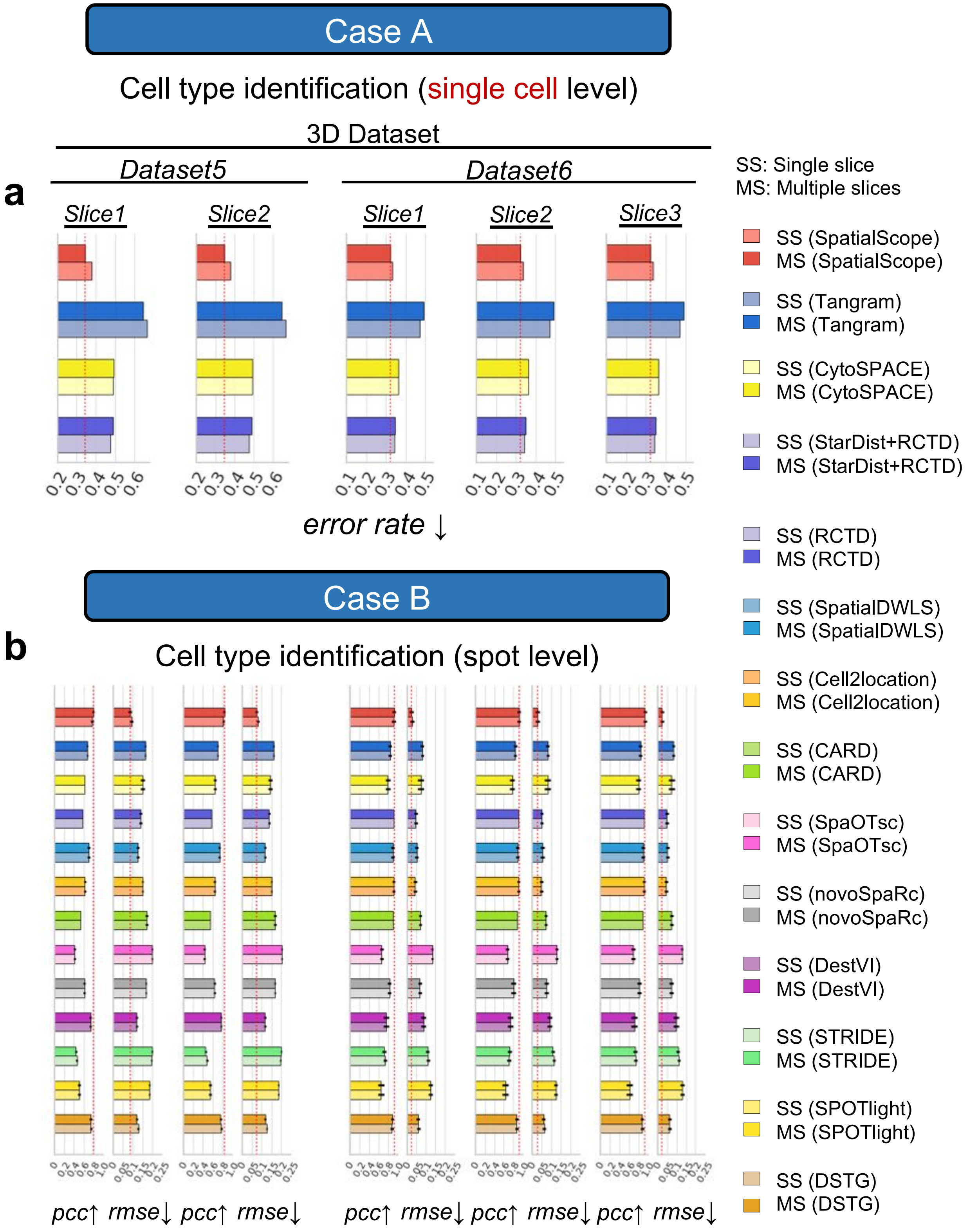

Figure S14 (previous page): Benchmarking of cell type identification based on the single-cell level (Case (a)) and the spot level (Case (b)) using multiple-slice datasets (Dataset 5-6). a, The bar plots of error rate of each method in inferring cell type label at the quesingle-cell level for two multiple-slice benchmarking datasets (Dataset 5-6). Two settings are considered. One is to apply methods to the single slice in the dataset one by one (Single slice), and another is to apply methods to all slices at once (Multiple slices). **b**, The bar plots of PCC and RMSE of each method in inferring cell type proportion at spot level for two multiple-slice benchmarking datasets (Dataset 5-6). Two settings are considered. One is to apply methods to the single slice in the dataset one by one (Single slice), and another is to apply methods to all slices at once (Multiple slices). Data are presented as mean values ±95% confidence intervals; $n = 3307, 3485$ is the number of spots for slice1 and slice2, respectively, in Dataset5. $n = 505, 486, 530$ is the number of spots for slice1, slice2, and slice3 respectively in Dataset6. Source data are provided as a Source Data file.

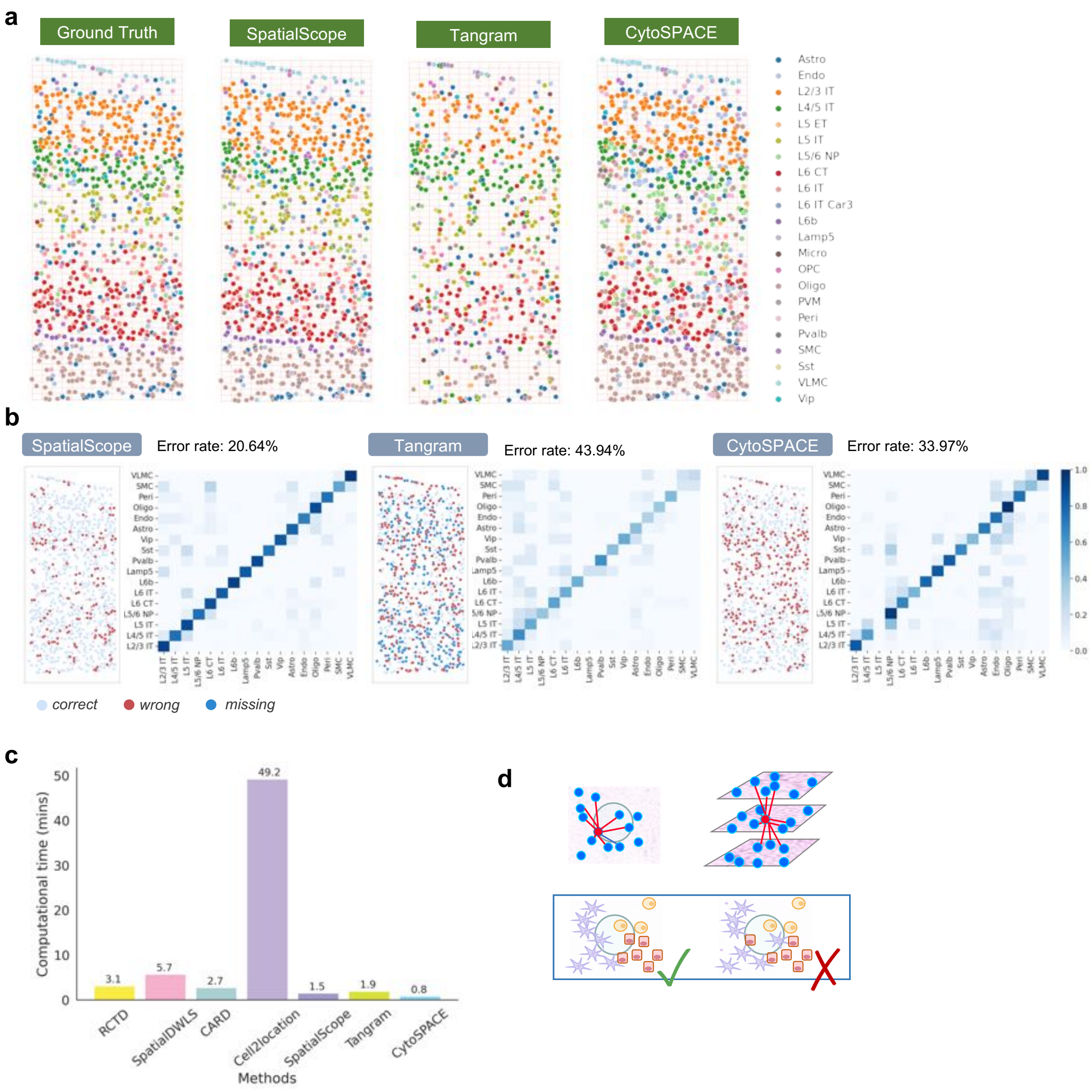

Figure S15: Simulated study of cell type identification. **a**, A spatial scatter plot displays identified single cell types on each cell location from ground truth and different methods. Each grid represents a simulated spot containing multiple cells. **b**, Scatter plot of single cells in simulated spatial data (left). Different colors show cells with correct/wrong cell type identification and cells missed by each method. Confusion matrix of true versus identified single cell types by different methods. Color in the confusion matrix represents the proportion of the cell type on the y-axis identified as the cell type on the x-axis. **c**, Computational times of SpatialScope and the compared methods when using Dataset 1 as the simulated spot-level ST data. **d**, Schematic diagram of SpatialScope utilizing spatial information by encouraging neighboring cells to belong to the same cell type within a single slice or across slices. Source data are provided as a Source Data file.

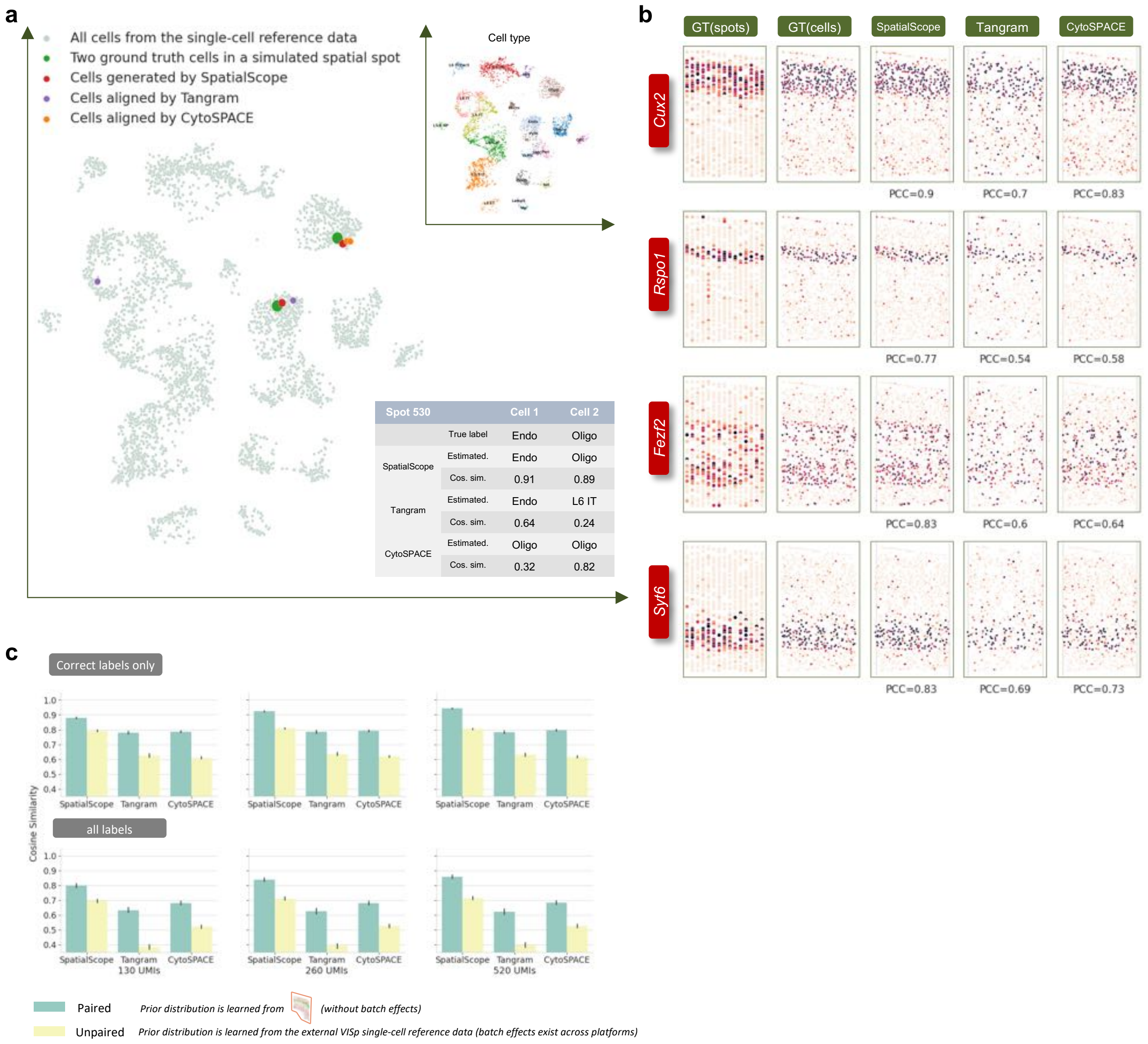

a
All cells from the single-cell reference data
Two ground truth cells in a simulated spatial spot
Cells generated by SpatialScope
Cells aligned by Tangram
Cells aligned by CytoSPACE
Cell type
Spot 530 | Cell 1 | Cell 2
True label | Endo | Oligo
SpatialScope Estimated. | Endo | Oligo
SpatialScope Cos. sim. | 0.91 | 0.89
Tangram Estimated. | Endo | L6 IT
Tangram Cos. sim. | 0.64 | 0.24
CytoSPACE Estimated. | Oligo | Oligo
CytoSPACE Cos. sim. | 0.32 | 0.82
b
GT(spots)
GT(cells)
SpatialScope
Tangram
CytoSPACE
Cux2
PCC=0.9
PCC=0.7
PCC=0.83
Rspo1
PCC=0.77
PCC=0.54
PCC=0.58
Fezf2
PCC=0.83
PCC=0.6
PCC=0.64
Syt6
PCC=0.83
PCC=0.69
PCC=0.73
c
Correct labels only
all labels
Cosine Similarity
SpatialScope
Tangram
CytoSPACE
130 UMIs
260 UMIs
520 UMIs
Paired
Prior distribution is learned from (without batch effects)
Unpaired
Prior distribution is learned from the external VISp single-cell reference data (batch effects exist across platforms)

Figure S16 (previous page): SpatialScope generates single-cell resolution gene expression profiles. **a**, The UMAP plot of gene expression decomposition example for a simulated spot with two cells from endothelial and oligodendrocyte, respectively. Dots of different colors represent gene expression profiles of two single cells decomposed from this simulated spot by different methods. The table shows the cosine similarity between the ground truth and decomposed gene expressions of two single cells in the simulated spot. In the table, "True label" represents ground truth cell type label; "Estimated." represents estimated cell type label by different methods; "Cos. sim." represents cosine similarity between ground truth gene expression profiles and estimated gene expression profiles by different methods. **b**, The spatial expression pattern of four layer's marker genes before and after applying SpatialScope's gene expression decomposition are displayed. SpatialScope decomposes spots to single-cell resolution, resulting in a refined spatial map of gene expression. **c**, The cosine similarities between the ground truth and decomposed single-cell level gene expression profiles of different methods are displayed. Two different single-cell reference data are used to evaluate the robustness of gene expression decomposition. Cosine similarity within correctly identified cell type label (top) or all cells (bottom) under different combination scenarios of UMI subsample rate and single-cell reference data are shown. Error bars represent the 95% confidence interval of cosine similarity evaluated on all $n = 940$ cells, $n = 730, 362, 620$ correctly labeled cells for SpatialScope, Tangram, CytoSPACE, respectively, in paired reference, 130UMI setting, $n = 746, 361, 620$ correctly labeled cells for SpatialScope, Tangram, CytoSPACE, respectively, in paired reference, 260UMI setting, $n = 740, 355, 624$ correctly labeled cells for SpatialScope, Tangram, CytoSPACE, respectively, in paired reference, 520UMI setting, $n = 604, 205, 680$ correctly labeled cells for SpatialScope, Tangram, CytoSPACE, respectively, in unpaired reference, 130UMI setting, $n = 621, 207, 677$ correctly labeled cells for SpatialScope, Tangram, CytoSPACE, respectively, in unpaired reference, 260UMI setting, $n = 644, 206, 686$ correctly labeled cells for SpatialScope, Tangram, CytoSPACE, respectively, in unpaired reference, 520UMI setting. Source data are provided as a Source Data file.

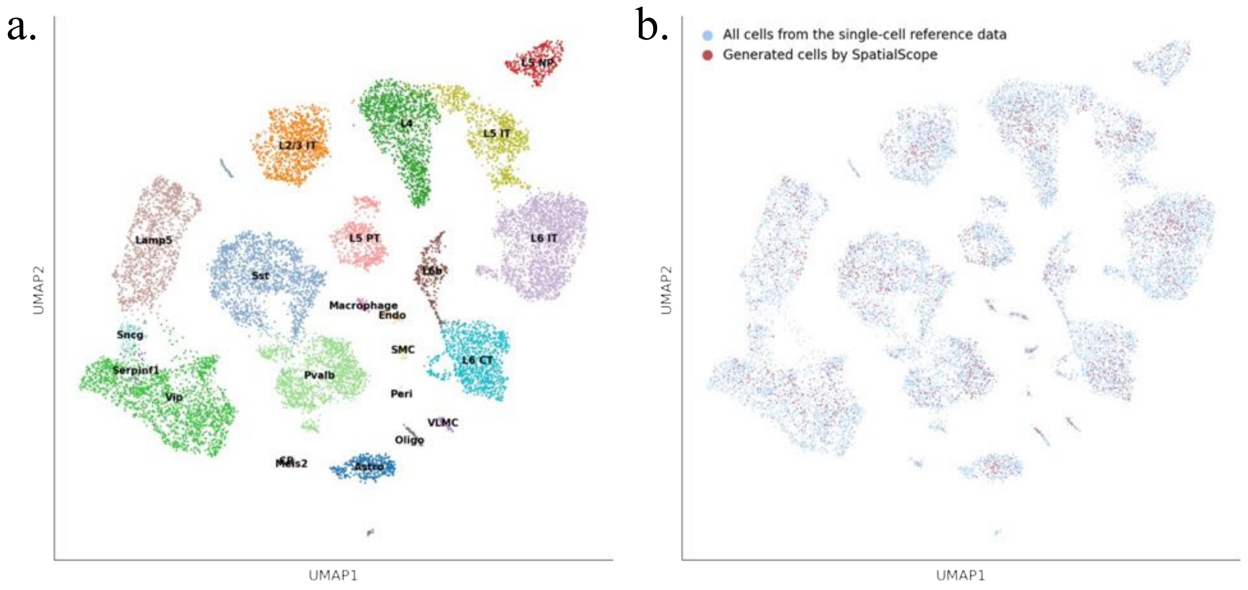

Figure S17: scRNA-seq reference of mouse brain cortex a, UMAP plot of scRNA-seq reference data with cell type annotations. **b**, UMAP plot of true cells and cells sampled from the learned distribution.

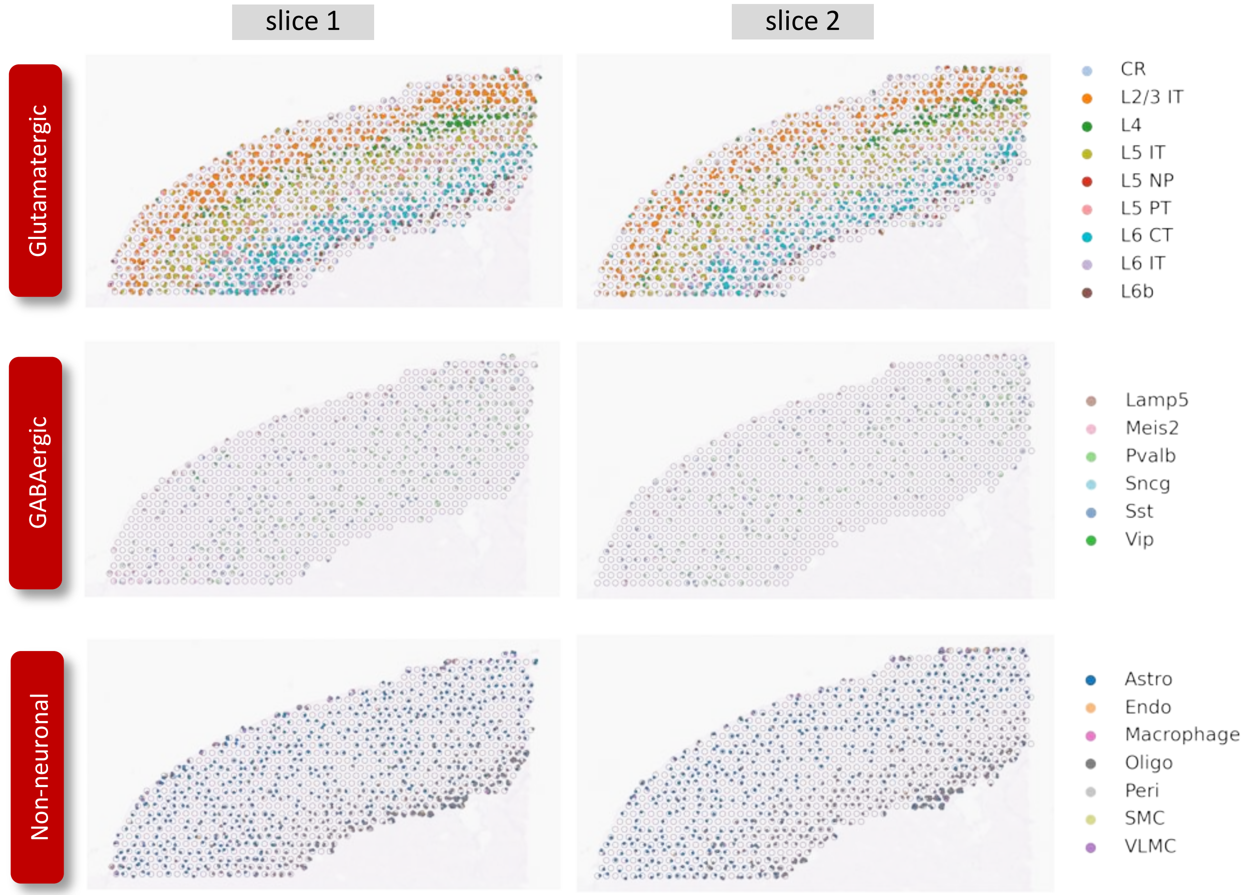

Figure S18: Cell type identification results by SpatialScope for the stacked 3D Visium mouse brain cortex data. Cell type identification results for slice 1 and slice2.

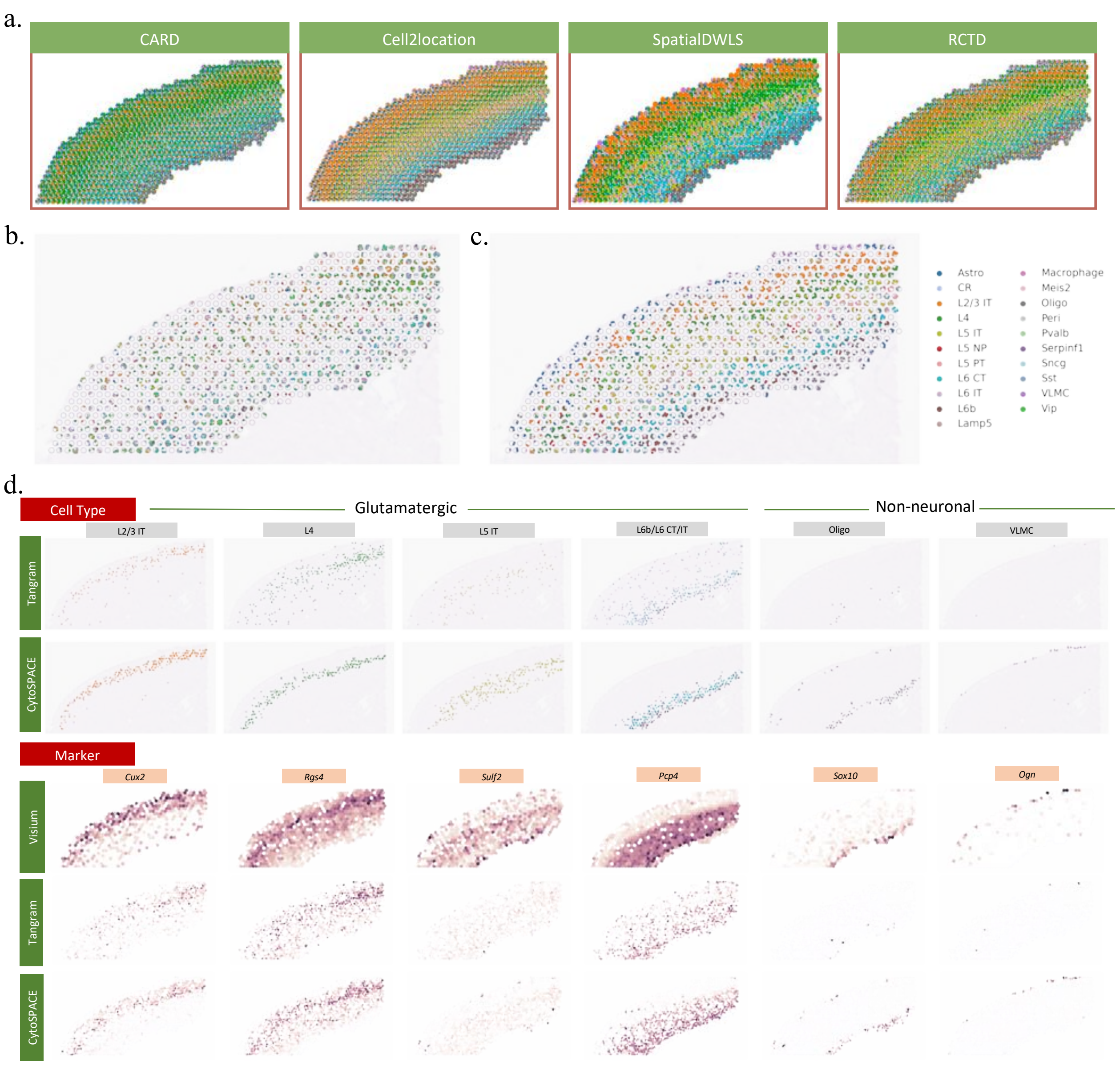

Figure S19: Visium mouse brain cortex's cell type identification and gene expression decomposition results by the compared methods. a, Cell type deconvolution results by spatialDWLS, RCTD, CARD, Cell2location. RCTD and Cell2location coarsely depicted the multi-layer structure, while CARD mistakenly assign many cells to the L4 layer and spatialDWLS did not provide clear boundaries across the layers. **b-c**, Cell type identification at single-cell resolution by Tangram and CytoSPACE. The canonical cortical four-layer structure is almost indistinguishable for Tangram due to missing cells caused by the soft regularized of cell number in each spot. CytoSPACE roughly reconstructed the four main layers but over-smoothed the cell type organization. For example, the right upper layer of the cortex was predicted to be Astrocytes only, while it should contain multiple cell types. **d**, The spatial cell type organizations (top) inferred by Tangram/CytoSPACE and Visium-measured spot-level (middle) and SpatialScope/CytoSPACE-decomposed single-cell level (bottom) expressions of a few marker genes.

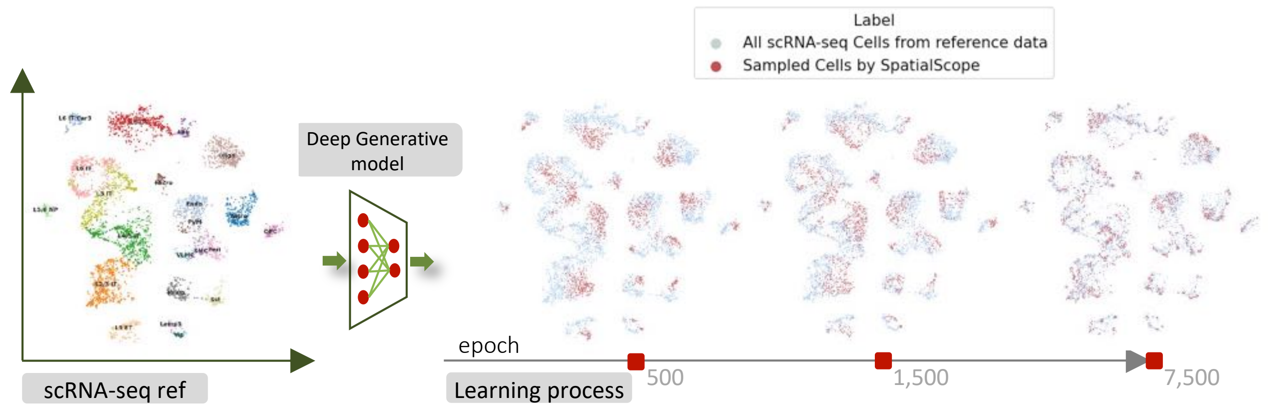

Figure S20: The UMAP plots of single-cell reference data and learned distribution at 500 and 7500 epochs. SpatialScope uses score-based generative modeling to learn the distribution of single-cell reference data.

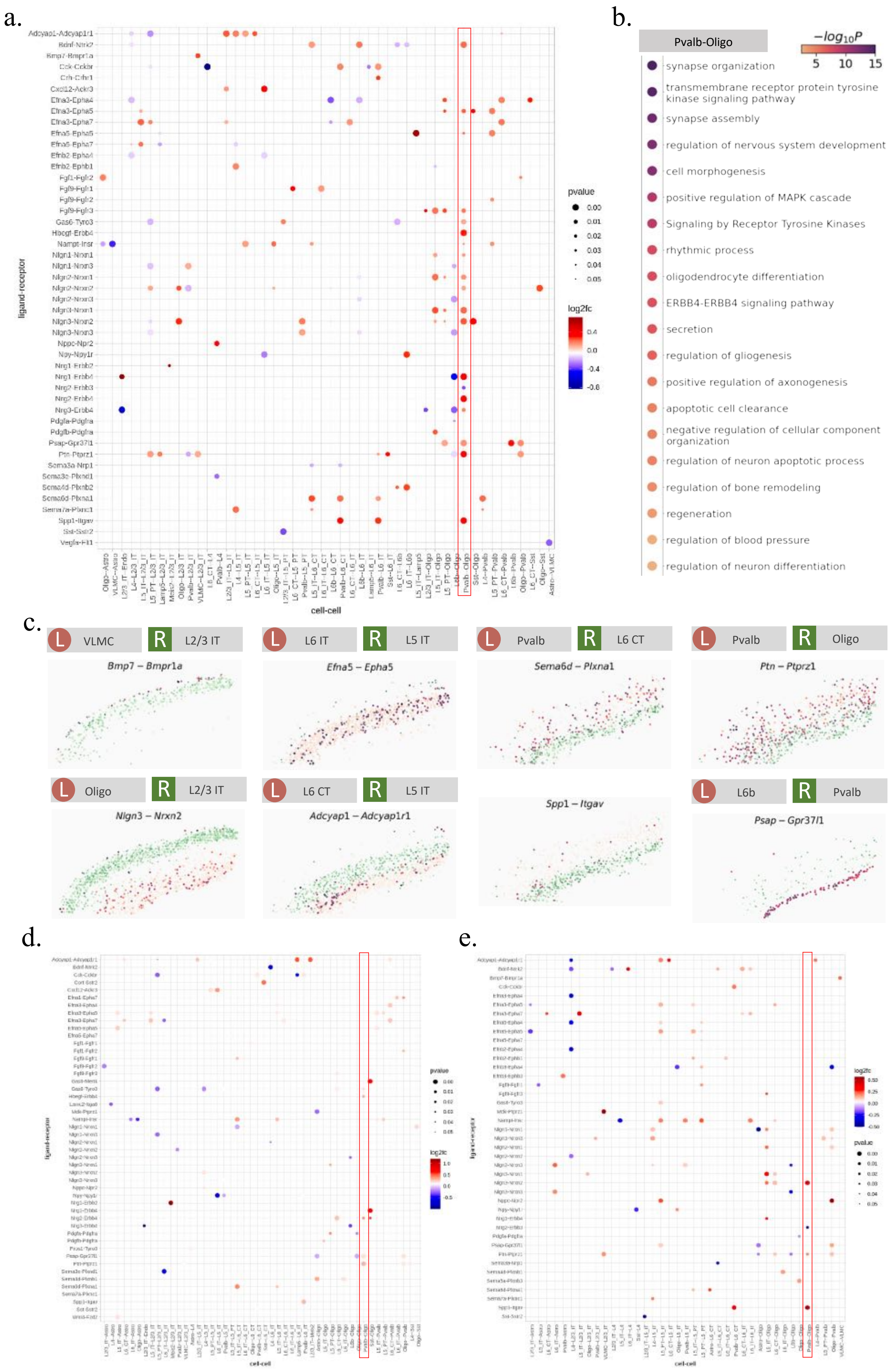
a.
b.
c.
d.
e.
ligand-receptor
cell-cell
pvalue
0.00
0.01
0.02
0.03
0.04
0.05
log2fc
Pvalb-Oligo
$-log_{10}P$
5
10
15
synapse organization
transmembrane receptor protein tyrosine kinase signaling pathway
synapse assembly
regulation of nervous system development
cell morphogenesis
positive regulation of MAPK cascade
Signaling by Receptor Tyrosine Kinases
rhythmic process
oligodendrocyte differentiation
ERBB4-ERBB4 signaling pathway
secretion
regulation of gliogenesis
positive regulation of axonogenesis
apoptotic cell clearance
negative regulation of cellular component organization
regulation of neuron apoptotic process
regulation of bone remodeling
regeneration
regulation of blood pressure
regulation of neuron differentiation
L VLMC
R L2/3 IT
Bmp7 – Bmpr1a
L L6 IT
R L5 IT
Efna5 – Epha5
L Pvalb
R L6 CT
Sema6d – Plxna1
L Pvalb
R Oligo
Ptn – Ptprz1
L Oligo
R L2/3 IT
Nlgn3 – Nrxn2
L L6 CT
R L5 IT
Adcyap1 – Adcyap1r1
Spp1 – Itgav
L L6b
R Pvalb
Psap – Gpr37l1

Figure S21 (previous page): Cell-cell interaction analysis in SpatialScope generated stacked 3D single-cell resolution ST mouse brain cortex data. **a**, Dot plot of ligand-receptor pairs that exhibit spatially resolved cell-cell communications when analyzing the 3D aligned ST data. p values were calculated under the null condition in the permuted data with the two-sided test. **b**, Significantly enriched Gene ontology biological processes determined with the Metascape web tool for the ligands and receptors from Pvalb and Oligo. **c** Visualization of a few representative cellular communications detected in the SpatialScope generated stacked 3D single-cell resolution ST mouse brain cortex data. **d**. Dot plot of ligand-receptor pairs that exhibit spatially resolved cell-cell communications when analyzing slice 1 only. p values were calculated under the null condition in the permuted data with the two-sided test. **e**. Dot plot of ligand-receptor pairs that exhibit spatially resolved cell-cell communications when analyzing slice 2 only. p values were calculated under the null condition in the permuted data with the two-sided test. Source data are provided as a Source Data file.

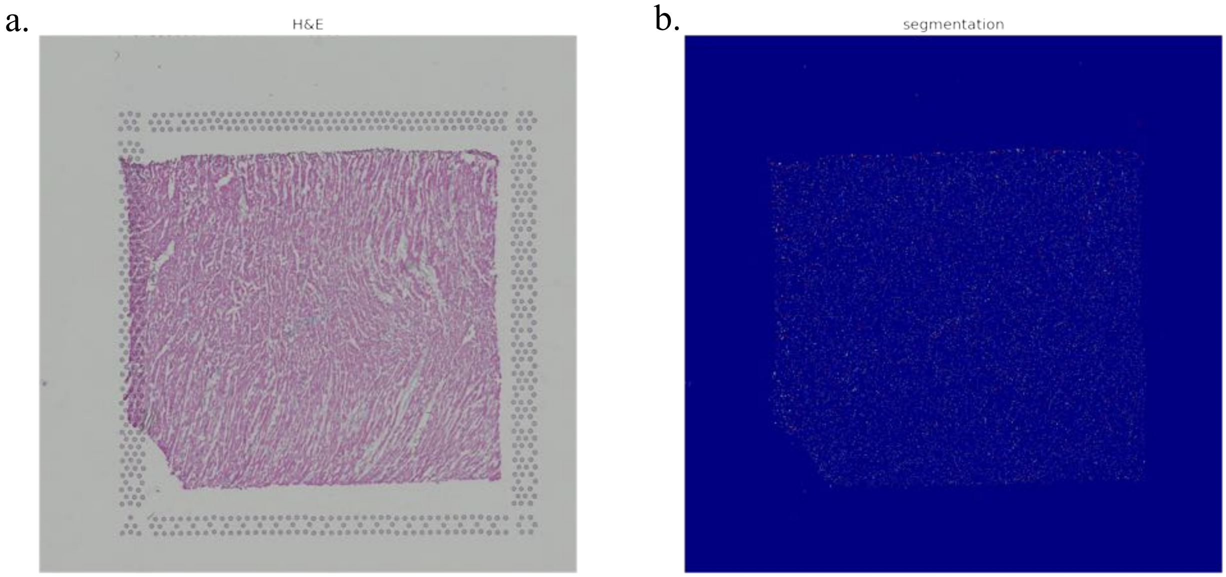

Figure S22: Nucleus segmentation result for 10X Visium heart data. **a**, The paired H&E stained histological image. **b**, The nucleus segmentation results

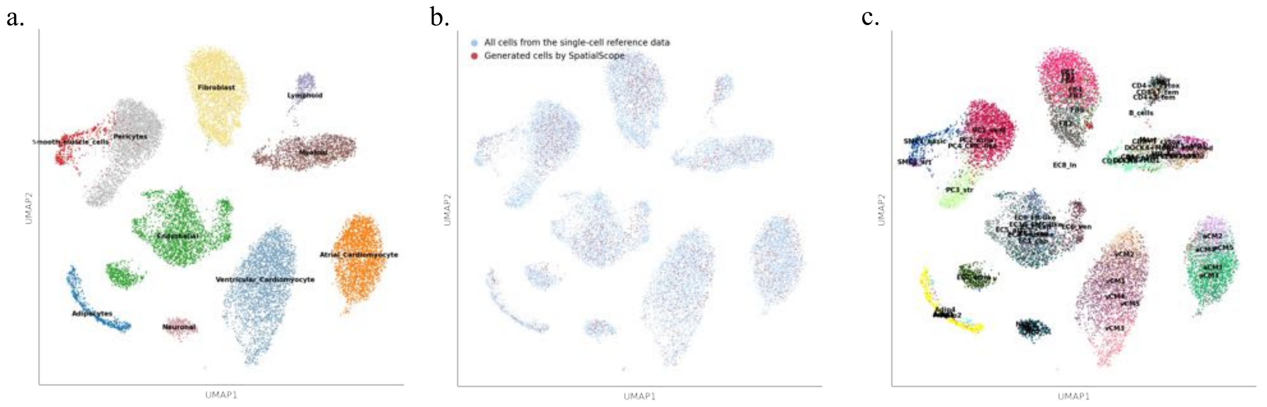

Figure S23: snRNA-seq reference of human heart a, UMAP plot of snRNA-seq reference data with cell type annotations. **b**, UMAP plot of true cells and cells sampled from the learned distribution, **c**, UMAP plot of snRNA-seq reference data with cell type subgroup annotations.

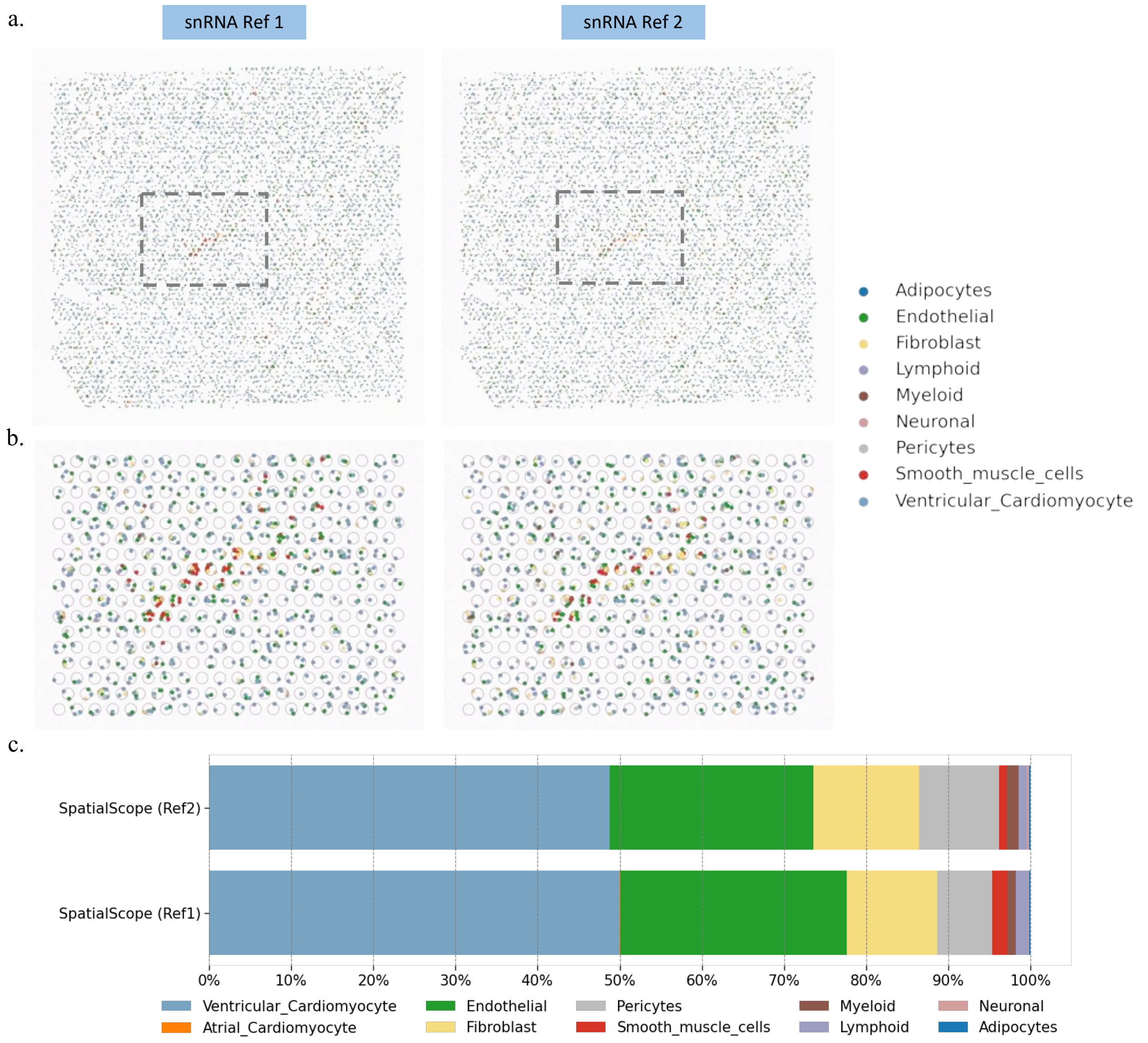

Figure S24: Visium heart data cell type identification results by SpatialScope when distinct snRNA-seq reference was used. a, Cell type identification at single-cell resolution for the whole slice. **b**, Cell type identification at single-cell resolution for ROI. **c**, Inferred cell type compositions across the whole slice when distinct snRNA-seq reference was used.

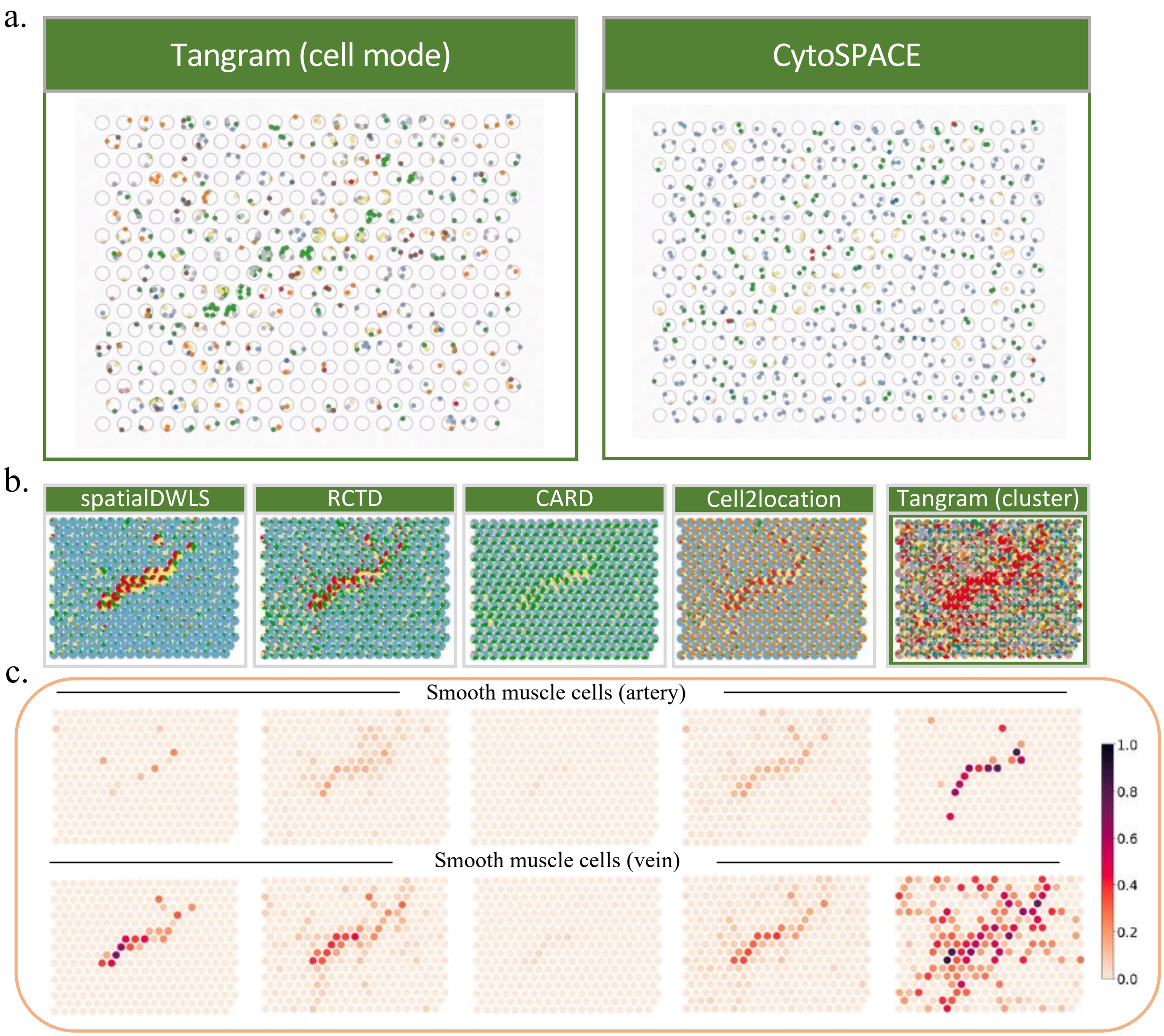

Figure S25: Visium heart data cell type identification results by the compared methods a, Cell type identification at single-cell resolution for ROI by Tangram (cell mode) and CytoSPACE. **b**, Cell type deconvolution results by spatialDWLS, RCTD, CARD, Cell2location and Tangram (cluster mode). **c**, Inferred cell type proportion for the two subgroups of SMC. We used the separated SMC cell type labels (SMC_artery, SMC_vein) in snRNA-seq reference rather than a unified SMC cell type label during the deconvolution, then we compared the estimated cell type proportion for these two subgroups to evaluate the discrimination abilities of the compared methods.

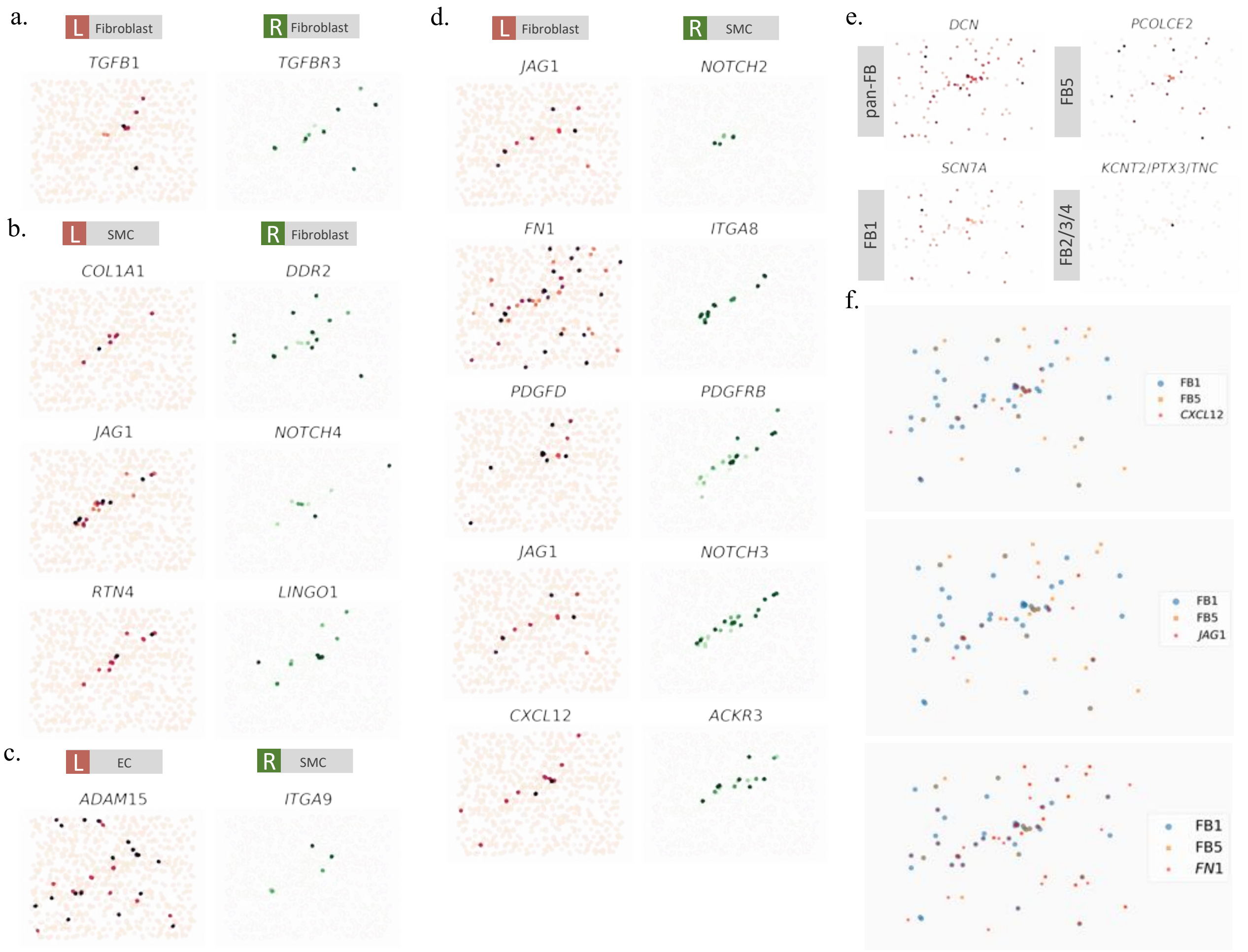

Figure S26: More cellular communications detected in the SpatialScope generated single-cell resolution human heart ST data. **a**, Visualization of molecular interactions between Fibroblast and Fibroblast. **b**, Visualization of molecular interactions between SMC and Fibroblast. **c**, Visualization of molecular interactions between EC and SMC. **d**, Visualization of molecular interactions between Fibroblast and SMC. **e**, Expression of Fibroblast marker genes in single-cell transcriptomes generated by SpatialScope. **f**, Spatial locations of subgroups (FB1, FB2) of Fibroblast.

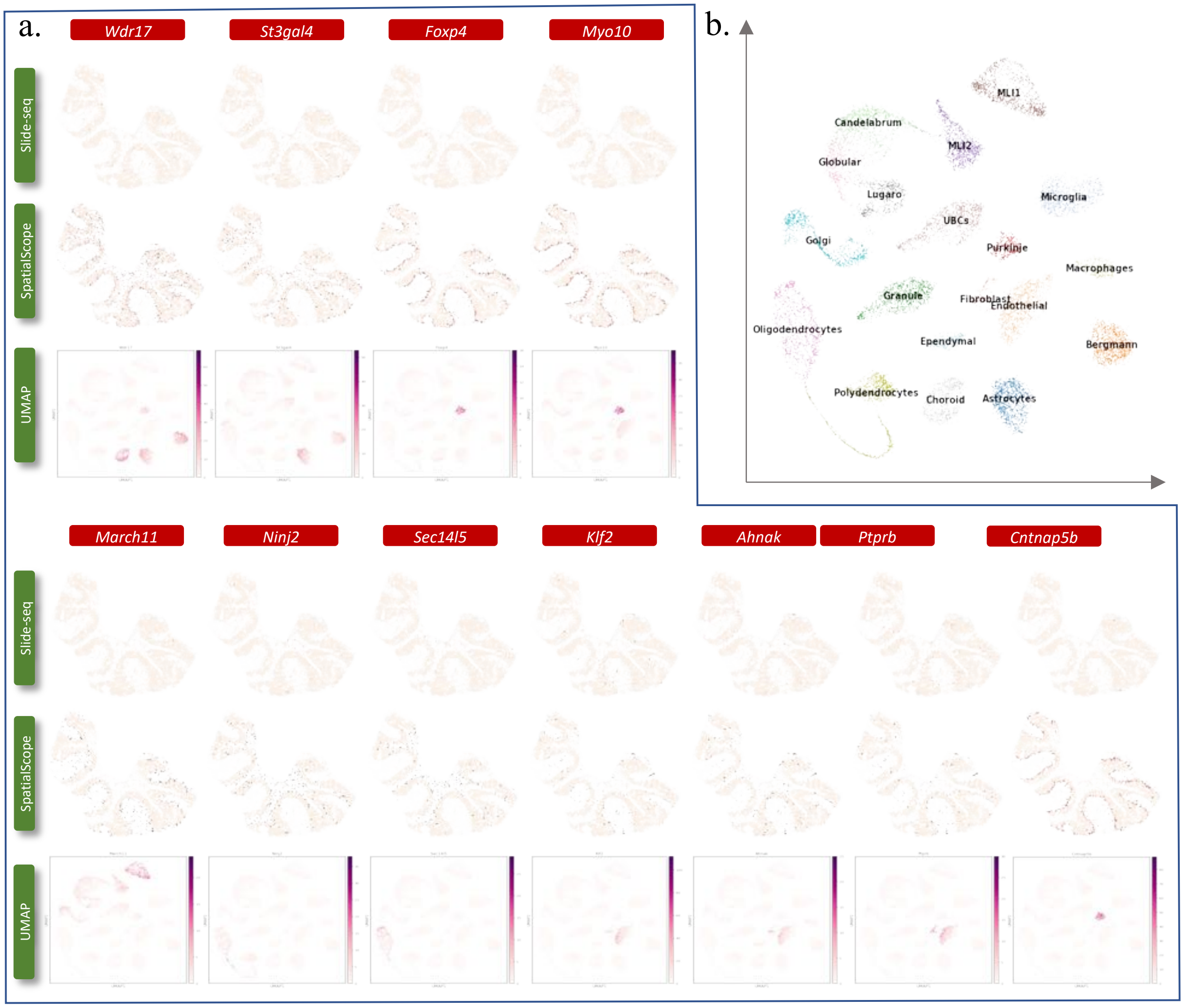

Figure S27: Dropouts corrections by SpatialScope in Slide-seq data a, Slide-seq measured (top) and SpatialScope corrected (middle) expressions of highly sparse marker genes. The marker gene expression signatures were displayed with UMAP plots. **b**, UMAP plot of snRNA-seq reference data with cell type annotations.

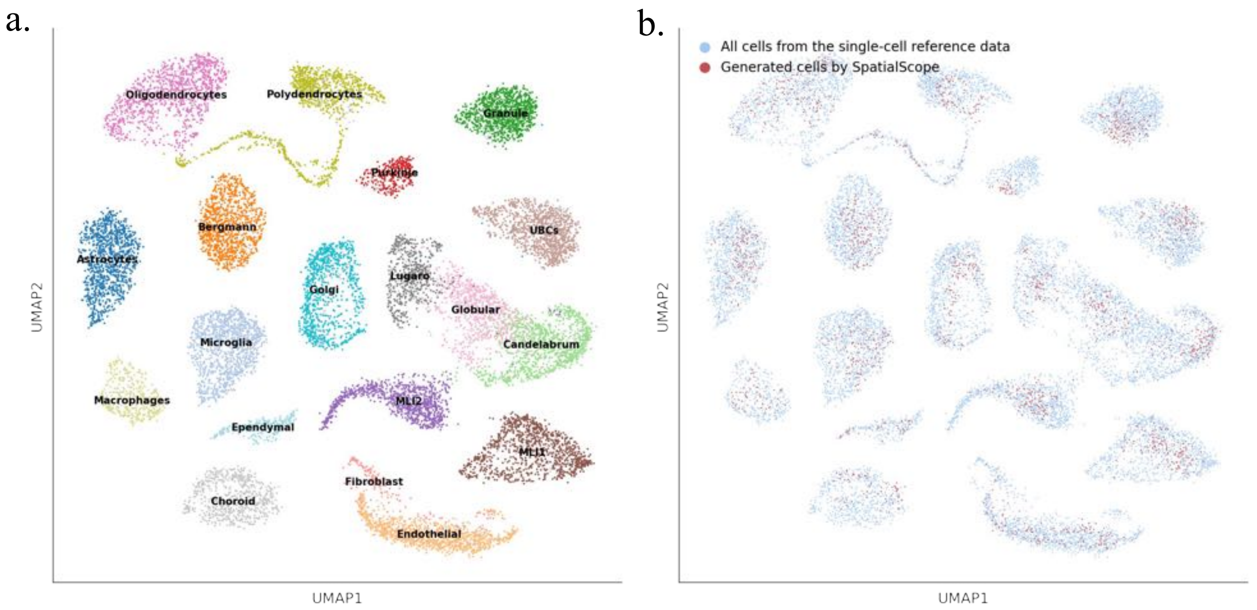

Figure S28: snRNA-seq reference of mouse cerebellum a, UMAP plot of snRNA-seq reference data with cell type annotations. **b**, UMAP plot of true cells and cells sampled from the learned distribution.

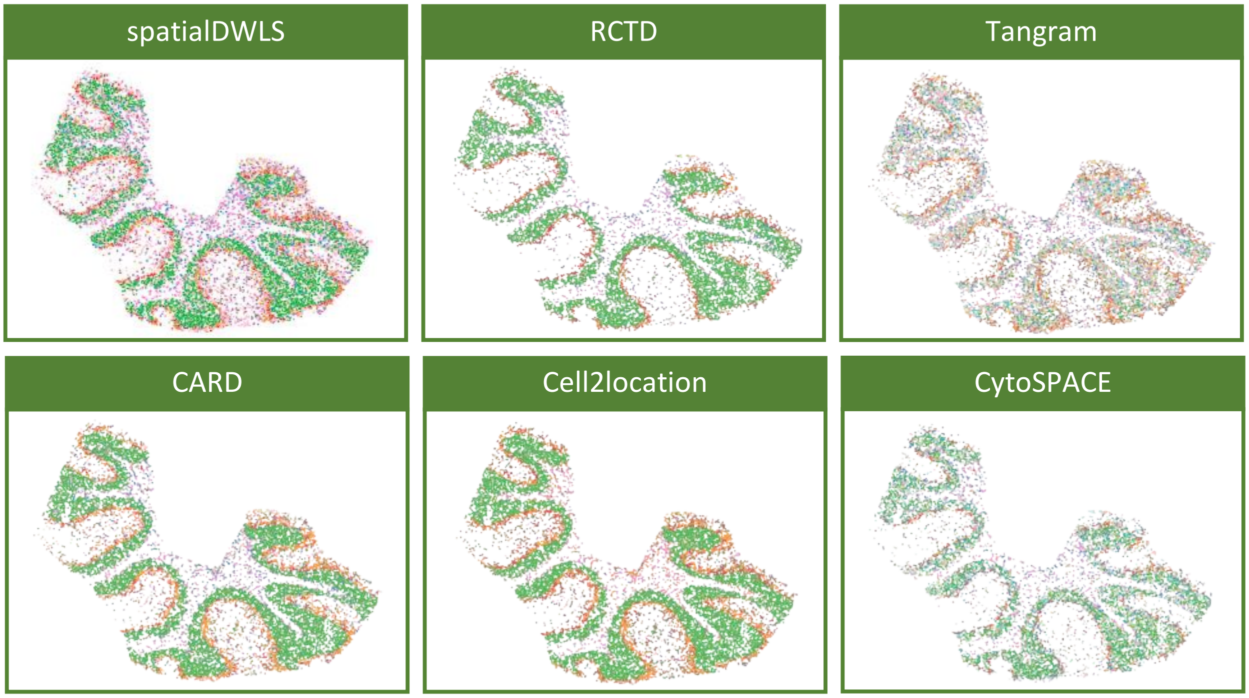

Figure S29: Cell type identification results by the compared methods for Slide-seq V2 mouse cerebellum data.

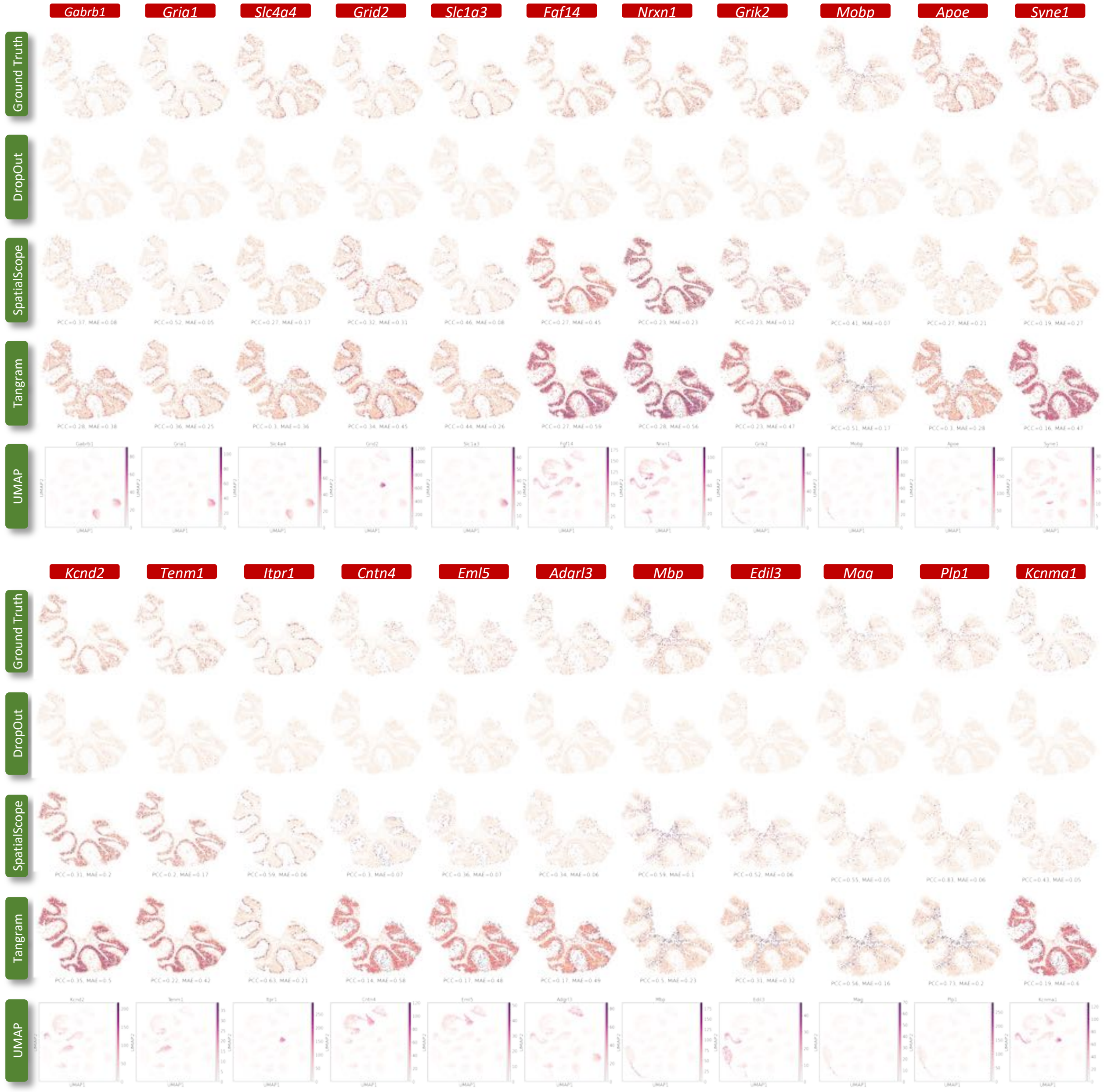

Figure S30: Simulated dropouts and correction results by SpatialScope and Tangram based on the Slide-seq data.

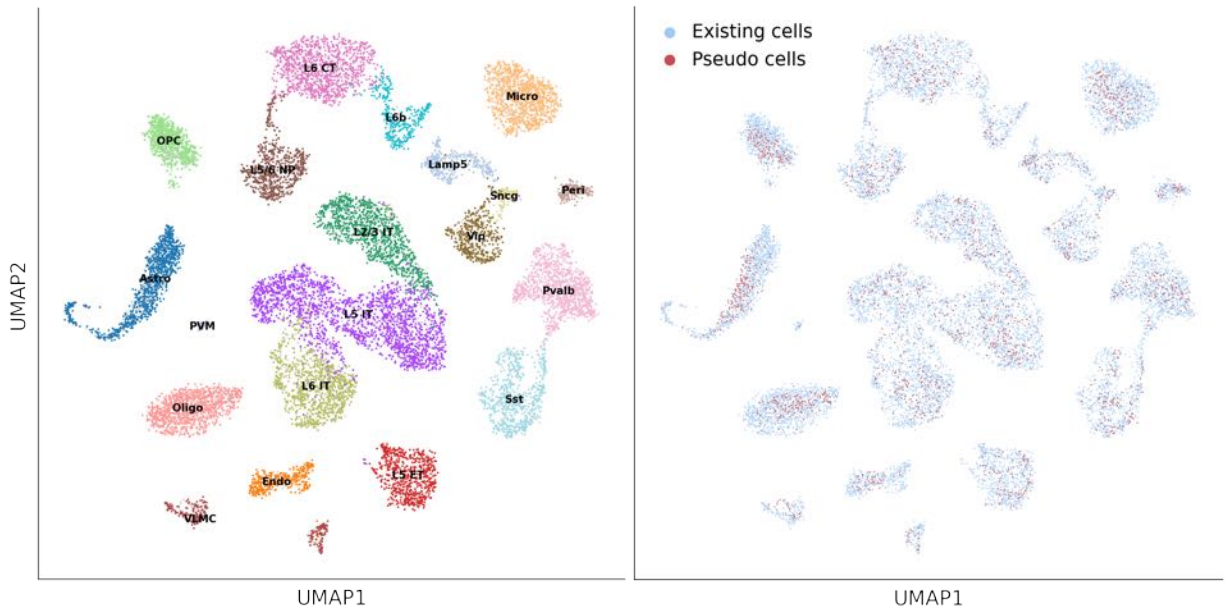

Figure S31: scRNA-seq reference of MERFISH data. UMAP plots of snRNA-seq reference data (left). UMAP of single cell reference data and the pseudo cells generated by deep generative model (right).

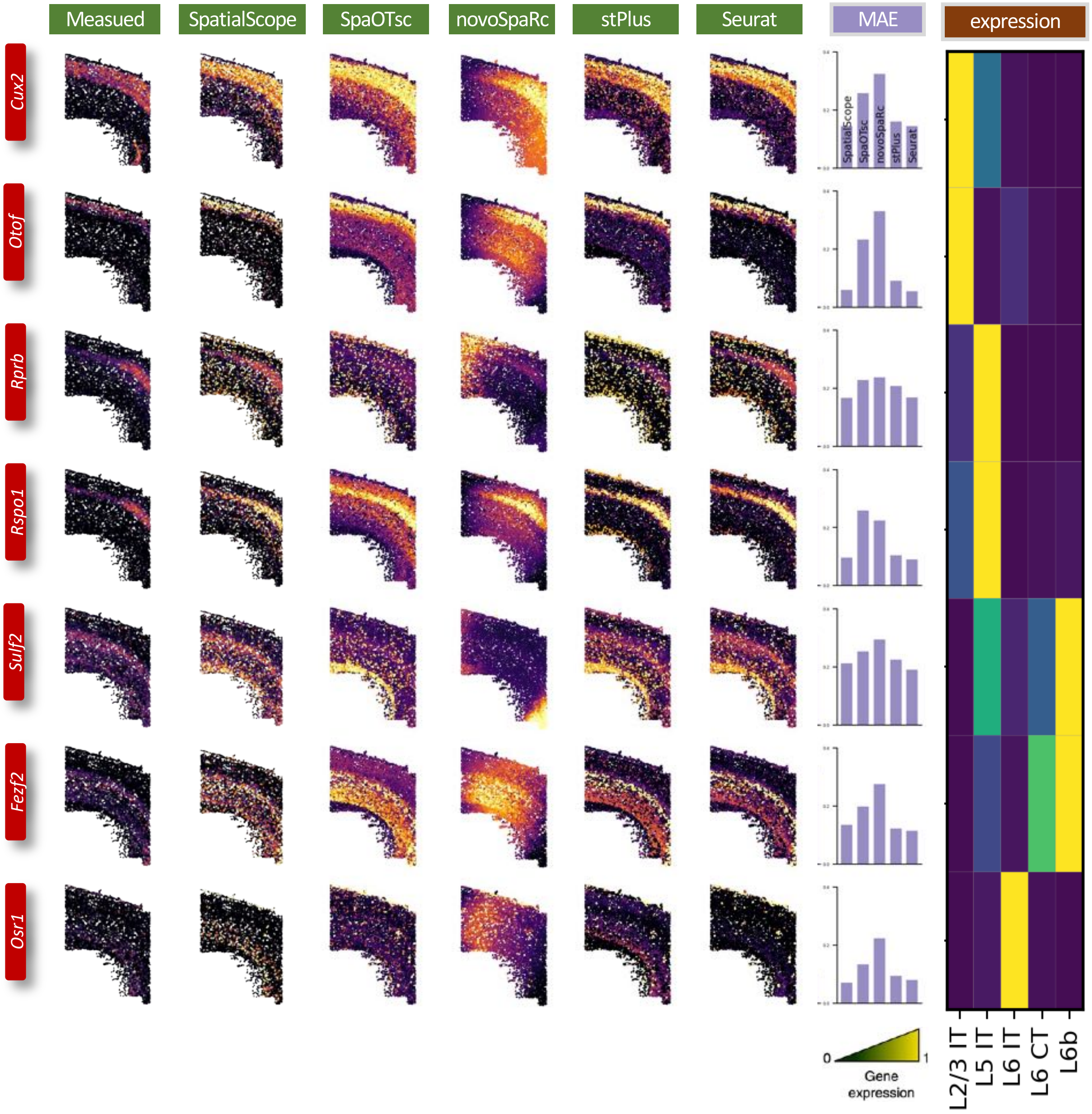

Figure S32: Comparison of gene expression imputation of more included methods for MERFISH genes. Measured and imputed expressions of known spatially patterned genes in the MERFISH dataset. Each row corresponds to a single gene. The first column from the left shows the measured spatial gene expression in the MERFISH dataset, while the second to sixth columns show the corresponding imputed expression pattern by SpatialScope, SpaOTsc, novoSpaRc, stPlus, and Seurat. The imputation accuracy was evaluated by MAE and displayed with bar plots (seventh column). The marker gene expression signatures in snRNA-seq reference were displayed with heatmap plots (eighth column).

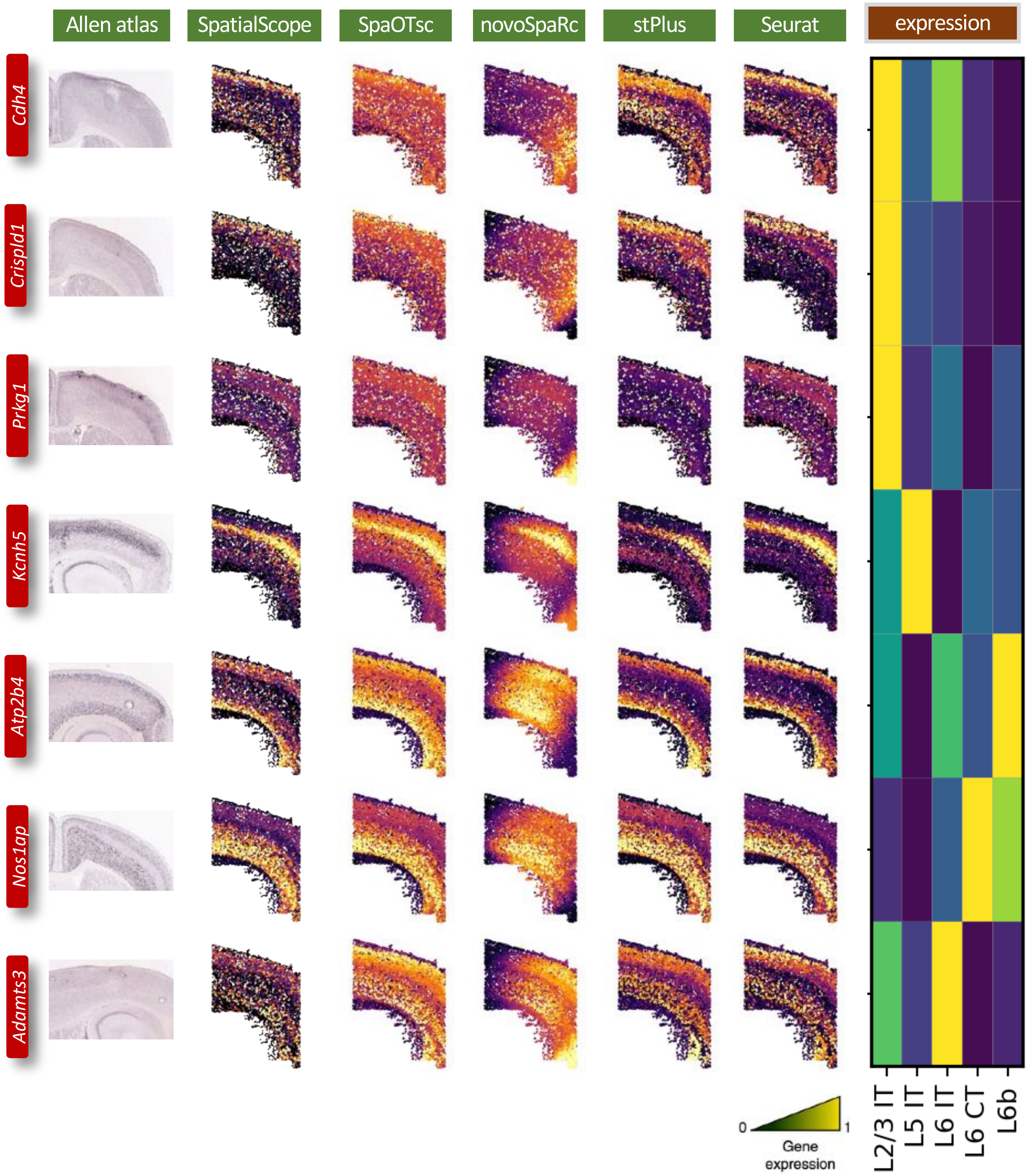

Figure S33: Comparison of gene expression imputation of more included methods for Non-MERFISH genes. Each row corresponds to a single gene. The first column from the left shows the ISH images from the Allen Brain Atlas, while the second to sixth columns show the corresponding imputed expression pattern by SpatialScope, SpaOTsc, novoSpaRc, stPlus, and Seurat. The imputation accuracy was evaluated by MAE and displayed with bar plots (seventh column). The marker gene expression signatures in snRNA-seq reference were displayed with heatmap plots (eighth column).

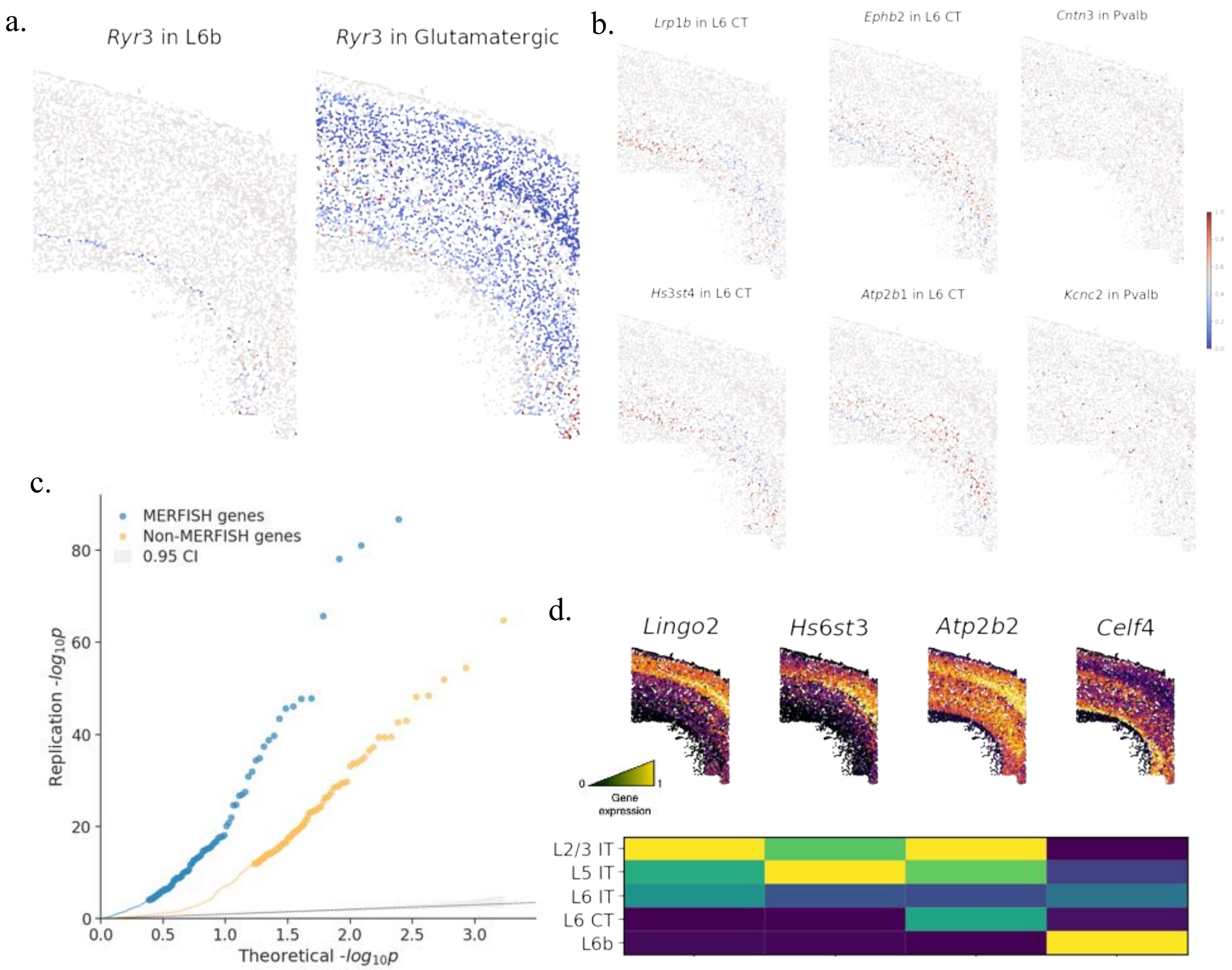

Figure S34: Spatially DE genes detection results on MERFISH data. **a**, The expression profile of *Ryr3* in L6b and Glutamatergic cell types, respectively. **b**, Representative examples of significant cell-type specific Non-MERFISH DE genes. **c**, QQ-plot of p-values for MERFISH and Non-MERFISH genes in the detection of spatially DE genes with SPARK-X. p values were calculated under the null condition in the permuted data with the two-sided test. **d**, Visualization of a few representative Non-MERFISH spatially DE genes detected by SPARK-X, the gene expression signatures in snRNA-seq reference were displayed with heatmap plot. Source data are provided as a Source Data file.

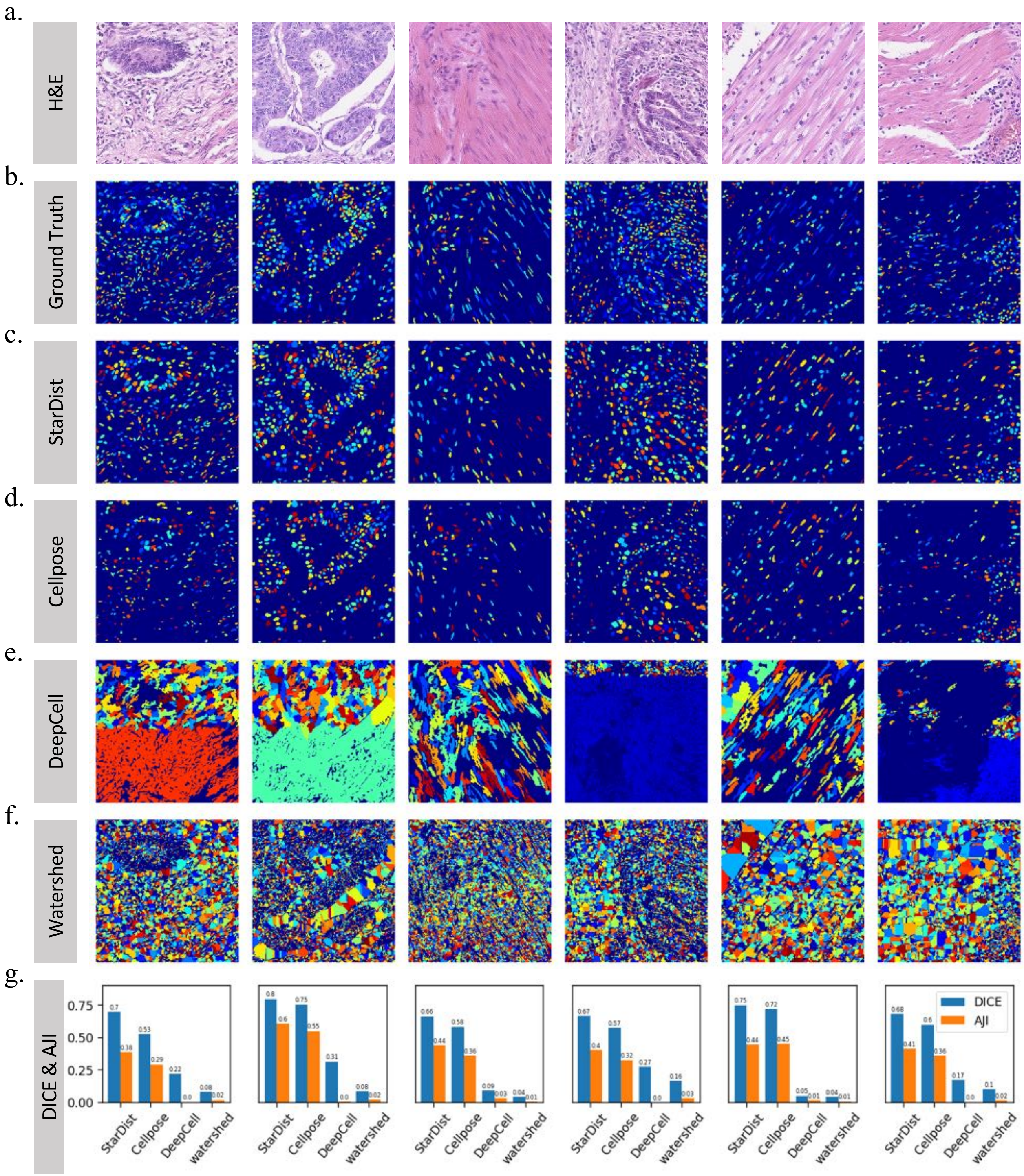

Figure S35: Comparison of nucleus segmentation performance among compared methods in CoNSep benchmarking dataset. **a**, H&E-stained histological images from CoNSep dataset. **b**, Manually annotated ground truth nuclei. **c**, Nucleus segmentation results by StarDist. **d**, Nucleus segmentation results by Cellpose. **e**, Nucleus segmentation results by DeepCell. **f**, Nucleus segmentation results by Watershed. **g**, DICE and AJI metrics.

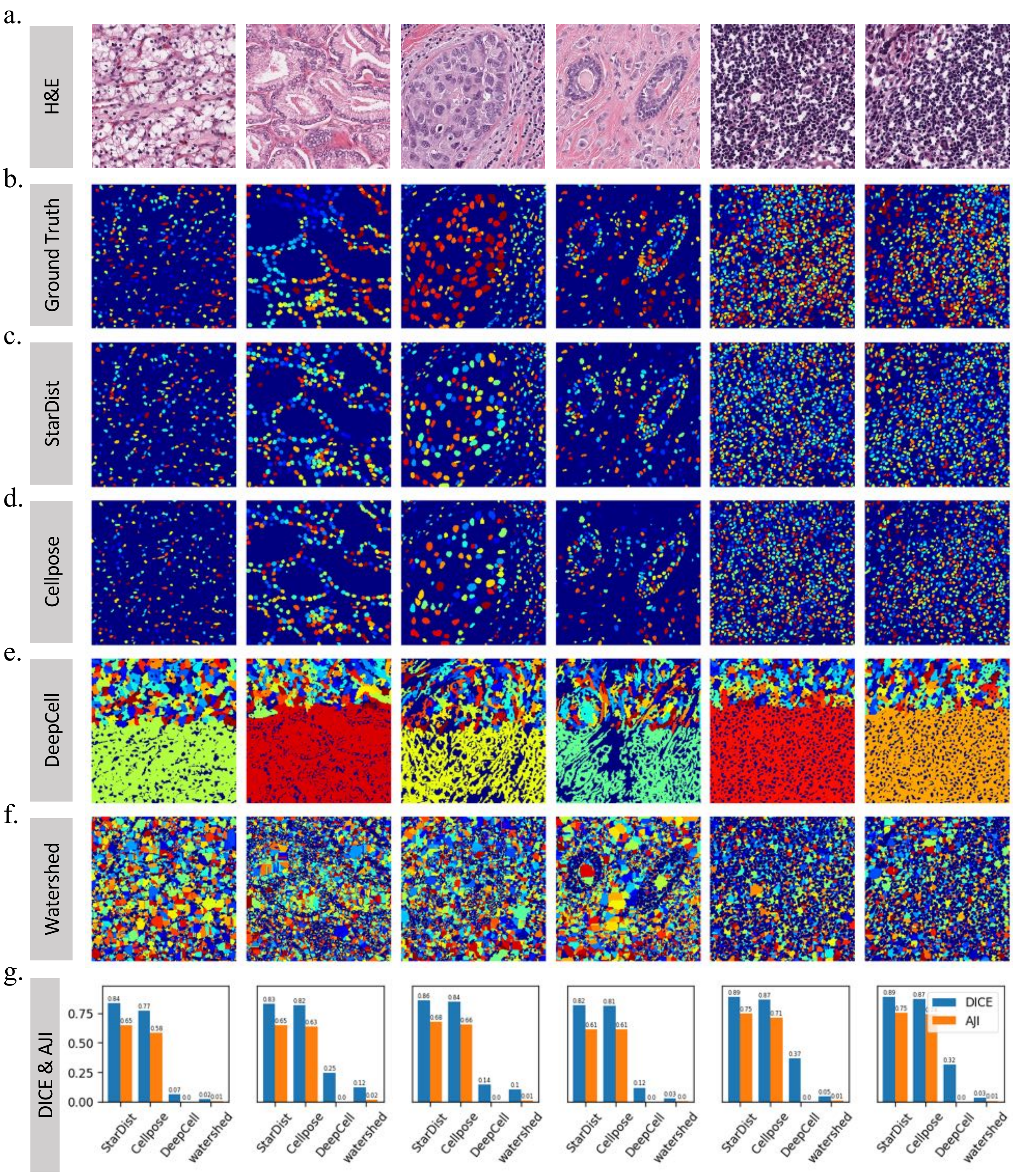

Figure S36: Comparison of nucleus segmentation performance among compared methods in Kumar benchmarking dataset. **a**, H&E-stained histological images from Kumar dataset. **b**, Manually annotated ground truth nuclei. **c**, Nucleus segmentation results by StarDist. **d**, Nucleus segmentation results by Cellpose. **e**, Nucleus segmentation results by DeepCell. **f**, Nucleus segmentation results by Watershed. **g**, DICE and AJI metrics.

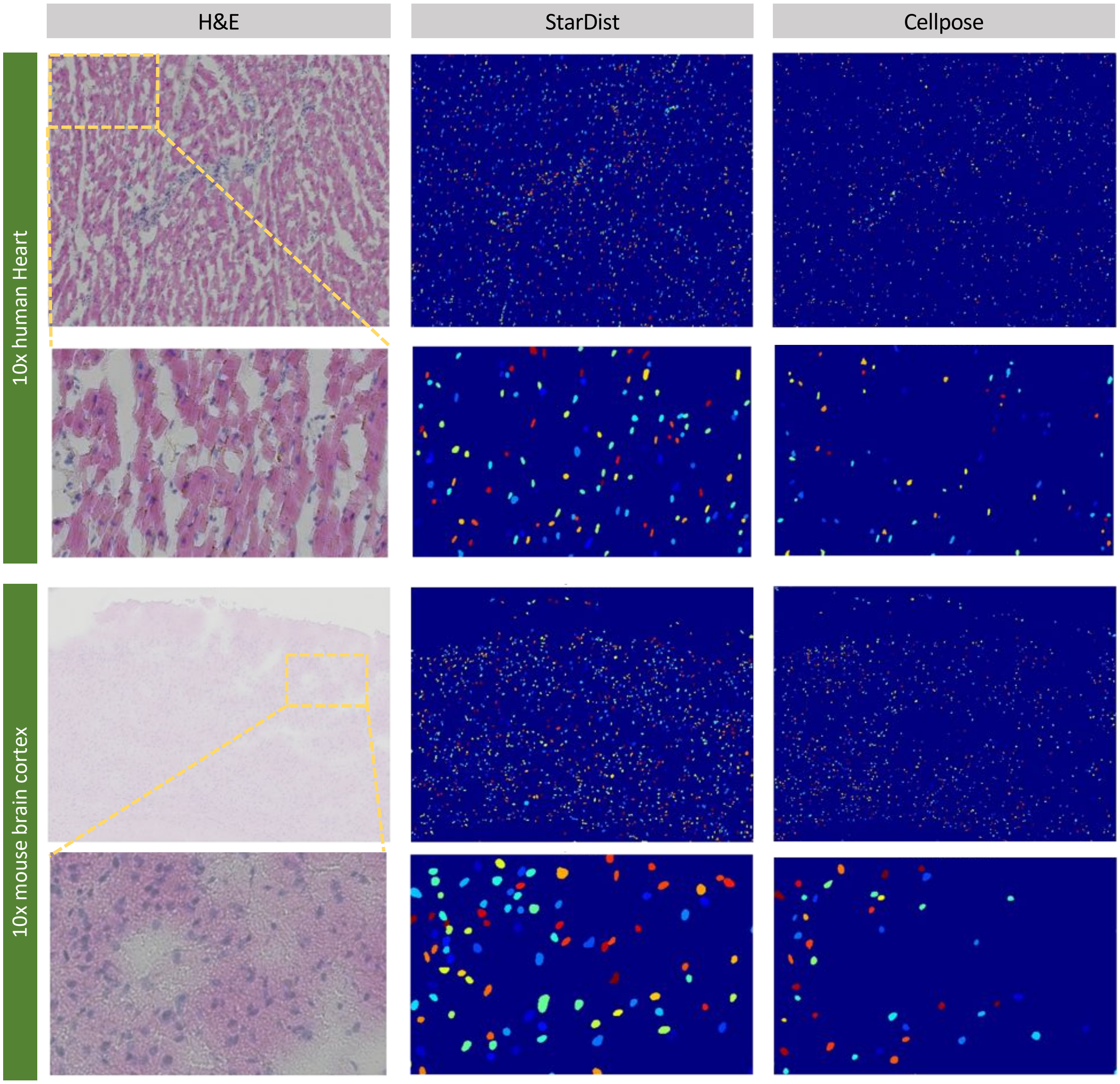

Figure S37: Comparison of nucleus segmentation performance between StarDist and Cellpose in two 10x Visium datasets. We applied Squidpy, which provides the interface of StarDist and Cellpose, to segment nuclei in the pair HE images. We used the default parameters following the instruction (`https://squidpy.readthedocs.io/en/stable/index.html`). In the first 10x human heart data, the H&E-stained histological image (first column) was used as input. The segmentation results of StarDist and Cellpose were shown in the second and last column, respectively, where StarDist located 1797 single cells and Cellpose only found 1301 cells. Clearly, Cellpose performed worse as a result of substantial missing cells, especially in the zoom-in region. For the second 10x mouse brain cortex dataset, we observed similar results that StarDist (n=1563) segments more cells than Cellpose (n=1250).

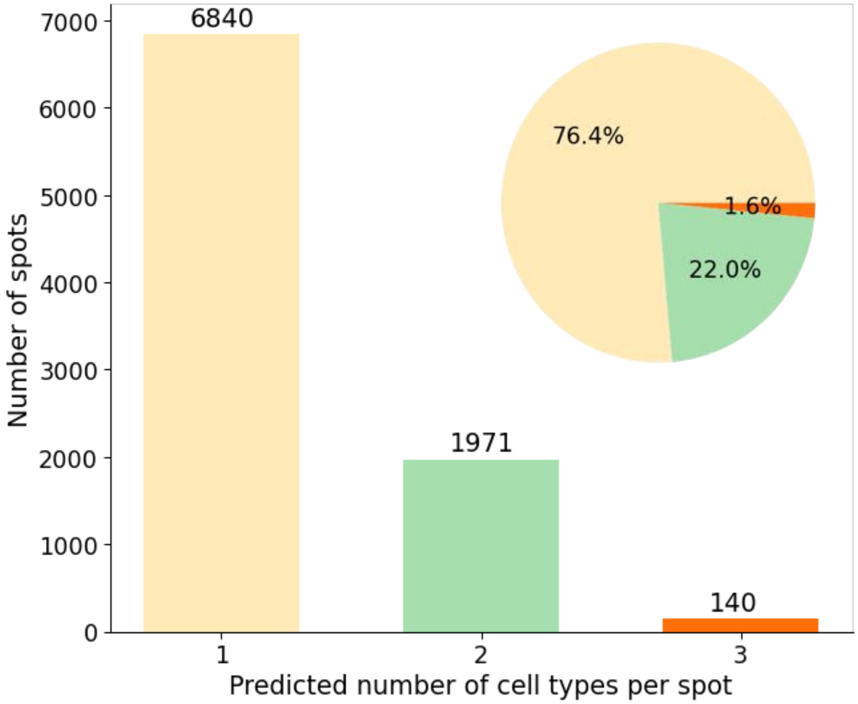

Figure S38: Number of cell types per Slide-seq V2 cerebellum spot. In total, 22.0% and 1.6% of spots were predicted to contain two and three cell types, respectively.

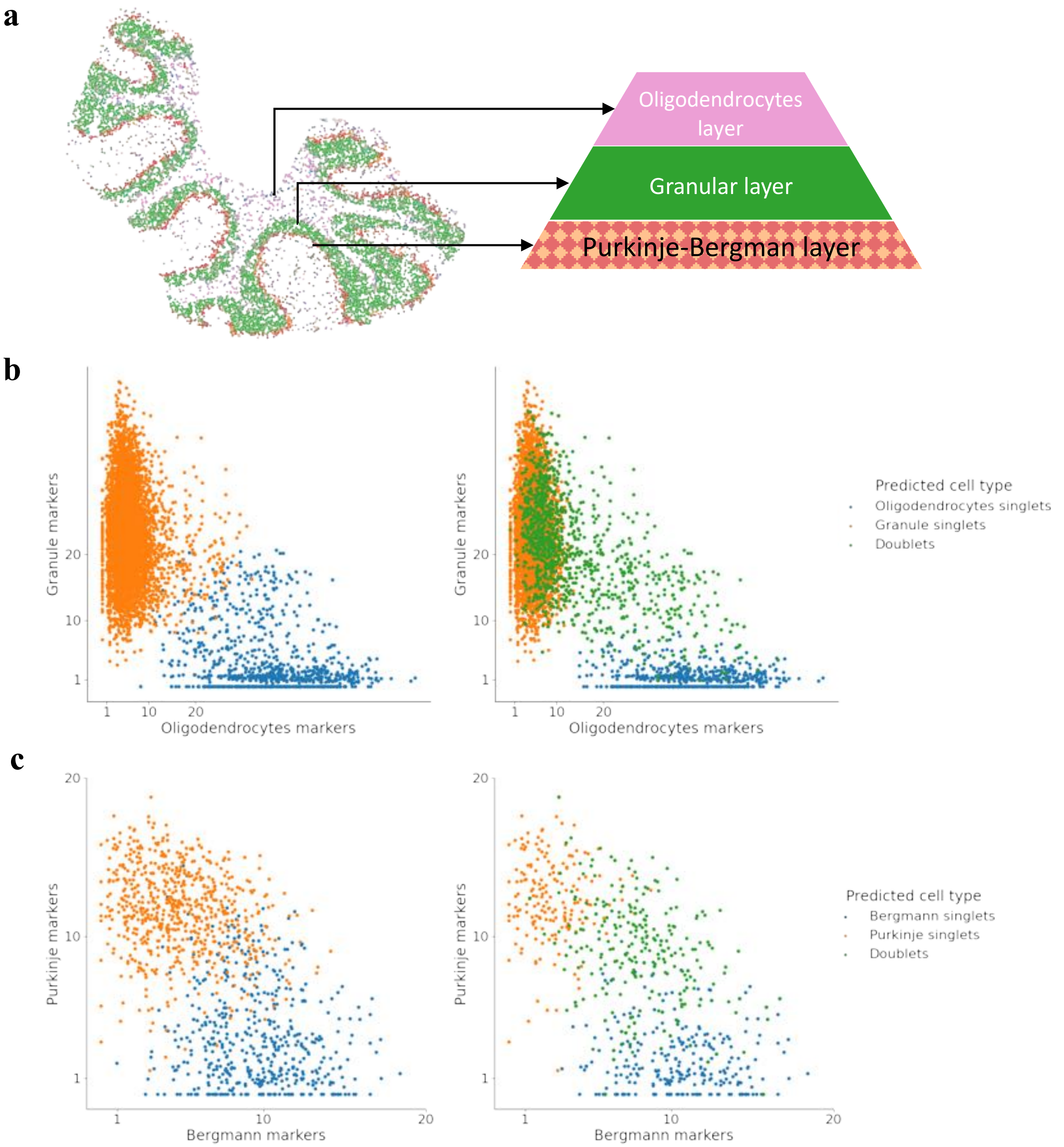

Figure S39: Benefits of at most two cell co-exist assumption for Slide-seq dataset. **a**, Cell type identification results by SpatialScope correctly captured the layered architecture (Oligodendrocytes layer, Granular layer and Purkinje-Bergman layer) of cerebellum. **b**, Expression of Granule and Oligodendrocytes cell marker genes for spots with Granule, Oligodendrocytes or doublet (mixture of Granule and Oligodendrocytes) cell type assignment when assuming at most one cell (left panel) or two-cell (right panel) co-exist within a spot. **c**, Expression of Bergmann and Purkinje cell marker genes for spots with Bergmann, Purkinje or doublet (mixture of Bergmann and Purkinje) cell type assignment when assuming at most one cell (left panel) or two-cell (right panel) co-exist within a spot. Notably, Bergmann and Purkinje cells spatially colocalize to the same layer, resulting in a population of spots exhibiting marker gene expression signatures from both cell types. This observation strongly suggests that these spots contain fractional representations of both Purkinje and Bergman cells. If we simply assume that there is only one cell in a spot, then these doublet spots will be incorrectly assigned with one cell type only (left panel). In contrast, the flexible assumption adopted by our method can automatically distinguish doublets from singlets (right panel). Consequently, we are able to accurately assign the mixed cell types for these doublet spots, thus yielding a more elucidated and comprehensive depiction of tissue structures. Source data are provided as a Source Data file.

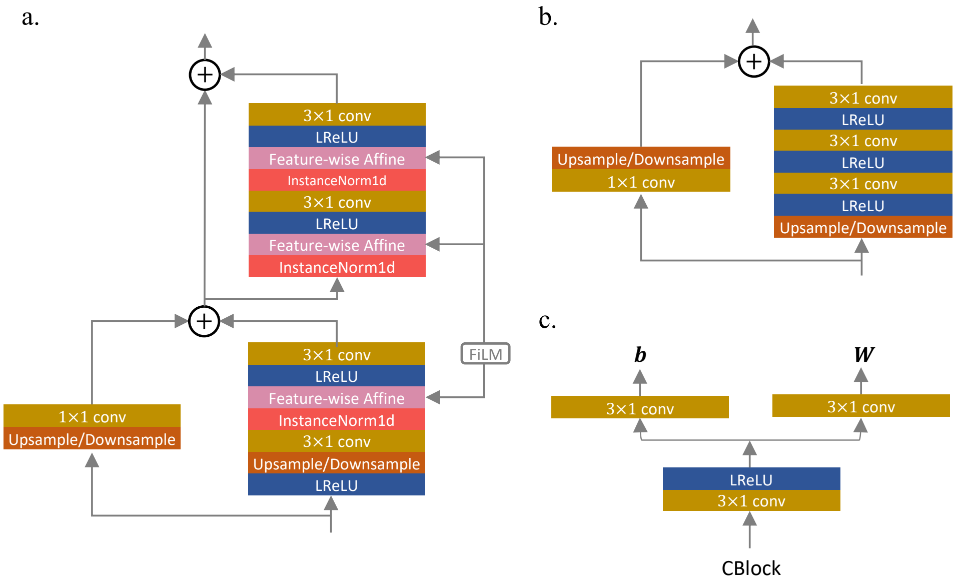

Figure S40: The diagrams of MBlock, CBlock and FiLM. **a**, The diagram of MBlock. Two residual blocks are used. The output of the FiLM block will be the input to the Feature-wise Affine block, as shown in Equation (26). **b**, The diagram of CBlock. One residual block is used, and this block's output will be the FiLM block's input (Fig. S40c). **c**, The diagram of FiLM. The block will take the output of CBlock as input and output scale **W** and shift **b**.

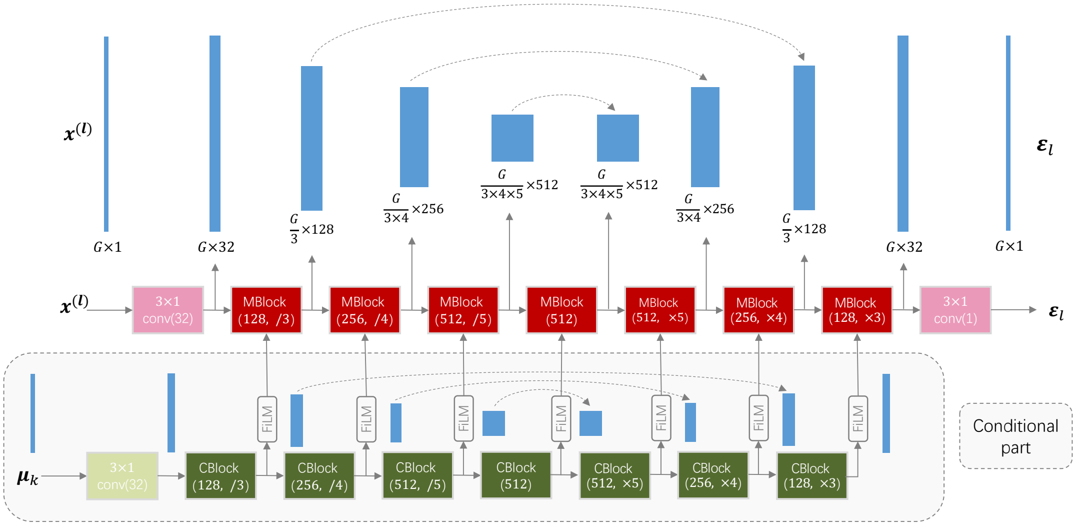

Figure S41: Architecture of the conditional *score network* $\mathbf{s}_{\boldsymbol{\theta}}(\mathbf{x}, C)$. The red color line is the UNet that takes $\mathbf{x}$ as input, and the green one is the conditional UNet that takes $\boldsymbol{\mu}_k$ as input, where k in $\boldsymbol{\mu}_k$ is the cell type that $\mathbf{x}$ belongs to. We condition cell type information into score function by FiLM module, which produces both scale and bias vectors for feature-wise affine transformation as shown in Equation (26).

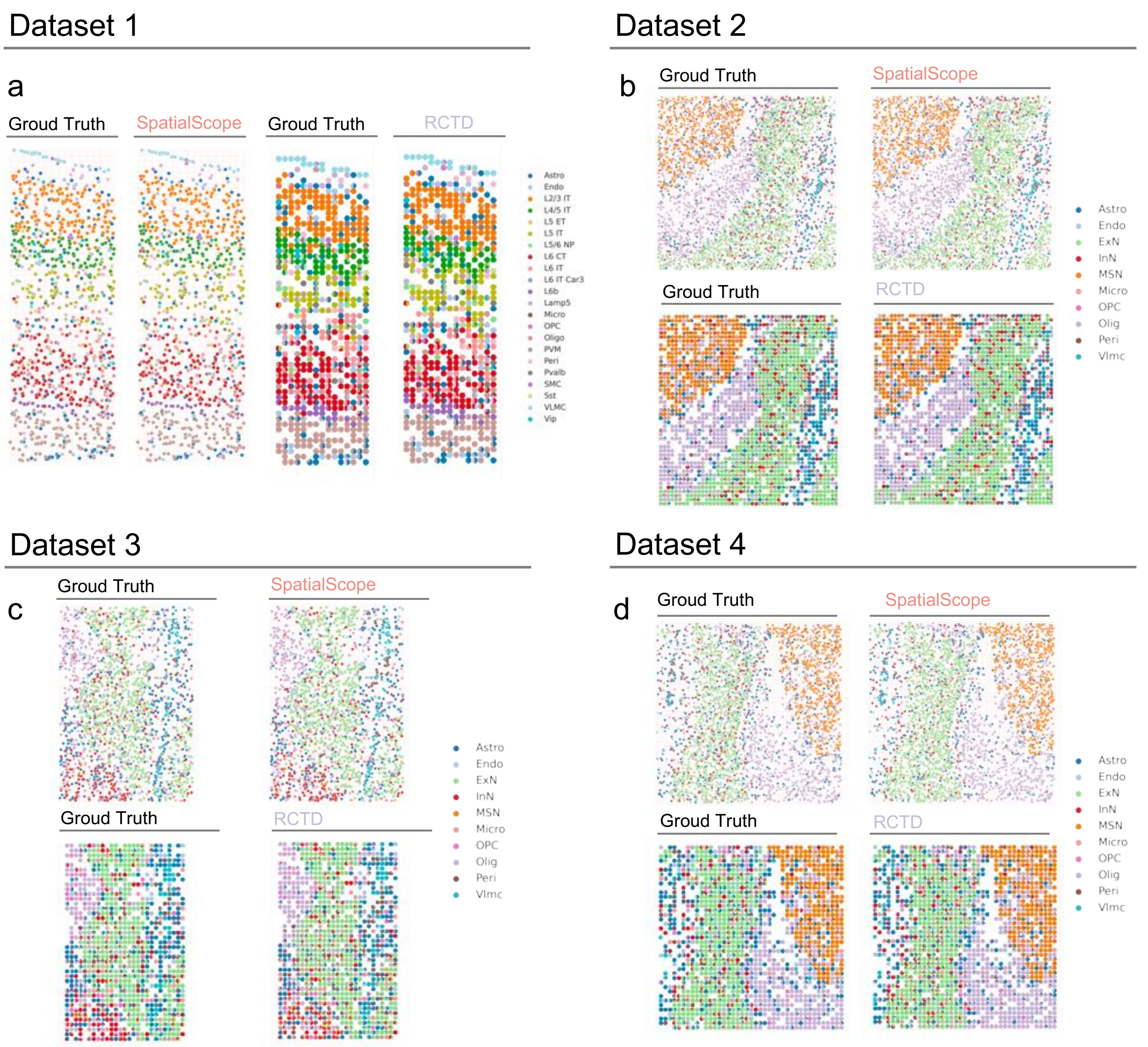

Figure S42: Visualization of the results of "Cell type identification" given by SpatialScope and RCTD for Datasets 1, 2, 3, and 4. (**a** left, **b,c,d** upper) Spatials scatter plots display the ground truth and the result of Cell type identification given by SpatialScope of cell types at single-cell resolution. (**a** right, **b,c,d** lower) Spatial scatter pie plots display the ground truth and the inferred cell-type compositions by RCTD at the spot level.

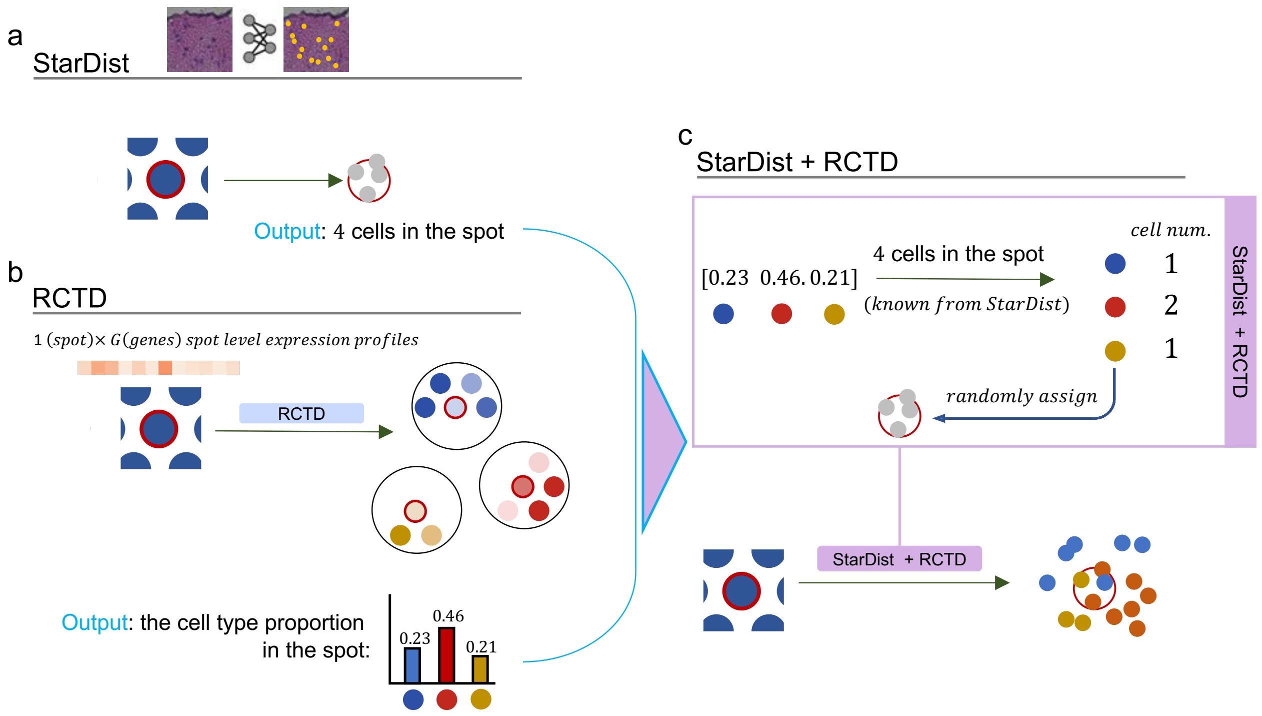

Figure S43: The workflow of StarDist+RCTD, a generalization of RCTD to make SpatialScope's "Cell type identification" step comparable with RCTD a, StarDist+RCTD borrow "Nucleus segmentation" step of SpatialScope to detect cell number in each spot. Upper, StarDist uses networks to complete cell segmentation of H&E image. Lower, Blue circle on the left represents spots in spatial data, and the red circle indicates the spot we focus on. "Nucleus segmentation" outputs the cell number in the spots. In the example of a red circled spot, there are 4 cells. **b**, RCTD outputs continuous cell-type proportions of spots. Opacity represents the inferred cell type proportion (right), and different color represents different cell types. In a red circled spot example, RCTD outputs cell type proportion $(0.23, 0.46, 0.21)$ for blue, red, and yellow cell types, respectively. **c**, Upper, StarDist+RCTD discretizes the cell type proportion produced by RCTD to get the distribution of single-cell cell type labels then randomly assign them to cell locations. In the example of a red circled spot, StarDist+RCTD outputs 1 blue cell, 2 red cells, and 1 yellow cell in the spot after discretization. Lower, the generalization version of RCTD, StarDist+RCTD, can output a single-cell resolution spatial landscape of cell types to make RCTD comparable with SpatialScope's "Cell type identification" step.

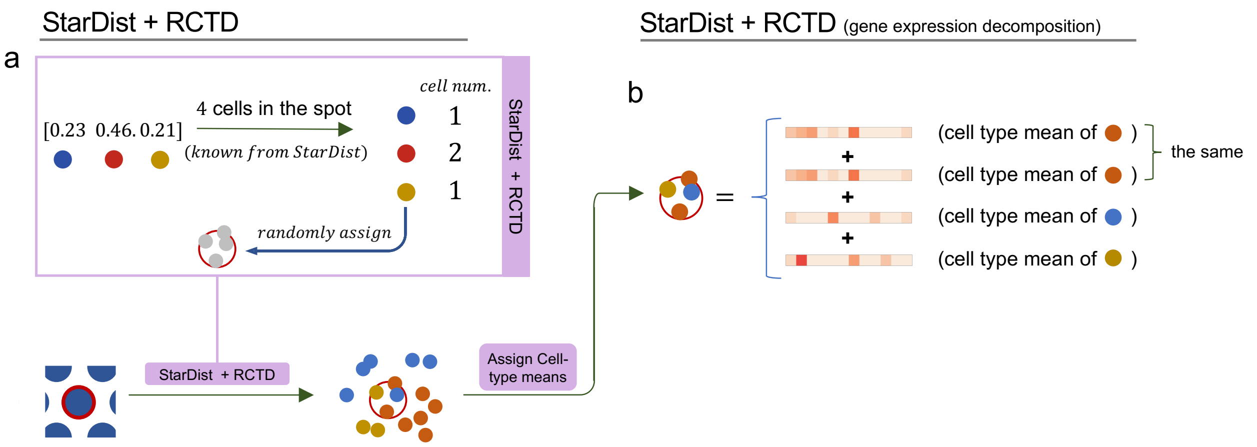

Figure S44: The workflow of StarDist+RCTD and use the average expression of cell types for gene expression decomposition. **a**, Upper, StarDist+RCTD discretize the cell type proportion produced by RCTD to get the distribution of single-cell cell type labels then randomly assign them to cell locations. In the example of a red circled spot, StarDist+RCTD outputs 1 blue cell, 2 red cells, and 1 yellow cell in the spot after discretization. Lower, the generalization version of RCTD, StarDist+RCTD, can output a single-cell resolution spatial landscape of cell types to make RCTD comparable with SpatialScope's "Cell type identification" step. **b**, By assigning the average expression of cell types to each cell location according to their identified cell types in **a**, StarDist+RCTD achieves gene expression decomposition. For example, the upper and lower cell is identified as red cells by StarDist+RCTD; their decomposed single-cell expression is cell type means of red cell type. The left/right cell is identified as yellow/blue cells by StarDist+RCTD; their decomposed single-cell expression is cell type means of yellow/blue cell type.

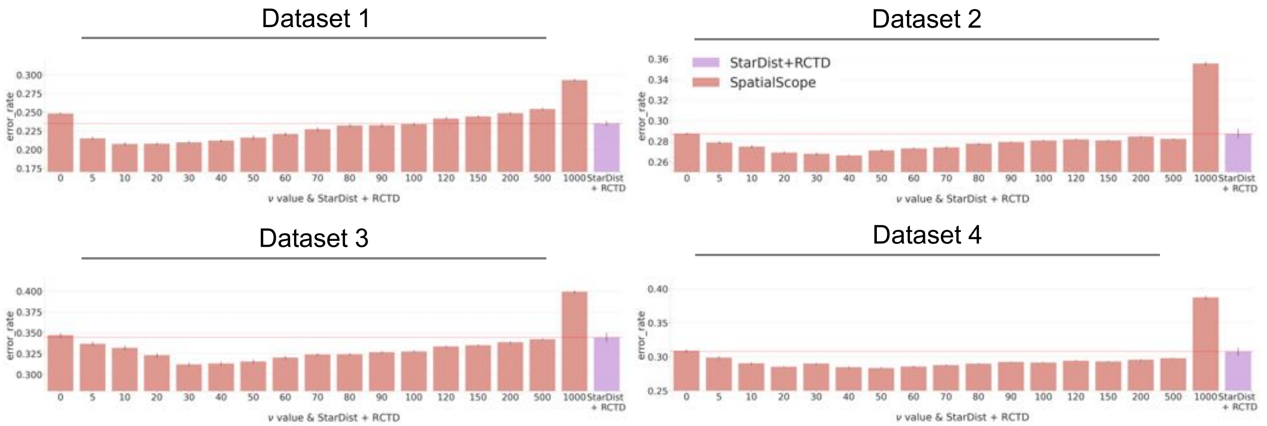

Figure S45: The effect of incorporating spatial information under different ν values in cell type identification task compared with the "StarDist+RCTD" method for Datasets 1, 2, 3, and 4. Bar plots of the error rates under different ν values for the cell type identification step of SpatialScope and the "StarDist+RCTD" method. The red line indicates the error rate of the "StarDist+RCTD" method as a baseline. Data are presented as mean values ±95% confidence intervals; $n = 10$ simulation replicates. Source data are provided as a Source Data file.

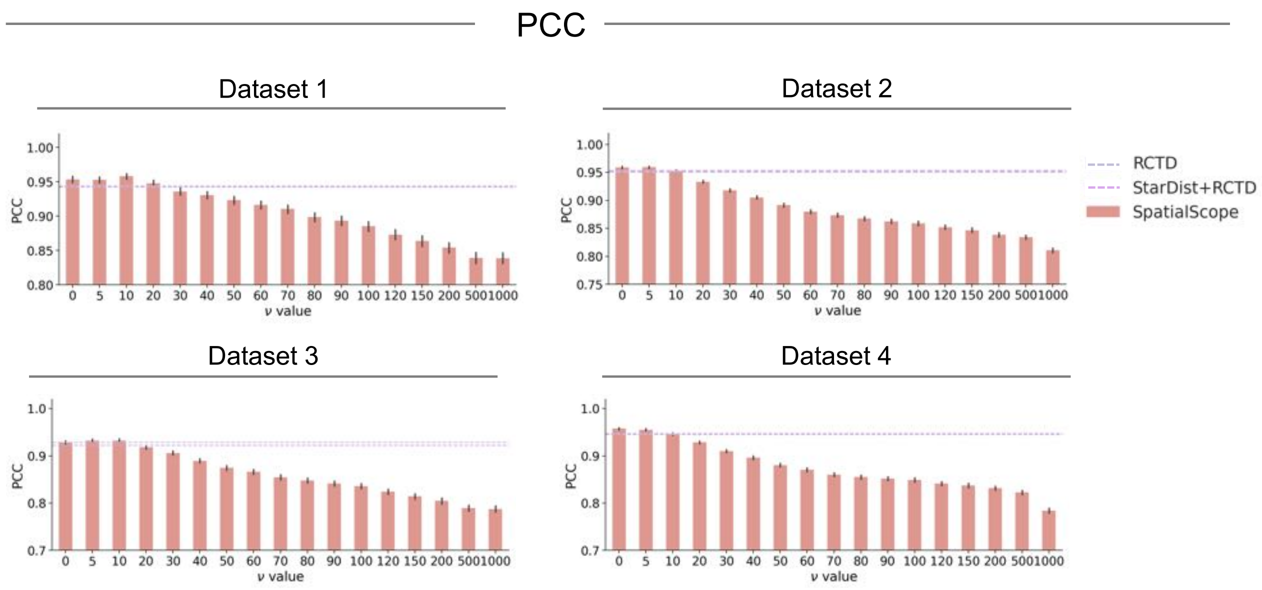

Figure S46: The effect of incorporating spatial information under different ν in deconvolution task compared with baseline method RCTD and StarDist+RCTD for Datasets 1, 2, 3, and 4. Metric: PCC, the higher the better. Bar plots of PCC under different ν values for the cell type identification step of SpatialScope are shown. The blue and purple lines represent the PCC values of RCTD and StarDist+RCTD, respectively, serving as baselines. Data are presented as mean values ±95% confidence intervals; $n = 10$ simulation replicates. Source data are provided as a Source Data file.

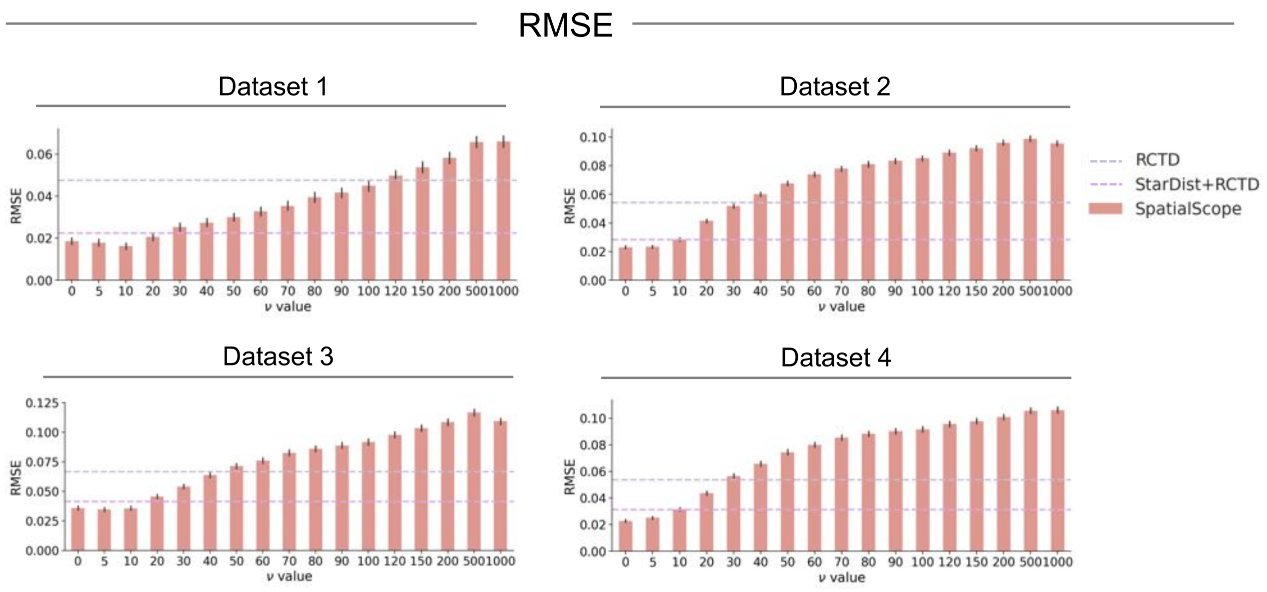

Figure S47: The effect of incorporating spatial information under different ν in deconvolution task compared with baseline method RCTD and StarDist+RCTD for Datasets 1, 2, 3, and 4. Metric: RMSE, the lower the better. Bar plots of the error rate under different ν values for the cell type identification step of SpatialScope are displayed. The blue and purple lines represent the RMSE values of RCTD and StarDist+RCTD, respectively, serving as baselines. Data are presented as mean values ±95% confidence intervals; $n = 10$ simulation replicates. Source data are provided as a Source Data file.

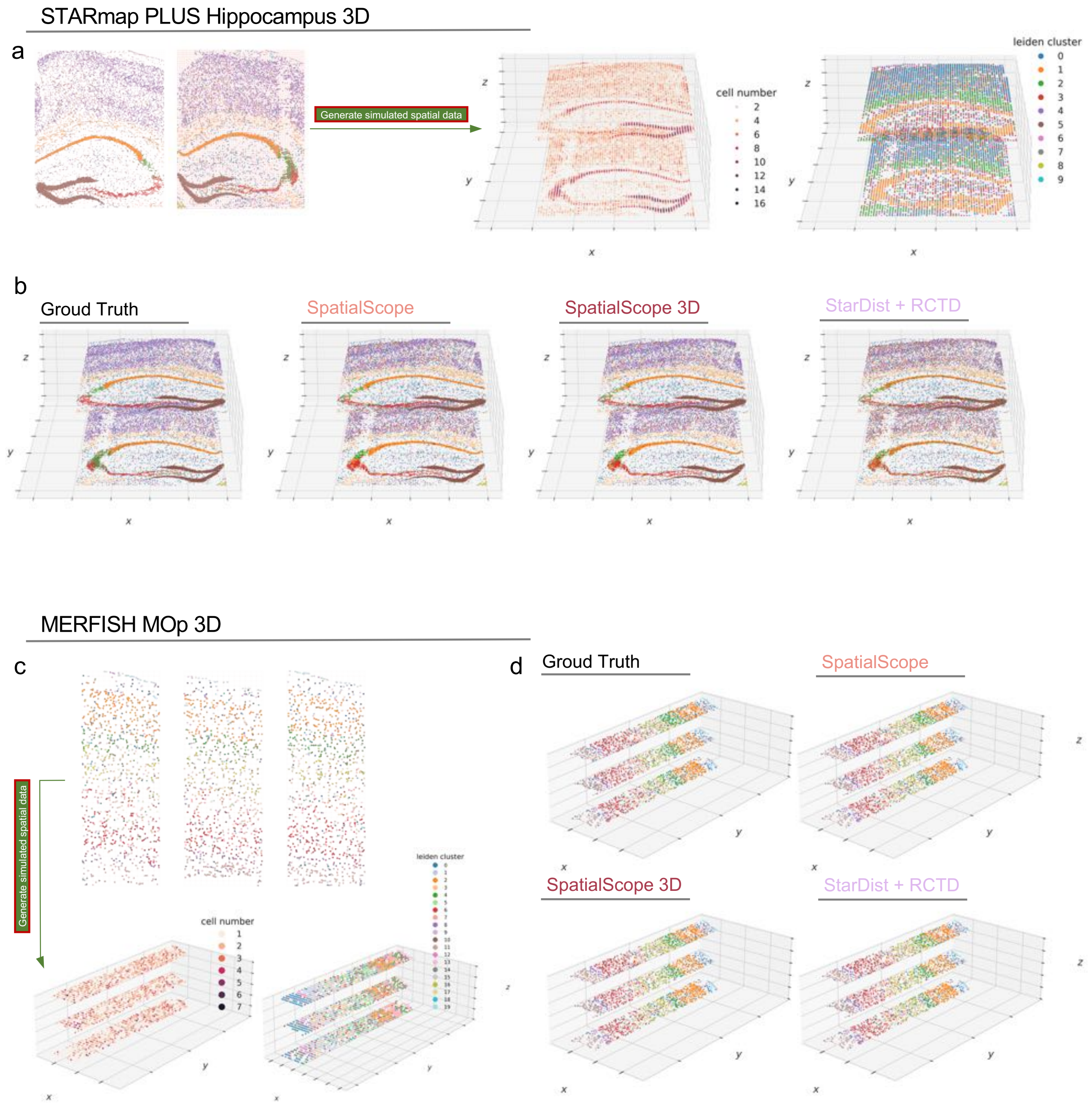

Figure S48: Visualization of SpatialScope "Cell type identification" and RCTD results at 2 simulated multi-slice data. **a,c**, 2 multi-slice data with annotated cell types for every single cell are used to generate simulated spatial data. **a** left and **c** upper, a spatial scatter plot displays single cell types on each cell location from the ground truth. Red dashed lines indicate the gridding for aggregating the cells to simulate spots. Different color represents different cell types. **a** right and **c** lower, scatter plot of simulated spots and the cell number of spots (represented by color) and Leiden clustering result. Different color represents different clusters. **b,d**, Results of SpatialScope's "Cell type identification" step assigning neighboring region only across single slices (named SpatialScope), SpatialScope's "Cell type identification" step assigning neighboring region across multiple slices (named SpatialScope 3D) and StarDist+RCTD. Figures show spatial scatter plot displaying identified single cell types on each cell location from ground truth and 3 methods.

STARmap PLUS Hippocampus 3D

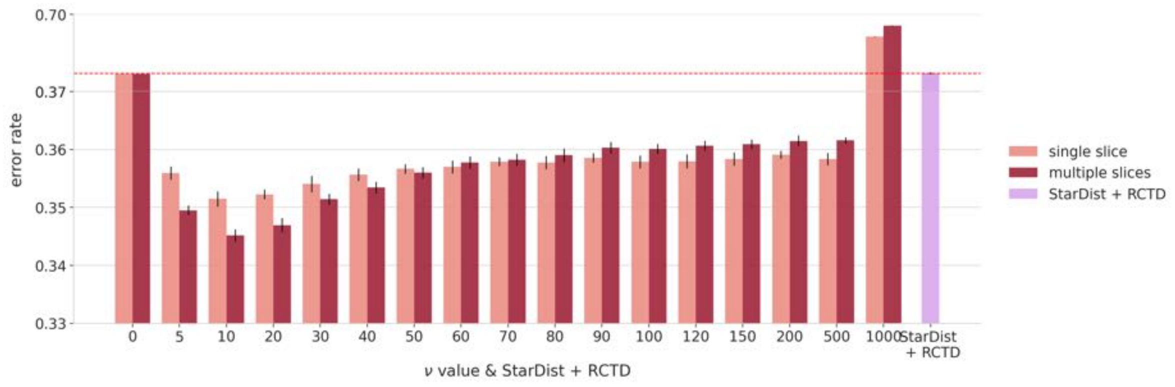

MERFISH MOp 3D

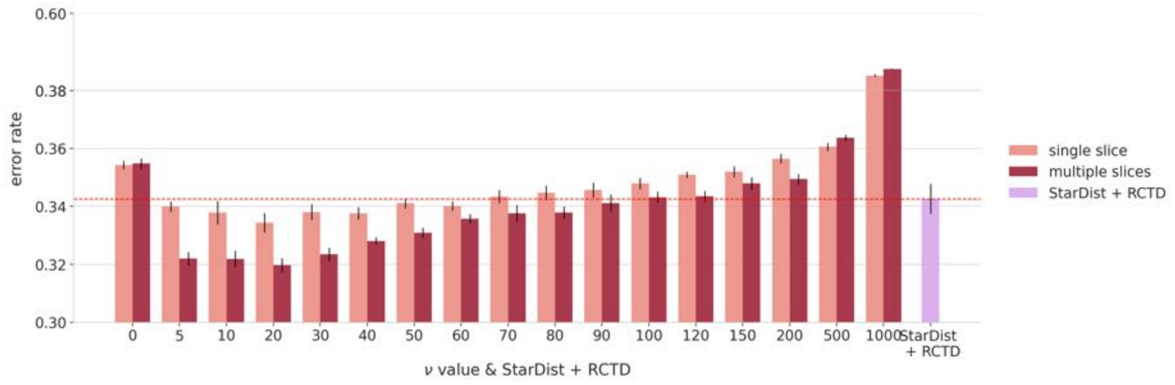

Figure S49: Comparason of performance of "Cell type identification" using multiple-slice spatial information, "Cell type identification" only using single-slice information and StarDist+RCTD in cell type identification task. Bar plots of error rate under different ν values of SpatialScope's "Cell type identification" step assigning neighboring region only across single slices (single slice), SpatialScope's "Cell type identification" step assigning neighboring region across multiple slices (multiple slices) and StarDist+RCTD. The red dashed line indicates the error rate of StarDist+RCTD as baselines. Data are presented as mean values with error bars representing one standard deviation from the mean. The error bars were computed based on $n = 10$ simulation replicates. Source data are provided as a Source Data file.

STARmap PLUS Hippocampus 3D

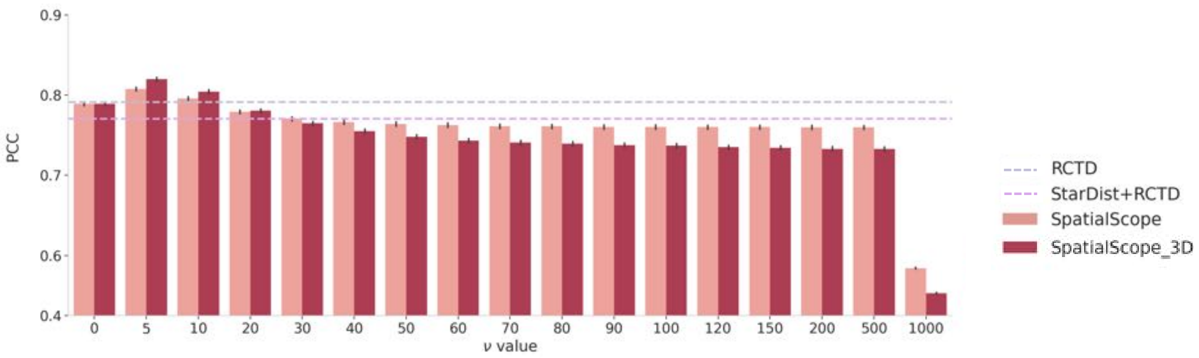

MERFISH MOp 3D

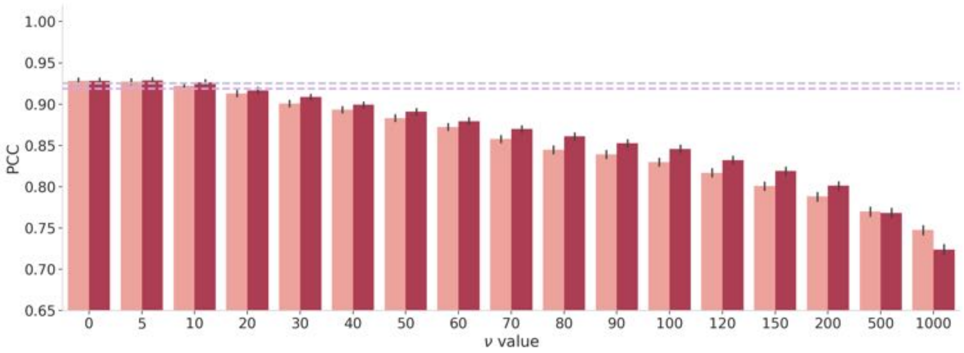

Figure S50: Comparason of performance of “Cell type identification” using multiple-slice spatial information, “Cell type identification” only using single-slice information, RCTD, and StarDist + RCTD in deconvolution task. Metric: PCC, the higher the better. Bar plots of PCC under different ν values of SpatialScope’s “Cell type identification” step assigning neighboring region only across single slices (single slice), SpatialScope’s “Cell type identification” step assigning neighboring region across multiple slices (multiple slices). The blue and purple line indicates the PCC of RCTD and StarDist + RCTD, respectively, as baselines. Data are presented as mean values ±95% confidence intervals; $n = 10$ simulation replicates. Source data are provided as a Source Data file.

STARmap PLUS Hippocampus 3D

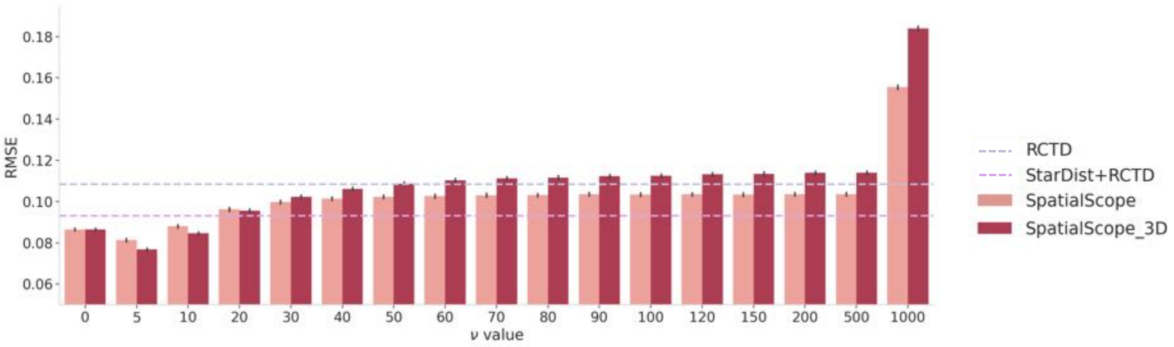

MERFISH MOp 3D

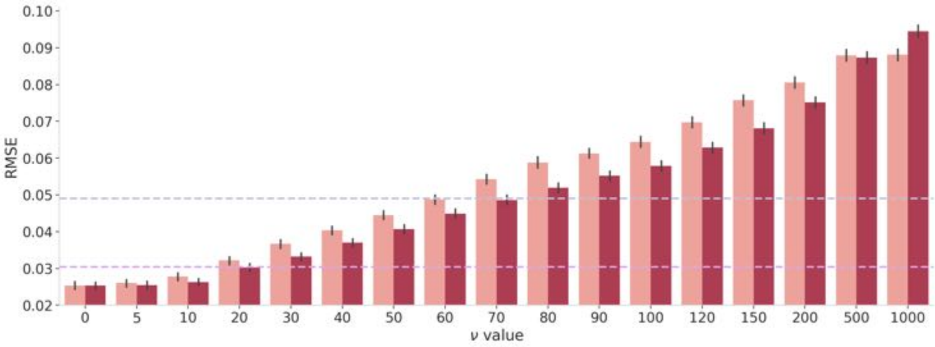

Figure S51: Comparason of performance of "Cell type identification" using multiple-slice spatial information, "Cell type identification" only using single-slice information, RCTD, and StarDist + RCTD in deconvolution task. Metric: RMSE, the lower the better. Bar plots of RMSE under different ν values of SpatialScope's "Cell type identification" step assigning neighboring region only across single slices (single slice), SpatialScope's "Cel l type identification" step assigning neighboring region across multiple slices (multiple slices). The blue and purple line indicates the RMSE of RCTD and StarDist + RCTD, respectively, as baselines. Data are presented as mean values ±95% confidence intervals; $n = 10$ simulation replicates. Source data are provided as a Source Data file.

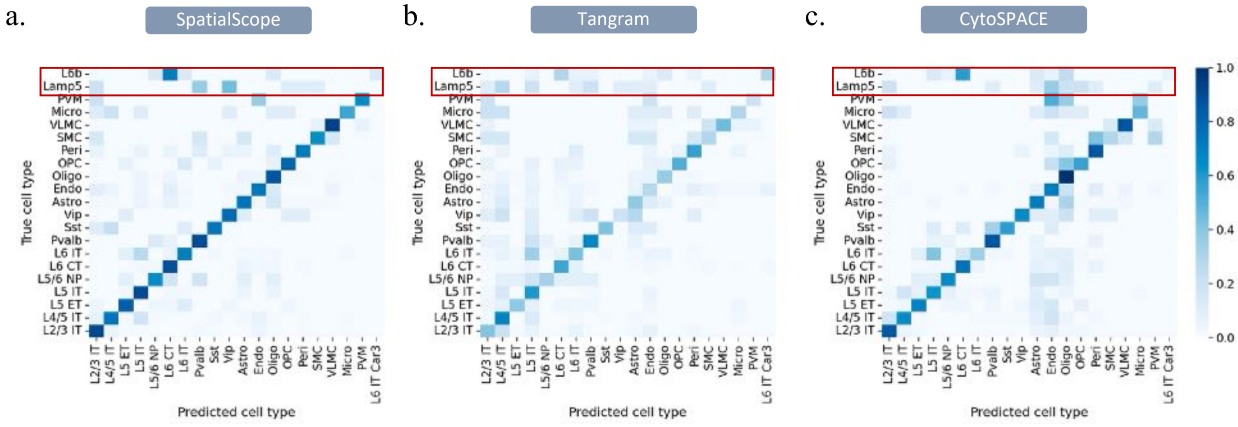

Figure S52: Performance of SpatialScope and the compared method on cell types that do not appear in the single-cell reference. **a**, Confusion matrix for SpatialScope's performance on missing cell types simulation analysis. Color represents the proportion of the cell type on the y-axis, classified as the cell type on the x-axis. **b**, Confusion matrix of Tangram. **c**, Confusion matrix of CytoSPACE.

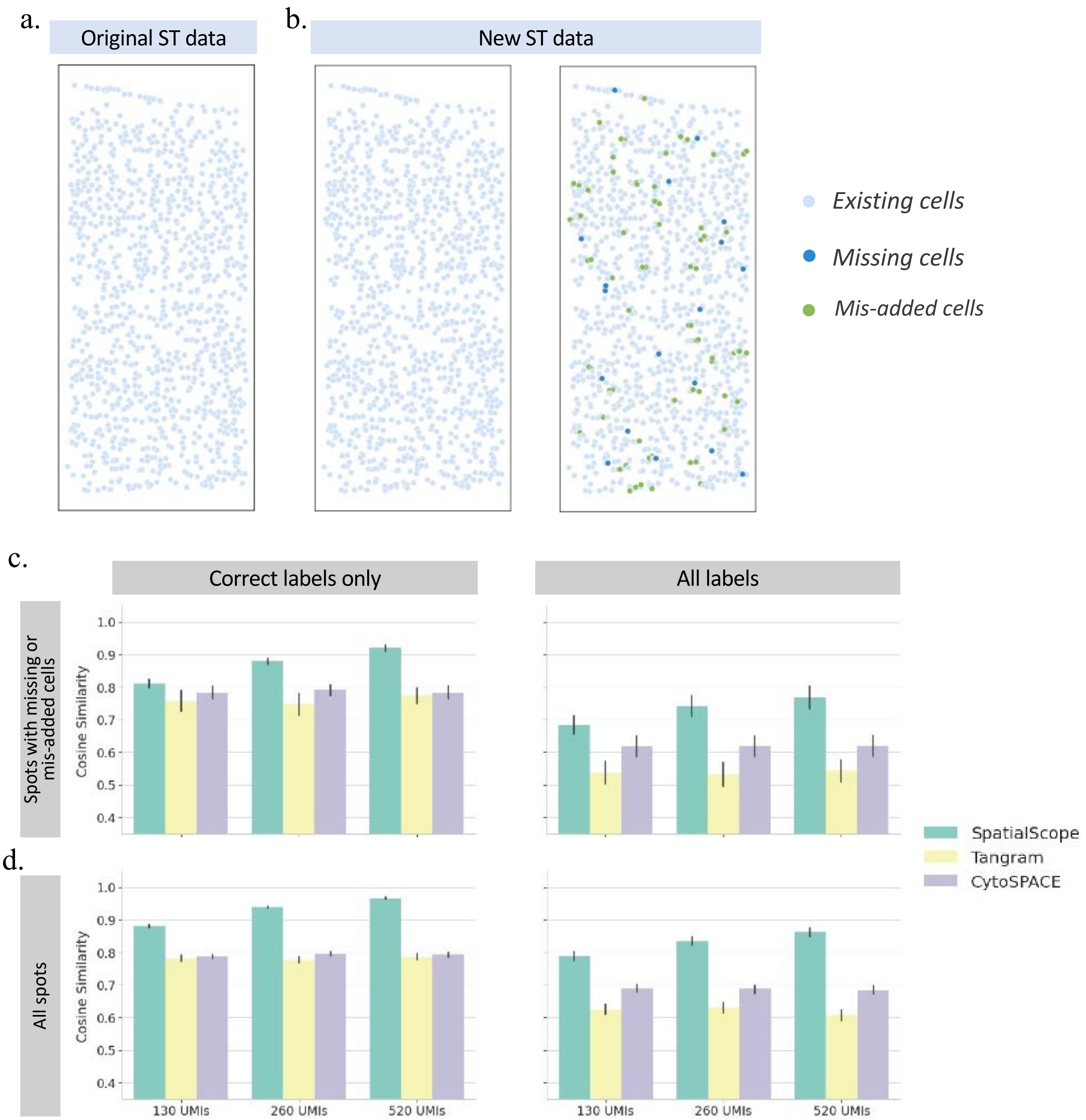

a.
Original ST data
b.
New ST data
Existing cells
Missing cells
Mis-added cells
c.
Correct labels only
All labels
Spots with missing or mis-added cells
Cosine Similarity
d.
All spots
Cosine Similarity
130 UMIs
260 UMIs
520 UMIs
SpatialScope
Tangram
CytoSPACE

Figure S53 (previous page): Influence of inconsistent cell number on gene expression decomposition task. **a**, Original ST cells in the benchmarking Dataset 1. **b**, The new ST data after randomly removing and adding some cells. Missing cells are colored blue, and mis-added cells are colored green. **c-d**, The cosine similarities between the ground truth and predicted gene expressions by the considered methods for cells in spots with inconsistent cell number (c) or all spots (d) under different scenarios of subsampled UMIs count. We further considered cells with correctly identified cell type labels (left) or all cells (right) as in the main text. Error bars represent the 95% confidence interval of cosine similarity evaluated on $n = 119, 50, 102$ cells with correct labels in spots with inconsistent cell numbers for SpatialScope, Tangram and CytoSPACE, respectively, under 130UMI setting, $n = 120, 57, 99$ cells with correct labels in spots with inconsistent cell numbers for SpatialScope, Tangram and CytoSPACE, respectively, under 260UMI setting, $n = 122, 59, 103$ cells with correct labels in spots with inconsistent cell numbers for SpatialScope, Tangram and CytoSPACE, respectively, under 520UMI setting, $n = 755, 385, 679$ cells with correct labels in all spots for SpatialScope, Tangram and CytoSPACE, respectively, under 130UMI setting, $n = 754, 413, 674$ cells with correct labels in all spots for SpatialScope, Tangram and CytoSPACE, respectively, under 260UMI setting, $n = 767, 359, 671$ cells with correct labels in all spots for SpatialScope, Tangram and CytoSPACE, respectively, under 520UMI setting, $n = 187$ cells in spots with inconsistent cell numbers, $n = 997$ for cells in all spots or spots with inconsistent cell number. Source data are provided as a Source Data file.

a.

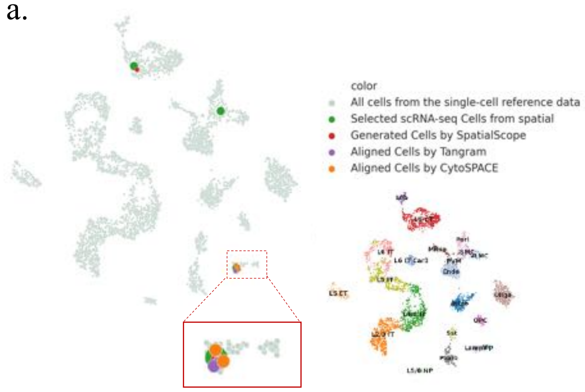

		cell1	cell2	cell3
	True label	L6 CT	Lamp5	SMC
SpatialScope	Estimated.	L6 CT	Lamp5	------
	Cos. sim.	0.94	0.96	------
Tangram	Estimated.	Lamp5	Lamp5	------
	Cos. sim.	0.38	0.80	------
CytoSPACE	Estimated.	Lamp5	Lamp5	------
	Cos. sim.	0.41	0.78	------

b.

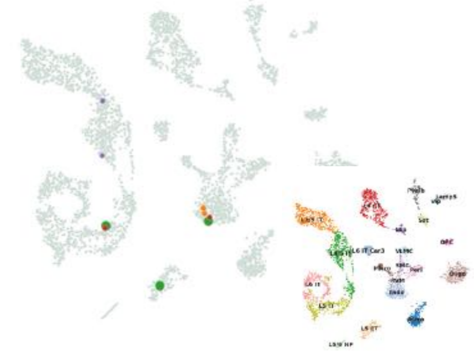

		cell1	cell2	cell3
	True label	L4/5 IT	Endo	L5 ET
SpatialScope	Estimated.	L5 IT	Endo	------
	Cos. sim.	0.94	0.88	------
Tangram	Estimated.	L4/5 IT	L4/5 IT	------
	Cos. sim.	0.60	0.16	------
CytoSPACE	Estimated.	Endo	Endo	------
	Cos. sim.	0.36	0.70	------

c.

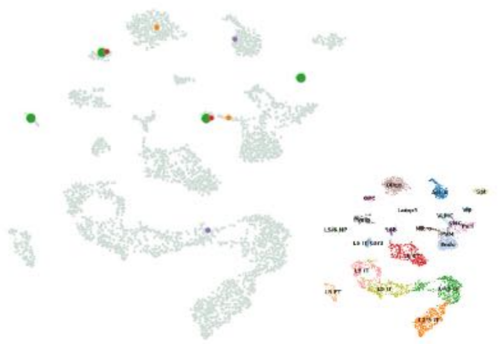

		cell1	cell2	cell3	cell4
	True label	Micro	OPC	Vip	L5/6 NP
SpatialScope	Estimated.	Micro	OPC	------	------
	Cos. sim.	0.93	0.98	------	------
Tangram	Estimated.	L4/5 IT	Astro	------	------
	Cos. sim.	0.19	0.22	------	------
CytoSPACE	Estimated.	Micro	Oligo	------	------
	Cos. sim.	0.61	0.37	------	------

d.

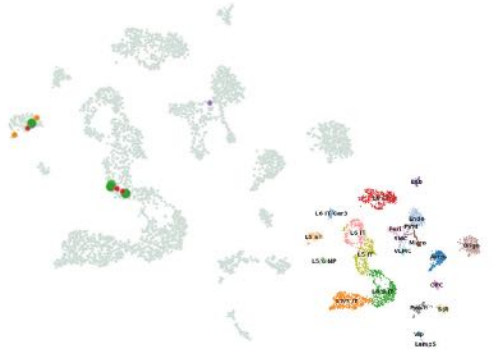

		cell1	cell2	cell3	cell4
	True label	L5 IT	L5 ET	L4/5 IT	L5 IT
SpatialScope	Estimated.	L5 IT	L5 ET	L5 IT	------
	Cos. sim.	0.86	0.89	0.90	------
Tangram	Estimated.	------	------	L5 IT	------
	Cos. sim.	------	------	0.88	------
CytoSPACE	Estimated.	L5 ET	L5 ET	L5 ET	------
	Cos. sim.	0.64	0.80	0.58	------

Figure S54: Examples of spots with missing cells in the gene expression decomposition task. **a**, The UMAP plot of gene expression decomposition example for spot 108, which contains three cells from L6 CT, Lamp5 and SMC, but the SMC cell was missing. The cell type identification and gene expression decomposition results were shown in the table below. Estimate.: Estimated cell type label by SpatialScope and the compared method. Cos. sim.: Cosine similarity between the ground truth and predicted gene expressions. **b**, The UMAP plot of gene expression decomposition example for spot 54, which contains three cells from L4/5 IT, Endo and L5 ET, but the L5 ET cell was missing. **c**, The UMAP plot of gene expression decomposition example for spot 140, which contains four cells from Micro, OPC, Vip and L5/6 NP, but the Vip and L5/6 NP cells were missing. **d**, The UMAP plot of gene expression decomposition example for spot 593, which contains four cells from L5 IT, L4/5 IT and L5 ET, but one of the L5 IT cells was missing.

a.

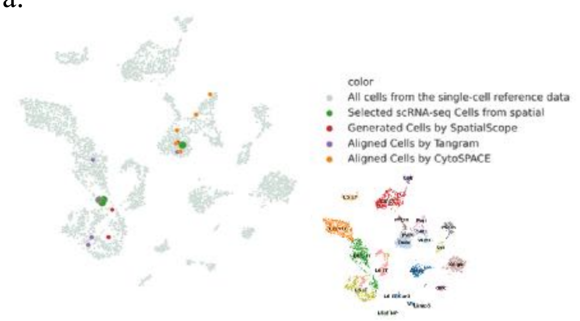

		cell1	cell2	cell3	cell4	cell5
	True label	L4/5 IT	L4/5 IT	L4/5 IT	Endo	------
SpatialScope	Estimated.	L4/5 IT	L5 IT	L4/5 IT	Endo	L4/5 IT
	Cos. sim.	0.83	0.68	0.79	0.82	------
Tangram	Estimated.	L4/5 IT	L4/5 IT	L5 IT	L5 IT	Endo
	Cos. sim.	0.81	0.60	0.68	0.18	------
CytoSPACE	Estimated.	Endo	Endo	Peri	Peri	PVM
	Cos. sim.	0.43	0.29	0.39	0.71	------

b.

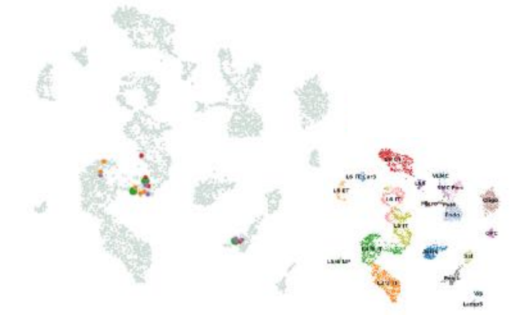

		cell1	cell2	cell3	cell4	cell5
	True label	L4/5 IT	Pvalb	L4/5 IT	------	------
SpatialScope	Estimated.	L4/5 IT	Pvalb	L5 IT	L5 IT	Pvalb
	Cos. sim.	0.76	0.90	0.73	------	------
Tangram	Estimated.	L4/5 IT	L4/5 IT	L4/5 IT	Pvalb	Pvalb
	Cos. sim.	0.71	0.38	0.42	------	------
CytoSPACE	Estimated.	L4/5 IT	L4/5 IT	L4/5 IT	L4/5 IT	L4/5 IT
	Cos. sim.	0.79	0.53	0.64	------	------

c.

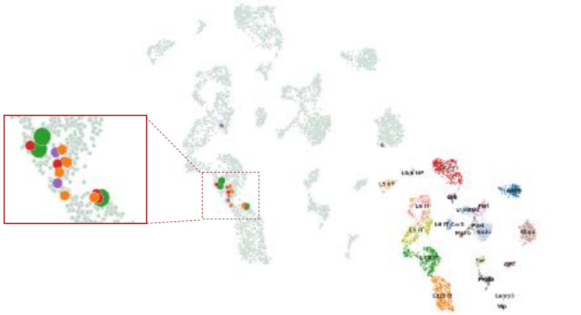

		cell1	cell2	cell3	cell4	cell5
	True label	L4/5 IT	L4/5 IT	L2/3 IT	------	------
SpatialScope	Estimated.	L2/3 IT	L4/5 IT	L2/3 IT	L4/5 IT	L4/5 IT
	Cos. sim.	0.79	0.87	0.90	------	------
Tangram	Estimated.	L4/5 IT	L4/5 IT	L5 IT	Oligo	------
	Cos. sim.	0.71	0.78	0.72	------	------
CytoSPACE	Estimated.	L2/3 IT	L4/5 IT	L4/5 IT	L4/5 IT	L4/5 IT
	Cos. sim.	0.80	0.82	0.89	------	------

d.

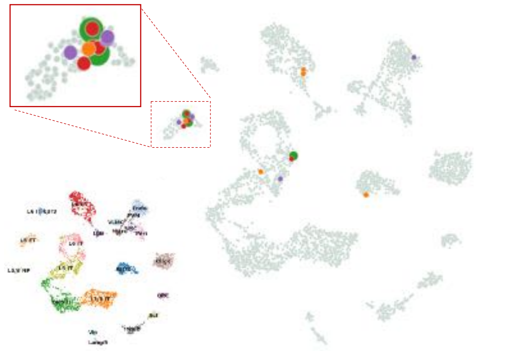

		cell1	cell2	cell3	cell4	cell5
	True label	L5 ET	L6 IT	L5 ET	------	------
SpatialScope	Estimated.	L5 ET	L5 ET	L6 IT	L5 ET	L5 ET
	Cos. sim.	0.95	0.63	0.61	------	------
Tangram	Estimated.	L5 IT	Endo	L5 ET	L5 ET	L5 ET
	Cos. sim.	0.60	0.20	0.76	------	------
CytoSPACE	Estimated.	Astro	L5 ET	L5 IT	L6 CT	L6 CT
	Cos. sim.	0.34	0.60	0.60	------	------

Figure S55: Examples of spots with mis-added cells in the gene expression decomposition task. **a**, The UMAP plot of gene expression decomposition example for spot 463, which contains four existing cells from L4/5 IT and Endo, and one mis-added cell. The cell type identification and gene expression decomposition results were shown in the table below. Estimate.: Estimated cell type label by SpatialScope and the compared method. Cos. sim.: Cosine similarity between the ground truth and predicted gene expressions. **b**, The UMAP plot of gene expression decomposition example for spot 13, which contains three existing cells from L4/5 IT and Pvalb, and two mis-added cells. **c**, The UMAP plot of gene expression decomposition example for spot 8, which contains three existing cells from L4/5 IT and L2/3 IT, and two mis-added cells. **d**, The UMAP plot of gene expression decomposition example for spot 437, which contains three existing cells from L5 ET and L6 IT, and two mis-added cells.

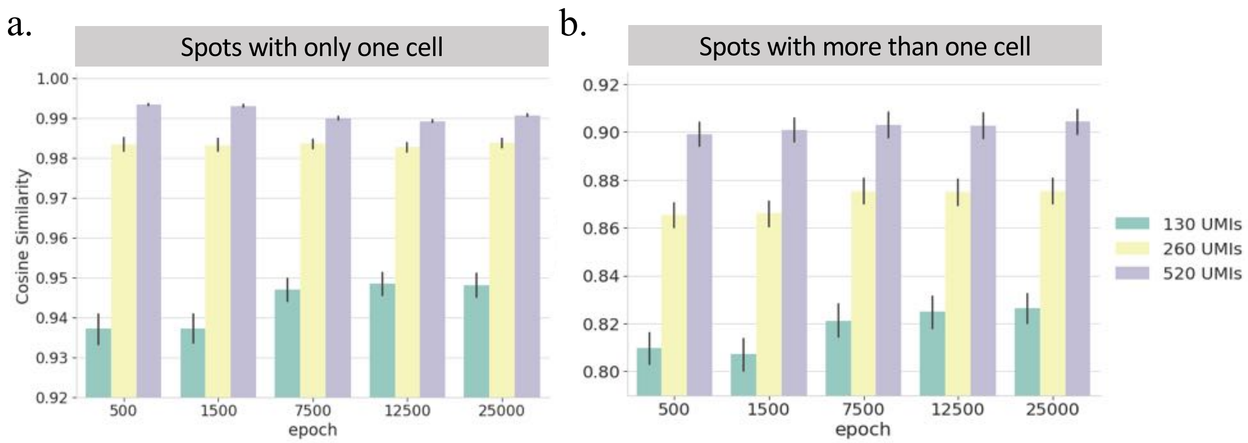

Figure S56: The gene expression decomposition performance for spots with only one cell (a) or cell number larger than one (b) when different checkpoints of the score-based generative model were used in the benchmarking Dataset 1. Error bars represent the 95% confidence interval of cosine similarity evaluated on $n = 333$ spots with only one cell or $n = 289$ spots with more than one cell.

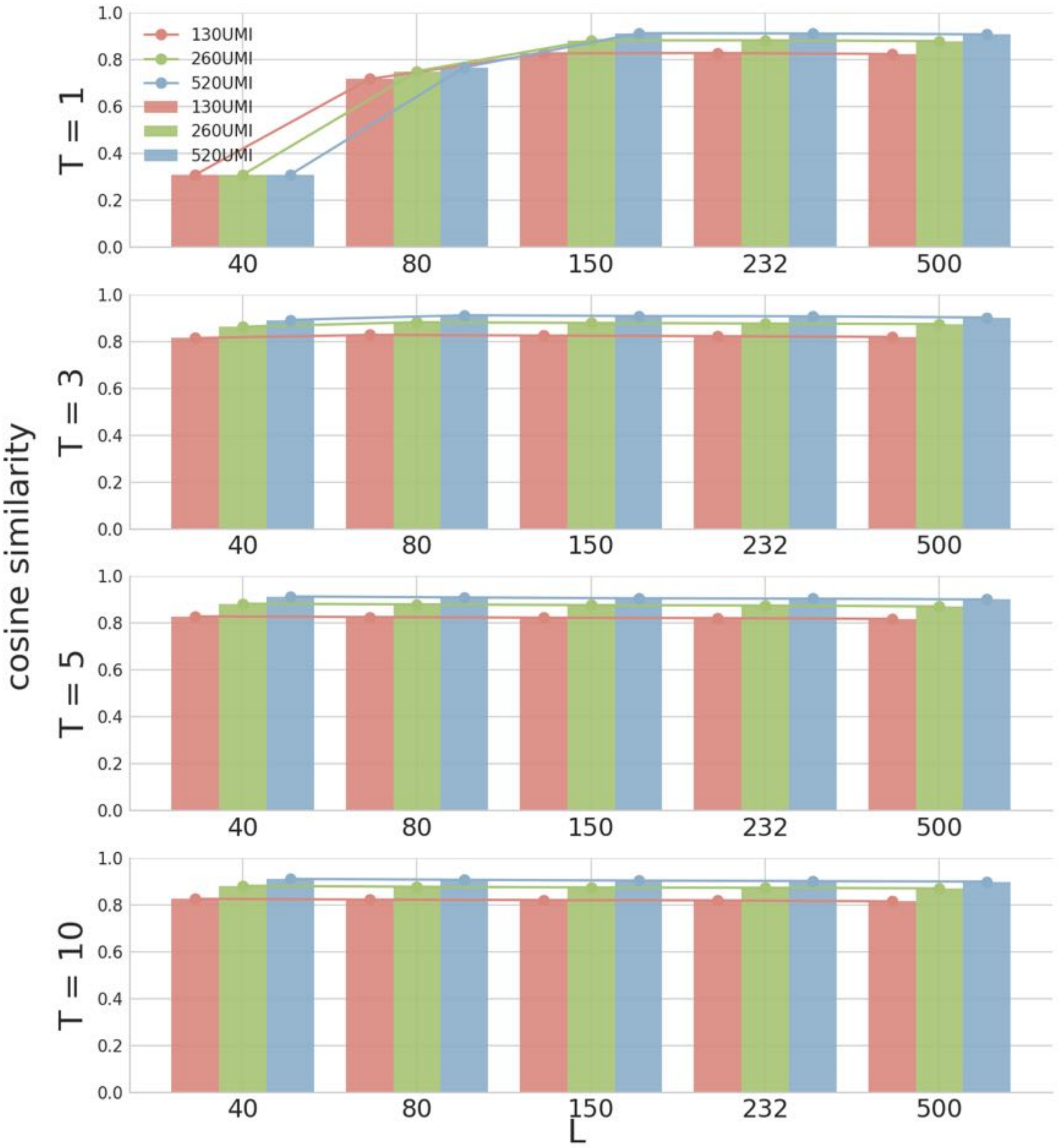

Figure S57: The evaluation of hyperparameters L and T. Bar plot of the cosine similarities between the ground truth and decomposed single-cell level gene expression profiles under different values of L and T. We use the same simulation data as in the main text. Different color represents different UMI subsample rate. The lower UMI subsample rate, the more difficult for gene expression decomposition task. Source data are provided as a Source Data file.

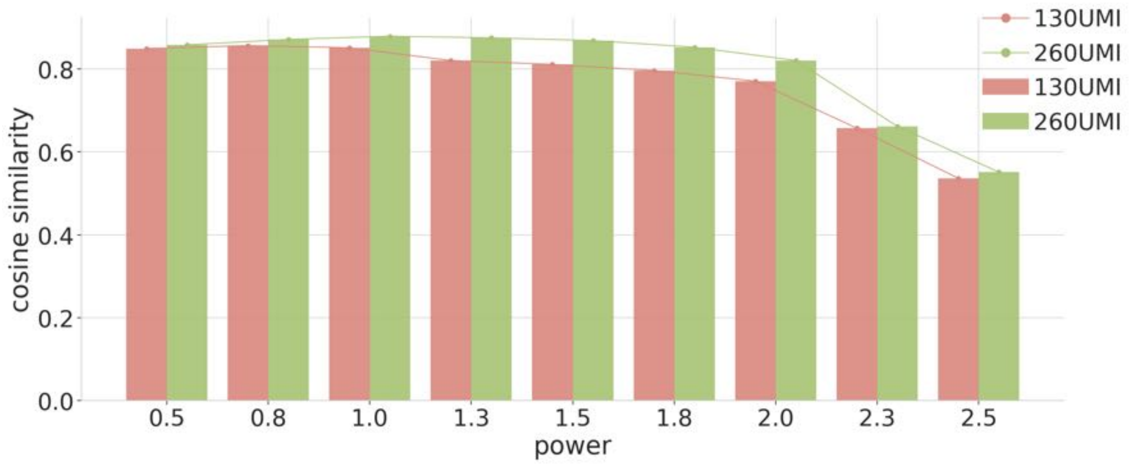

Figure S58: The evaluation of hyperparameters σ_{yl}. Bar plot of the cosine similarities between the ground truth and decomposed single-cell level gene expression profiles under different values of *power*, where $\sigma_{yl} = \sigma_l^{\frac{power}{2}}$ and σ_{yl} shows in Algorithm 1 in the main text. Different color represents different UMI subsample rate. The lower the UMI subsample rate, the more difficult for gene expression decomposition task. Source data are provided as a Source Data file.

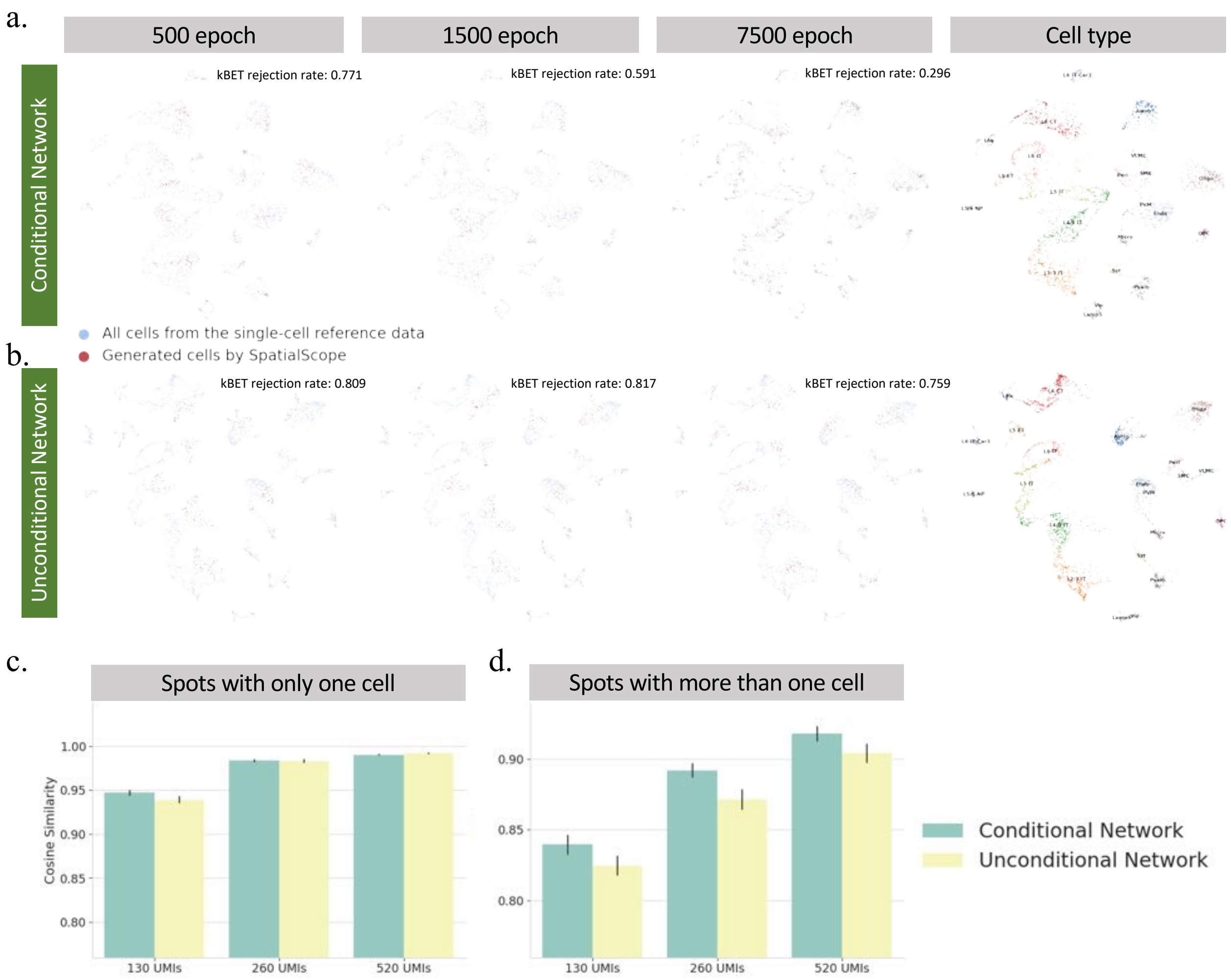

Figure S59: Comparison of conditional and unconditional networks in training the score-based generative model. The learning process of the score-based generative model for the conditional network (**a**) and unconditional network (**b**). UMAP of single cell reference data and the pseudo cells generated by the deep generative model at different epochs. The blue dots represent the existing cells from scRNA-seq data and the red dots represent cells generated by SpatialScope. **c**, Gene expression decomposition performance between conditional and unconditional networks for spots with only one cell. **d**, Gene expression decomposition performance for spots with cell number larger than one. Error bars represent the 95% confidence interval of cosine similarity evaluated on $n = 333$ spots with only one cell or 289 spots with more than one cell.

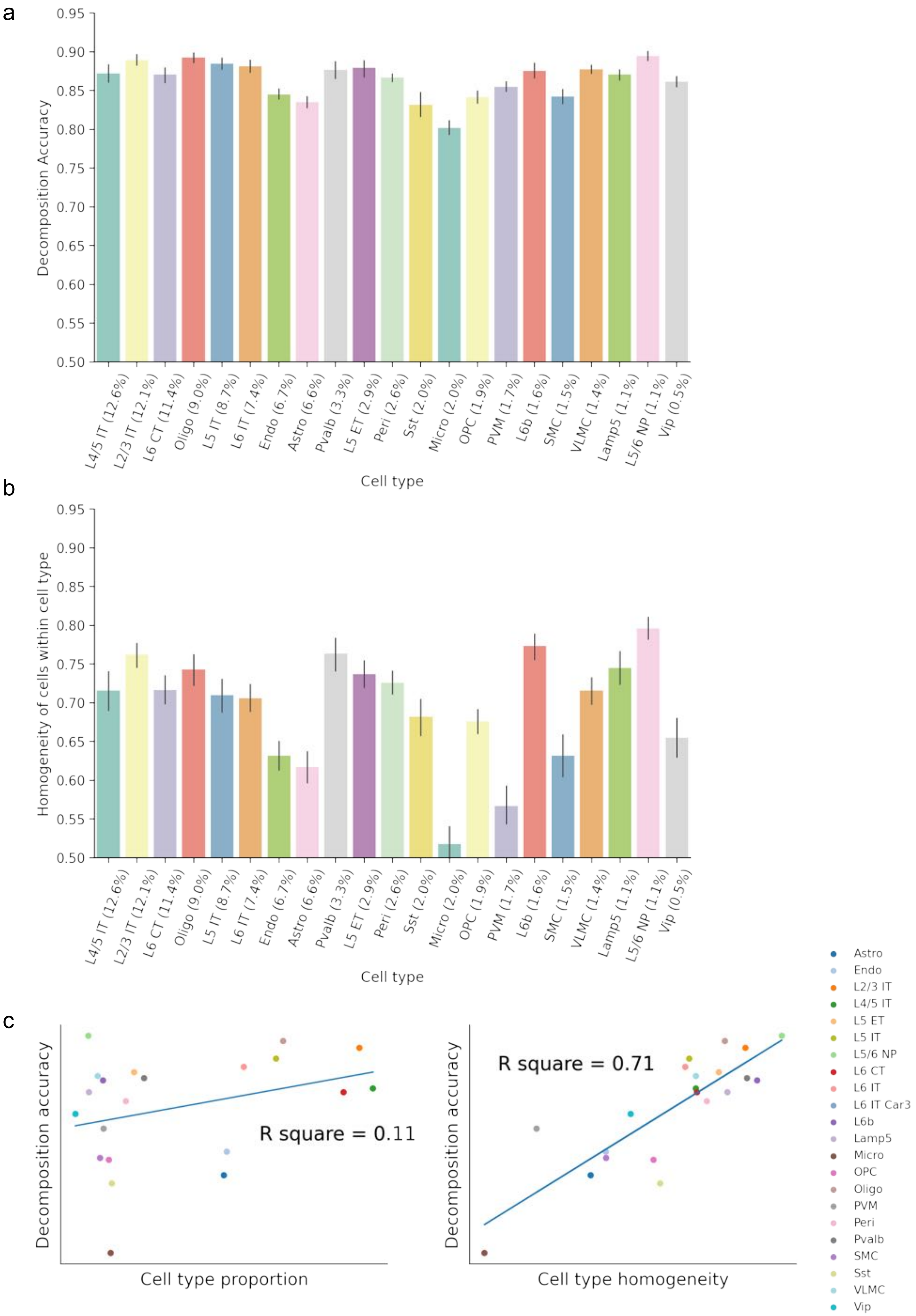
a
Decomposition Accuracy
0.95
0.90
0.85
0.80
0.75
0.70
0.65
0.60
0.55
0.50
L4/5 IT (12.6%)
L2/3 IT (12.1%)
L6 CT (11.4%)
Oligo (9.0%)
L5 IT (8.7%)
L6 IT (7.4%)
Endo (6.7%)
Astro (6.6%)
Pvalb (3.3%)
L5 ET (2.9%)
Peri (2.6%)
Sst (2.0%)
Micro (2.0%)
OPC (1.9%)
PVM (1.7%)
L6b (1.6%)
SMC (1.5%)
VLMC (1.4%)
Lamp5 (1.1%)
L5/6 NP (1.1%)
Vip (0.5%)
Cell type
b
Homogeneity of cells within cell type
0.95
0.90
0.85
0.80
0.75
0.70
0.65
0.60
0.55
0.50
L4/5 IT (12.6%)
L2/3 IT (12.1%)
L6 CT (11.4%)
Oligo (9.0%)
L5 IT (8.7%)
L6 IT (7.4%)
Endo (6.7%)
Astro (6.6%)
Pvalb (3.3%)
L5 ET (2.9%)
Peri (2.6%)
Sst (2.0%)
Micro (2.0%)
OPC (1.9%)
PVM (1.7%)
L6b (1.6%)
SMC (1.5%)
VLMC (1.4%)
Lamp5 (1.1%)
L5/6 NP (1.1%)
Vip (0.5%)
Cell type
c
Decomposition accuracy
R square = 0.11
Cell type proportion
Decomposition accuracy
R square = 0.71
Cell type homogeneity
Astro
Endo
L2/3 IT
L4/5 IT
L5 ET
L5 IT
L5/6 NP
L6 CT
L6 IT
L6 IT Car3
L6b
Lamp5
Micro
OPC
Oligo
PVM
Peri
Pvalb
SMC
Sst
VLMC
Vip

Figure S60 (previous page): Influence of unbalanced cell types in training the score-based generative model. **a**, Gene expression decomposition performance of SpatialScope for each cell type in the MERFISH simulation dataset, the cell types are sorted by their proportion (shown in the x-ticks) in the single-cell reference data. Error bars represent the 95% confidence interval of cosine similarity evaluated on $n = 4000$ decomposed cells' gene expression levels from different cell types. **b**, The mean cosine similarities between two randomly selected cells for each cell type in the paired single-cell reference of the MERFISH simulation dataset. Error bars represent the 95% confidence interval of cosine similarity evaluated on $n = 100$ cell pairs from different cell types. **c**, Left, Linear regression of decomposition accuracy within a cell type on cell type proportion. Right, Linear regression of decomposition accuracy within a cell type on cell type homogeneity. Different colors represent the cell type of the data point. Source data are provided as a Source Data file.

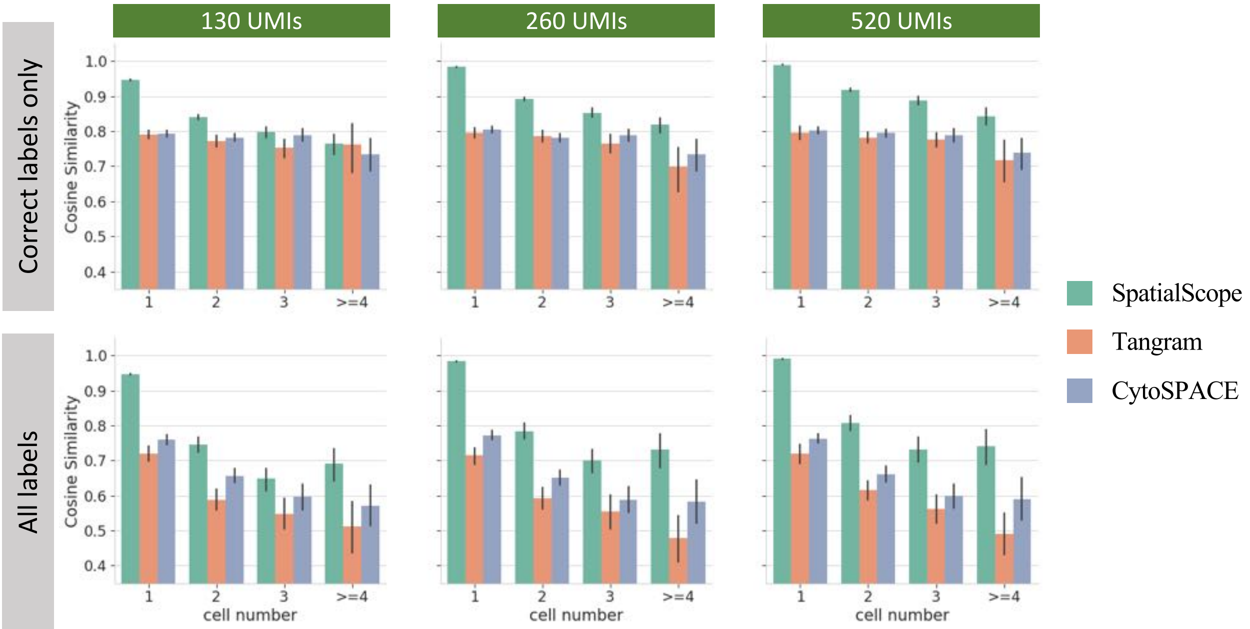

Figure S61: Comparison of gene expression decomposition for spots with varying cell numbers when reference is paired. The cosine similarities between the ground truth and predicted gene expressions for cells with correctly identified cell type label (top) or all cells (bottom) under different combination scenarios of UMI subsample rate. Error bars represent the 95% confidence interval of cosine similarity evaluated on 599 spots with varying cell numbers.

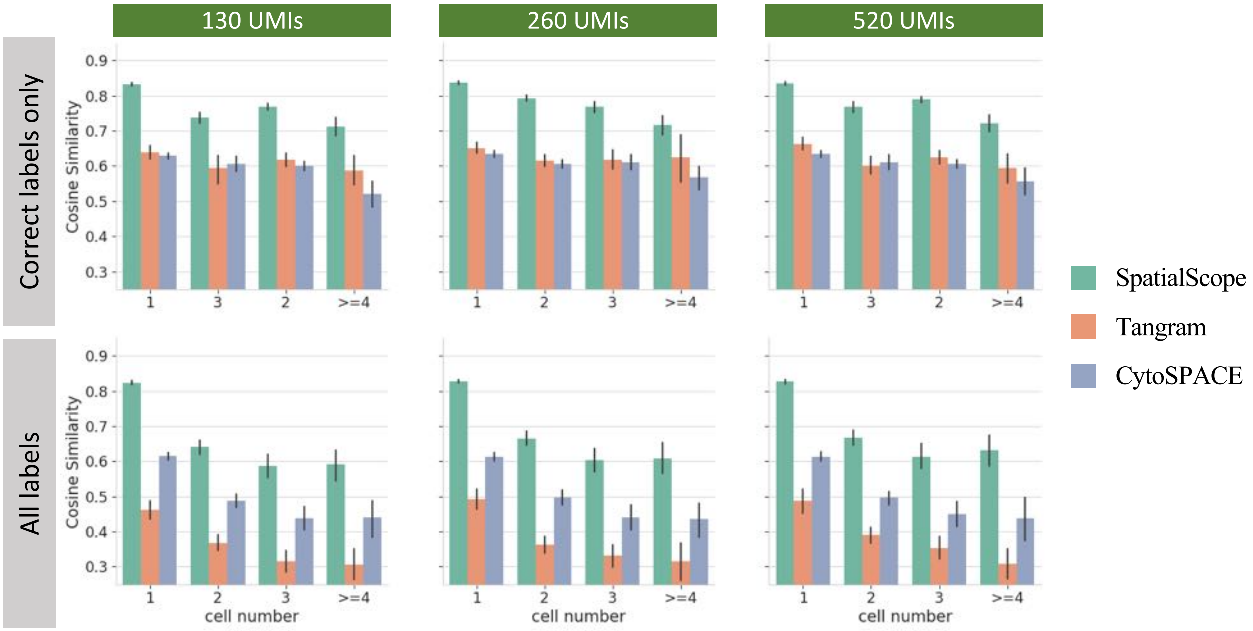

Figure S62: Comparison of gene expression decomposition for spots with varying cell numbers when reference is unpaired. The cosine similarities between the ground truth and predicted gene expressions for cells with correctly identified cell type label (top) or all cells (bottom) under different combination scenarios of UMI subsample rate. Error bars represent the 95% confidence interval of cosine similarity evaluated on 599 spots with varying cell numbers.

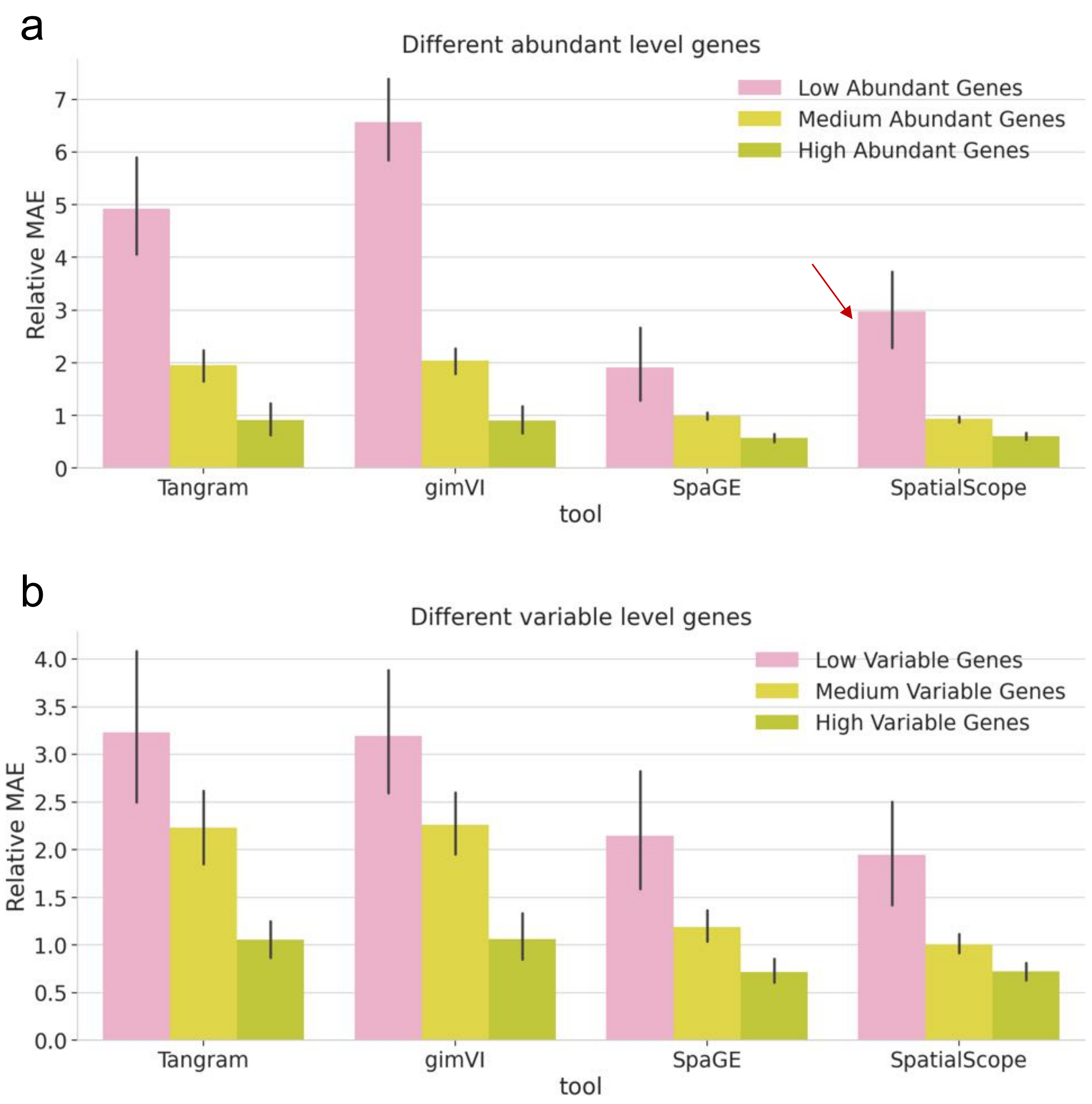

Figure S63: Performance of different methods imputing different gene groups with metric Relative MAE. a, Bar plot of Relative MAE from methods Tangram, gimVI, SpaGE, SpatialScope imputing low, medium and high abundant genes. Data are presented as mean values ±95% confidence intervals; $n = 25$ selected genes in each group. **b**, Bar plot of Relative MAE from methods Tangram, gimVI, SpaGE, SpatialScope imputing low, medium and high variable genes. Data are presented as mean values ±95% confidence intervals; $n = 25$ selected genes in each group. Source data are provided as a Source Data file.

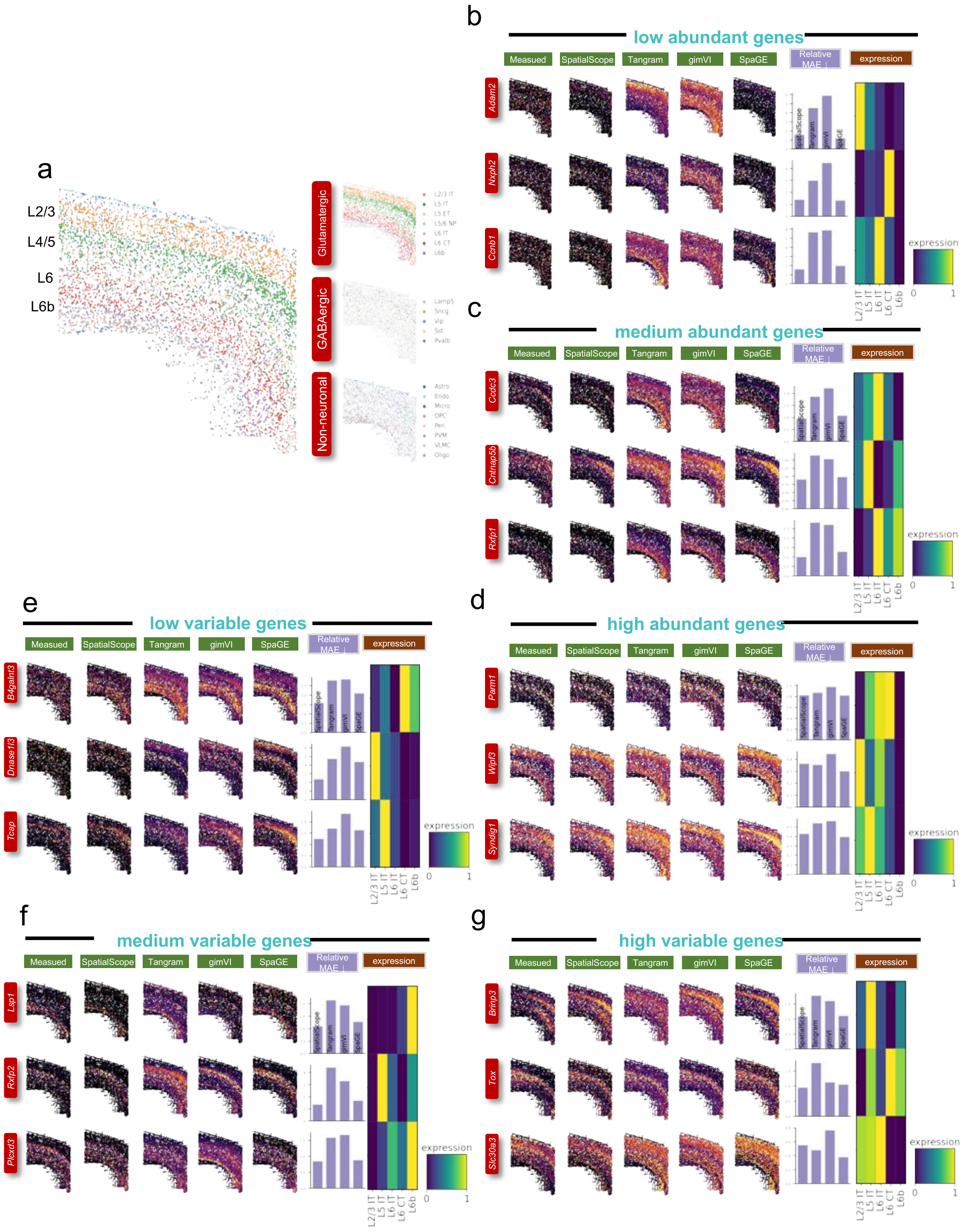

a
L2/3
L4/5
L6
L6b
Glutamatergic
GABAergic
Non-neuronal
b
low abundant genes
Measued
SpatialScope
Tangram
gimVI
SpaGE
Relative MAE ↓
expression
Adam2
Nxph2
Ccnb1
c
medium abundant genes
Ccdc3
Cntnap5b
Rxfp1
d
high abundant genes
Parm1
Wipf3
Syndig1
e
low variable genes
B4galnt3
Dnase1l3
Tcap
f
medium variable genes
Lsp1
Rxfp2
Plcxd3
g
high variable genes
Brinp3
Tox
Slc30a3
L2/3 IT
L5 IT
L6 IT
L6 CT
L6b
0
1

Figure S64 (previous page): The results of different methods predicting low, medium, and high abundant genes and low, medium, and high variable genes. a, Cell type identification results of MERFISH MOp data by SpatialScope showing a clear layer structure of mouse brain. Cell type identification results in each of the three major categories are shown on the right. **a,b,c,d,e,f,g** shows the prediction results of low abundant genes, medium abundant genes, high abundant genes, low variable genes, medium variable genes, and high variable genes, respectively. In each group, the figure shows measured and imputed expressions of selected three genes in that group. Each row corresponds to a single gene. The first column from the left shows the measured spatial gene expression in the MERFISH dataset, while the second to fifth columns show the corresponding imputed expression pattern by SpatialScope, Tangram, gimVI, SpaGE. The imputation accuracy was evaluated by Relative MAE and displayed with bar plots (sixth column). The marker gene expression signatures in snRNA-seq reference were displayed with a heatmap plot (seventh column).

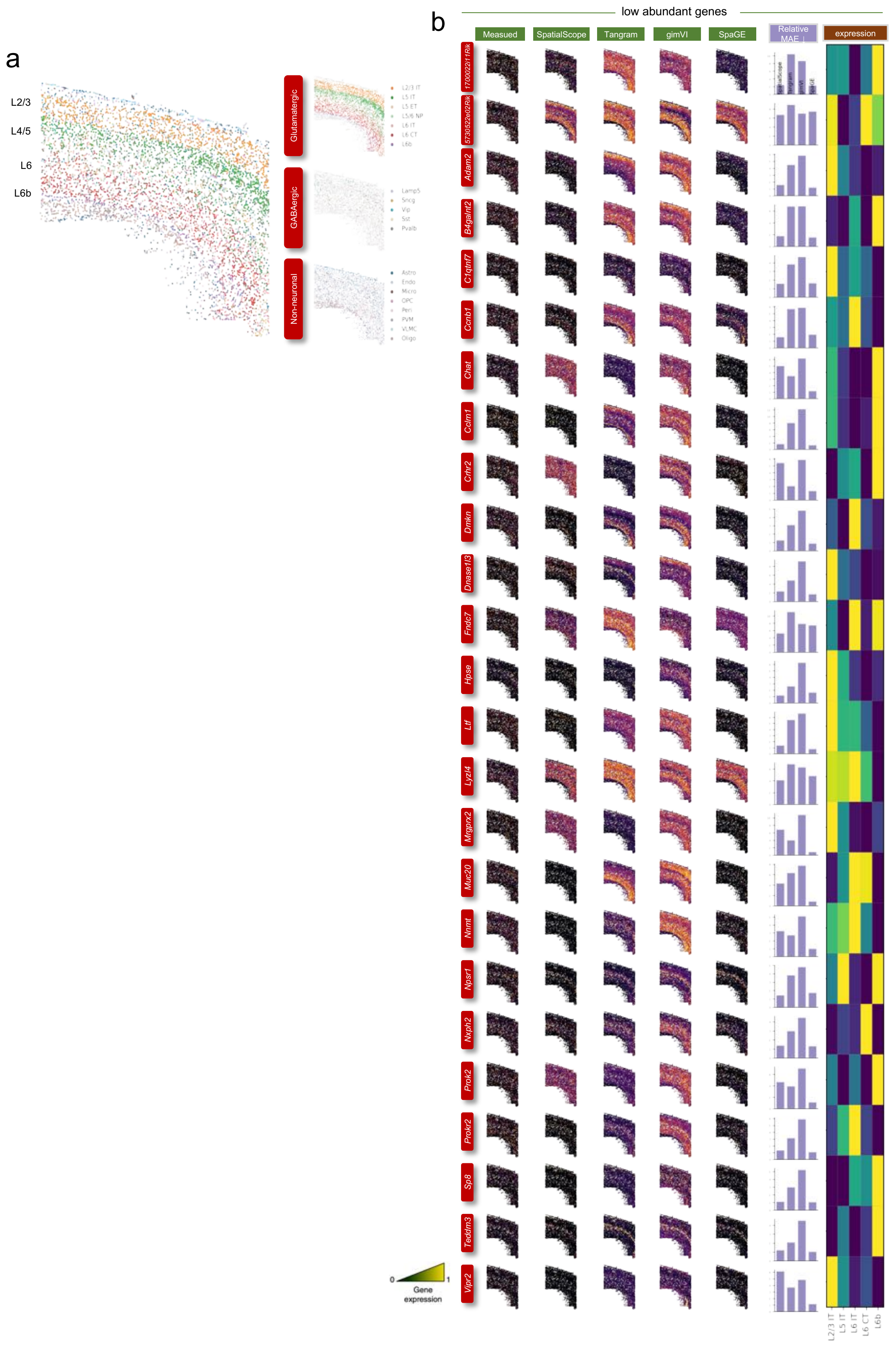
a
L2/3
L4/5
L6
L6b
Glutamatergic
GABAergic
Non-neuronal
L2/3 IT
L5 IT
L5 ET
L5/6 NP
L6 IT
L6 CT
L6b
Lamp5
Sncg
Vip
Sst
Pvalb
Astro
Endo
Micro
OPC
Peri
PVM
VLMC
Oligo
b
low abundant genes
Measued
SpatialScope
Tangram
gimVI
SpaGE
Relative MAE
expression
1700022I11Rik
5730522e02Rik
Adam2
B4galnt2
C1qtnf7
Ccnb1
Chat
Cdlm1
Crhr2
Dmkn
Dnase1l3
Fndc7
Hpse
Ltf
Lyzl4
Mrgprx2
Muc20
Nnmt
Npsr1
Nxph2
Prok2
Prokr2
Sp8
Teddm3
Vipr2
0
1
Gene expression
L2/3 IT
L5 IT
L6 IT
L6 CT
L6b

Figure S65 (previous page): Different methods predicting low abundant genes. a, Cell type identification results of MERFISH MOp data by SpatialScope showing a clear layer structure of mouse brain. Cell type identification results in each of the three major categories are shown on the right. **b**, The prediction results of 25 low abundant genes. Each row corresponds to a single gene. The first column from the left shows the measured spatial gene expression in the MERFISH dataset, while the second to fifth columns show the corresponding imputed expression pattern by SpatialScope, Tangram, gimVI, SpaGE. The imputation accuracy was evaluated by Relative MAE and displayed with bar plots (sixth column). The marker gene expression signatures in snRNA-seq reference were displayed with a heatmap plot (seventh column).

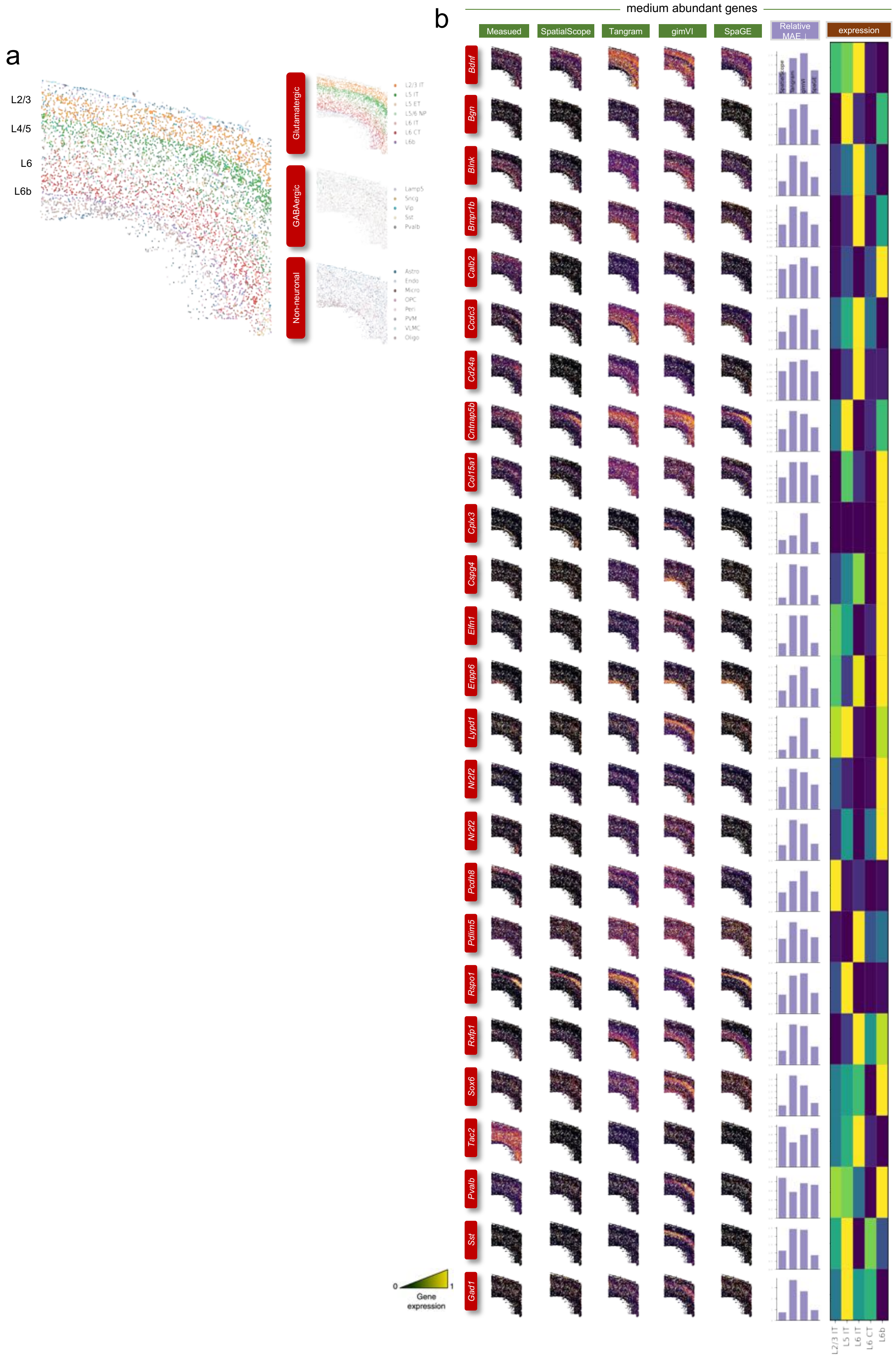
a
L2/3
L4/5
L6
L6b
Glutamatergic
L2/3 IT
L5 IT
L5 ET
L5/6 NP
L6 IT
L6 CT
L6b
GABAergic
Lamp5
Sncg
Vip
Sst
Pvalb
Non-neuronal
Astro
Endo
Micro
OPC
Peri
PVM
VLMC
Oligo
b
medium abundant genes
Measued
SpatialScope
Tangram
gimVI
SpaGE
Relative MAE
expression
Bdnf
Bgn
Blnk
Bmpr1b
Calb2
Ccdc3
Cd24a
Cntnap5b
Col15a1
Cplx3
Cspg4
Elfn1
Enpp6
Lypd1
Nr2f2
Nr2f2
Pcdh8
Pdlim5
Rspo1
Rxfp1
Sox6
Tac2
Pvalb
Sst
Gad1
0
1
Gene expression
L2/3 IT
L5 IT
L6 IT
L6 CT
L6b

Figure S66 (previous page): Different methods predicting medium abundant genes. **a**, Cell type identification results of MERFISH MOp data by SpatialScope showing a clear layer structure of mouse brain. Cell type identification results in each of the three major categories are shown on the right. **b**, The prediction results of 25 medium abundant genes. Each row corresponds to a single gene. The first column from the left shows the measured spatial gene expression in the MERFISH dataset, while the second to fifth columns show the corresponding imputed expression pattern by SpatialScope, Tangram, gimVI, SpaGE. The imputation accuracy was evaluated by Relative MAE and displayed with bar plots (sixth column). The marker gene expression signatures in snRNA-seq reference were displayed with a heatmap plot (seventh column).

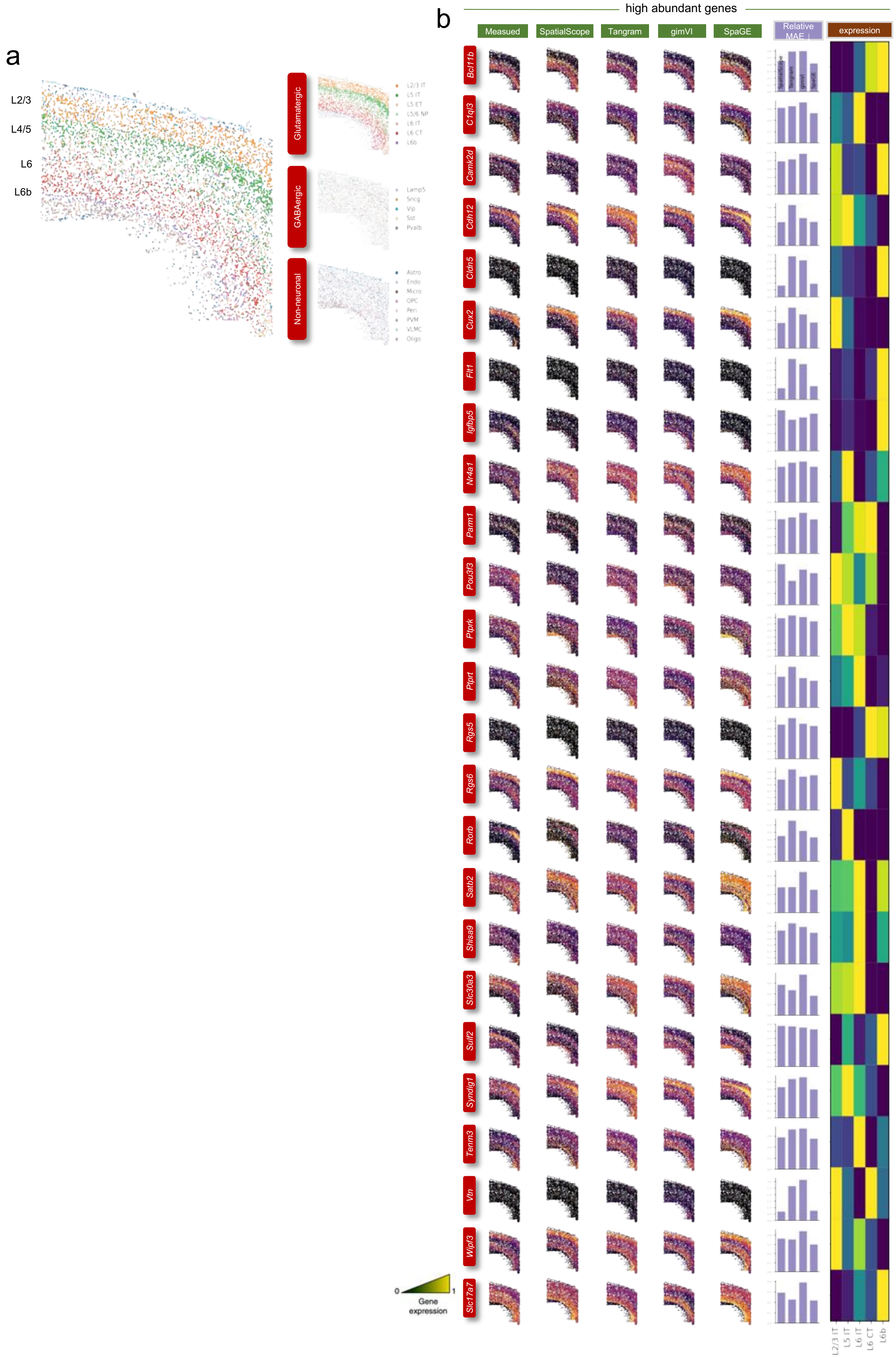
a
L2/3
L4/5
L6
L6b
Glutamatergic
GABAergic
Non-neuronal
L2/3 IT
L5 IT
L5 ET
L5/6 NP
L6 IT
L6 CT
L6b
Lamp5
Sncg
Vip
Sst
Pvalb
Astro
Endo
Micro
OPC
Peri
PVM
VLMC
Oligo
b
high abundant genes
Measued
SpatialScope
Tangram
gimVI
SpaGE
Relative MAE
expression
Bcl11b
C1ql3
Camk2d
Cdh12
Cldn5
Cux2
Flt1
Igfbp5
Nr4a1
Parm1
Pou3f3
Ptprk
Ptprt
Rgs5
Rgs6
Rorb
Satb2
Shisa9
Slc30a3
Sulf2
Syndig1
Tenm3
Vtn
Wipf3
Slc17a7
0
1
Gene expression
L2/3 IT
L5 IT
L6 IT
L6 CT
L6b

Figure S67 (previous page): Different methods predicting medium abundant genes. **a**, Cell type identification results of MERFISH MOp data by SpatialScope showing a clear layer structure of mouse brain. Cell type identification results in each of the three major categories are shown on the right. **b**, The prediction results of 25 high abundant genes. Each row corresponds to a single gene. The first column from the left shows the measured spatial gene expression in the MERFISH dataset, while the second to fifth columns show the corresponding imputed expression pattern by SpatialScope, Tangram, gimVI, SpaGE. The imputation accuracy was evaluated by Relative MAE and displayed with bar plots (sixth column). The marker gene expression signatures in snRNA-seq reference were displayed with a heatmap plot (seventh column).

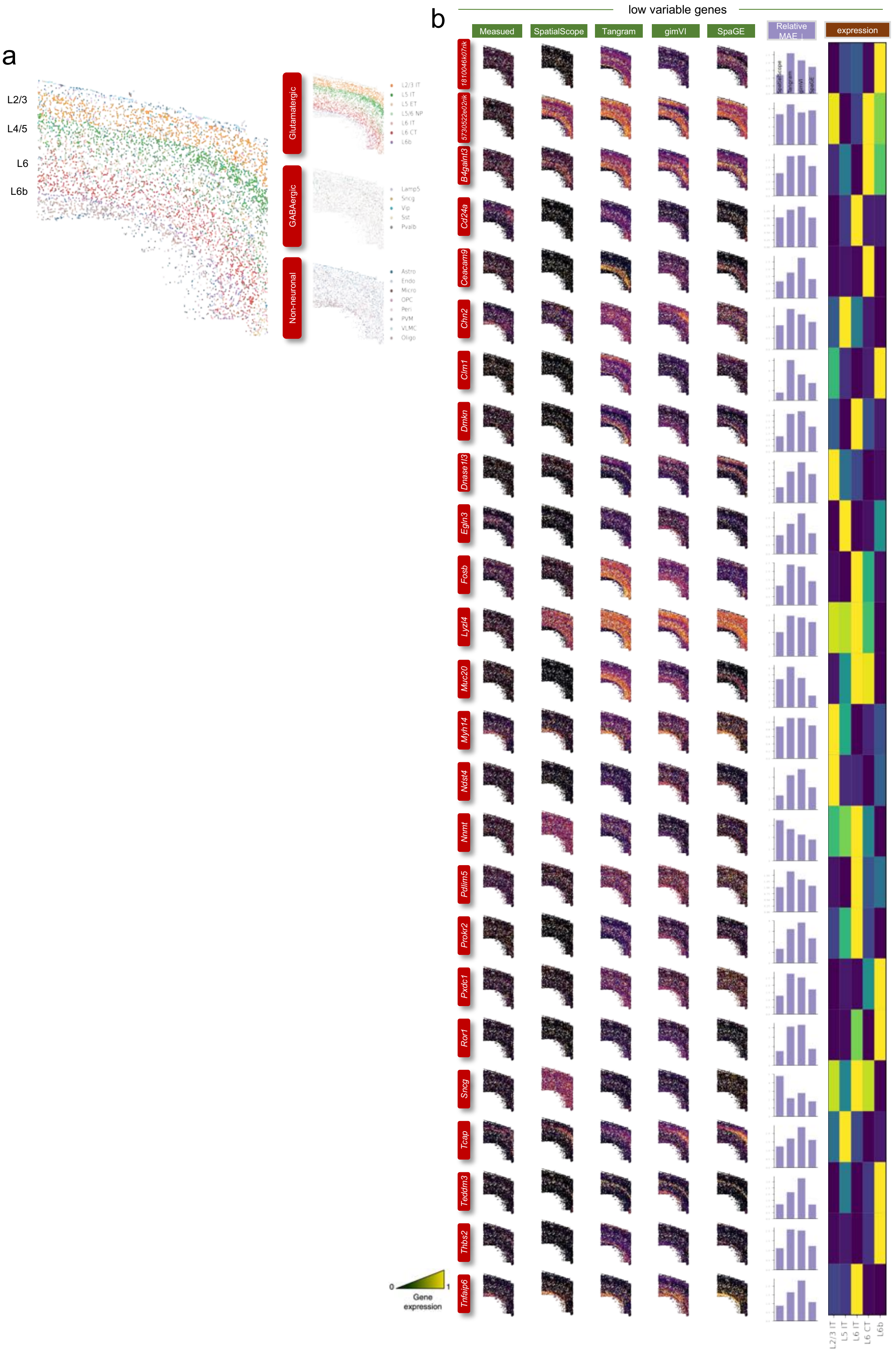
a
L2/3
L4/5
L6
L6b
Glutamatergic
L2/3 IT
L5 IT
L5 ET
L5/6 NP
L6 IT
L6 CT
L6b
GABAergic
Lamp5
Sncg
Vip
Sst
Pvalb
Non-neuronal
Astro
Endo
Micro
OPC
Peri
PVM
VLMC
Oligo
b
low variable genes
Measued
SpatialScope
Tangram
gimVI
SpaGE
Relative MAE
expression
1810046k07rik
5730522e02rik
B4galnt3
Cd24a
Ceacam9
Chn2
Clm1
Dmkn
Dnase1l3
Egln3
Fosb
Lyzl4
Muc20
Myh14
Ndst4
Nnmt
Pdlim5
Prokr2
Pxdc1
Ror1
Sncg
Tcap
Teddm3
Thbs2
Tnfaip6
0
1
Gene expression
L2/3 IT
L5 IT
L6 IT
L6 CT
L6b

Figure S68 (previous page): Different methods predicting low variable genes. a, Cell type identification results of MERFISH MOp data by SpatialScope showing a clear layer structure of mouse brain. Cell type identification results in each of the three major categories are shown on the right. **b**, The prediction results of 25 low variable genes. Each row corresponds to a single gene. The first column from the left shows the measured spatial gene expression in the MERFISH dataset, while the second to fifth columns show the corresponding imputed expression pattern by SpatialScope, Tangram, gimVI, SpaGE. The imputation accuracy was evaluated by Relative MAE and displayed with bar plots (sixth column). The marker gene expression signatures in snRNA-seq reference were displayed with a heatmap plot (seventh column).

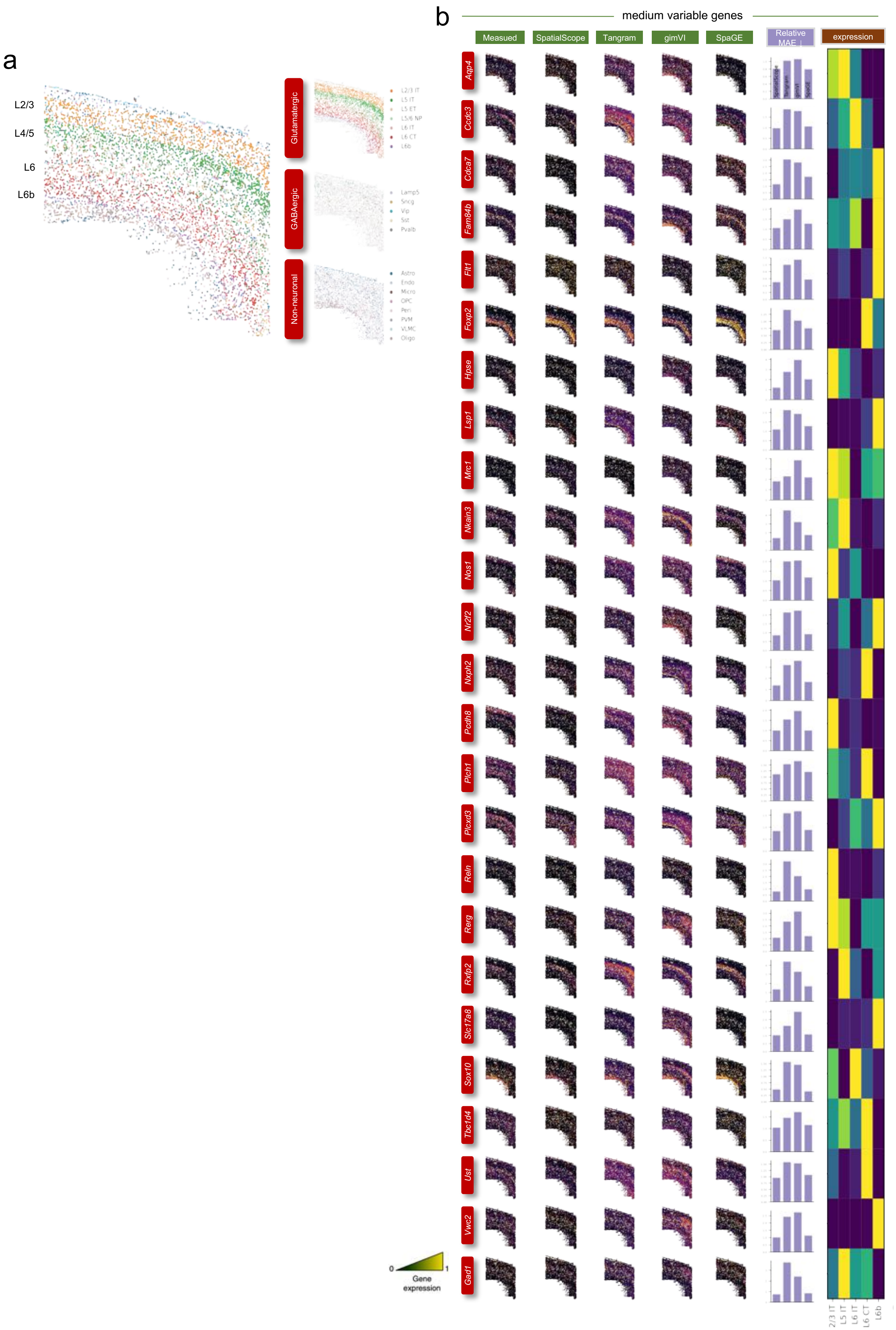
a
L2/3
L4/5
L6
L6b
Glutamatergic
L2/3 IT
L5 IT
L5 ET
L5/6 NP
L6 IT
L6 CT
L6b
GABAergic
Lamp5
Sncg
Vip
Sst
Pvalb
Non-neuronal
Astro
Endo
Micro
OPC
Peri
PVM
VLMC
Oligo
b
medium variable genes
Measued
SpatialScope
Tangram
gimVI
SpaGE
Relative MAE
expression
Aqp4
Ccdc3
Cdca7
Fam84b
Flt1
Foxp2
Hpse
Lsp1
Mrc1
Nkain3
Nos1
Nr2f2
Nxph2
Pcdh8
Plch1
Plcxd3
Reln
Rerg
Rxfp2
Slc17a8
Sox10
Tbc1d4
Ust
Vwc2
Gad1
0
1
Gene expression
2/3 IT
L5 IT
L6 IT
L6 CT
L6b

Figure S69 (previous page): Different methods predicting medium variable genes. **a**, Cell type identification results of MERFISH MOp data by SpatialScope showing a clear layer structure of mouse brain. Cell type identification results in each of the three major categories are shown on the right. **b**, The prediction results of 25 medium variable genes. Each row corresponds to a single gene. The first column from the left shows the measured spatial gene expression in the MERFISH dataset, while the second to fifth columns show the corresponding imputed expression pattern by SpatialScope, Tangram, gimVI, SpaGE. The imputation accuracy was evaluated by Relative MAE and displayed with bar plots (sixth column). The marker gene expression signatures in snRNA-seq reference were displayed with a heatmap plot (seventh column).

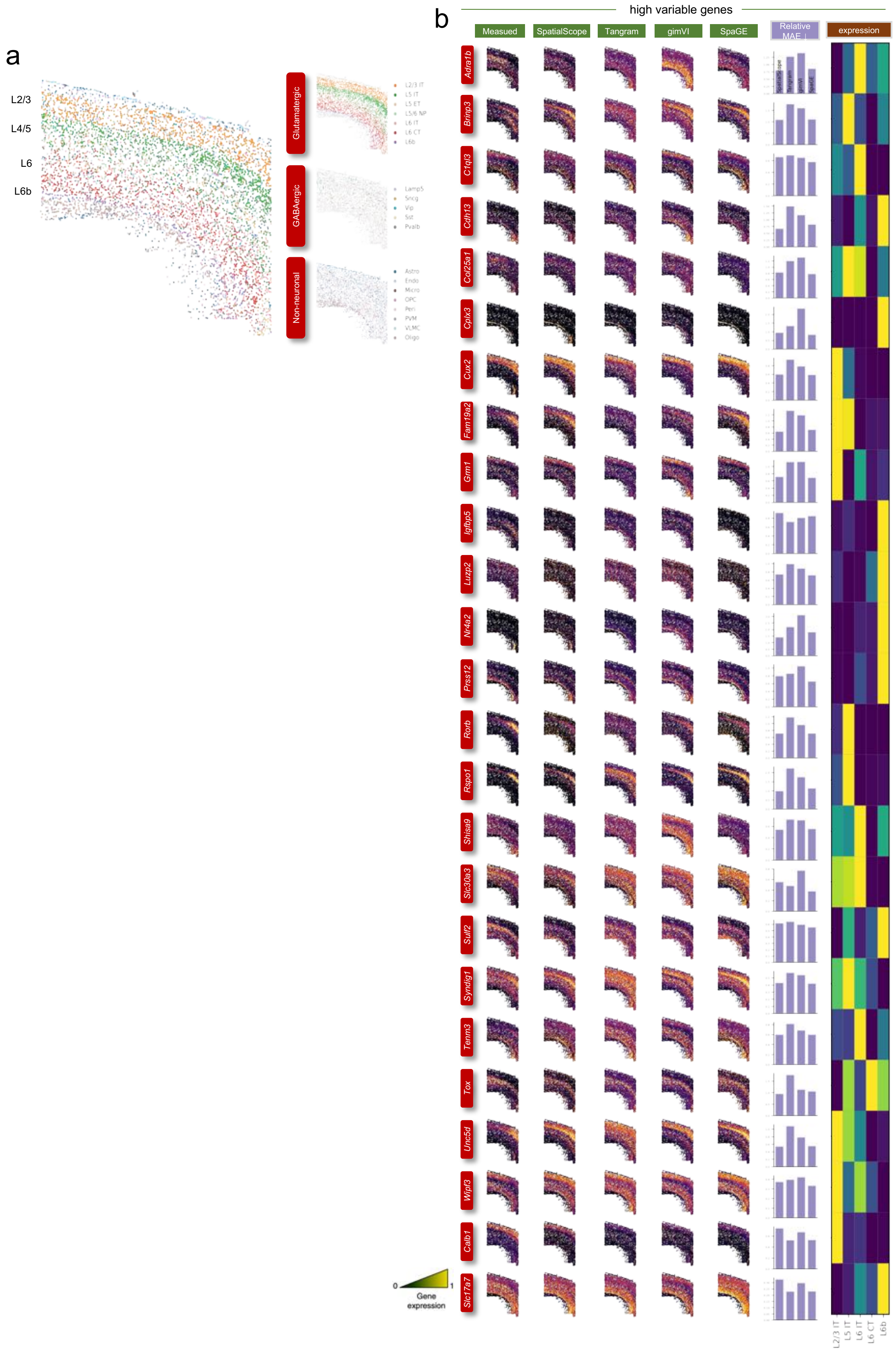
a
L2/3
L4/5
L6
L6b
Glutamatergic
GABAergic
Non-neuronal
b
high variable genes
Measued
SpatialScope
Tangram
gimVI
SpaGE
Relative MAE
expression
Adra1b
Brinp3
C1ql3
Cdh13
Col25a1
Cplx3
Cux2
Fam19a2
Grm1
Igfbp5
Luzp2
Nr4a2
Prss12
Rorb
Rspo1
Shisa9
Slc30a3
Sulf2
Syndig1
Tenm3
Tox
Unc5d
Wipf3
Calb1
Slc17a7
0
1
Gene expression
L2/3 IT
L5 IT
L6 IT
L6 CT
L6b

Figure S70 (previous page): Different methods predicting high variable genes. a, Cell type identification results of MERFISH MOp data by SpatialScope showing a clear layer structure of mouse brain. Cell type identification results in each of the three major categories are shown on the right. **b**, The prediction results of 25 high variable genes. Each row corresponds to a single gene. The first column from the left shows the measured spatial gene expression in the MERFISH dataset, while the second to fifth columns show the corresponding imputed expression pattern by SpatialScope, Tangram, gimVI, SpaGE. The imputation accuracy was evaluated by Relative MAE and displayed with bar plots (sixth column). The marker gene expression signatures in snRNA-seq reference were displayed with a heatmap plot (seventh column).

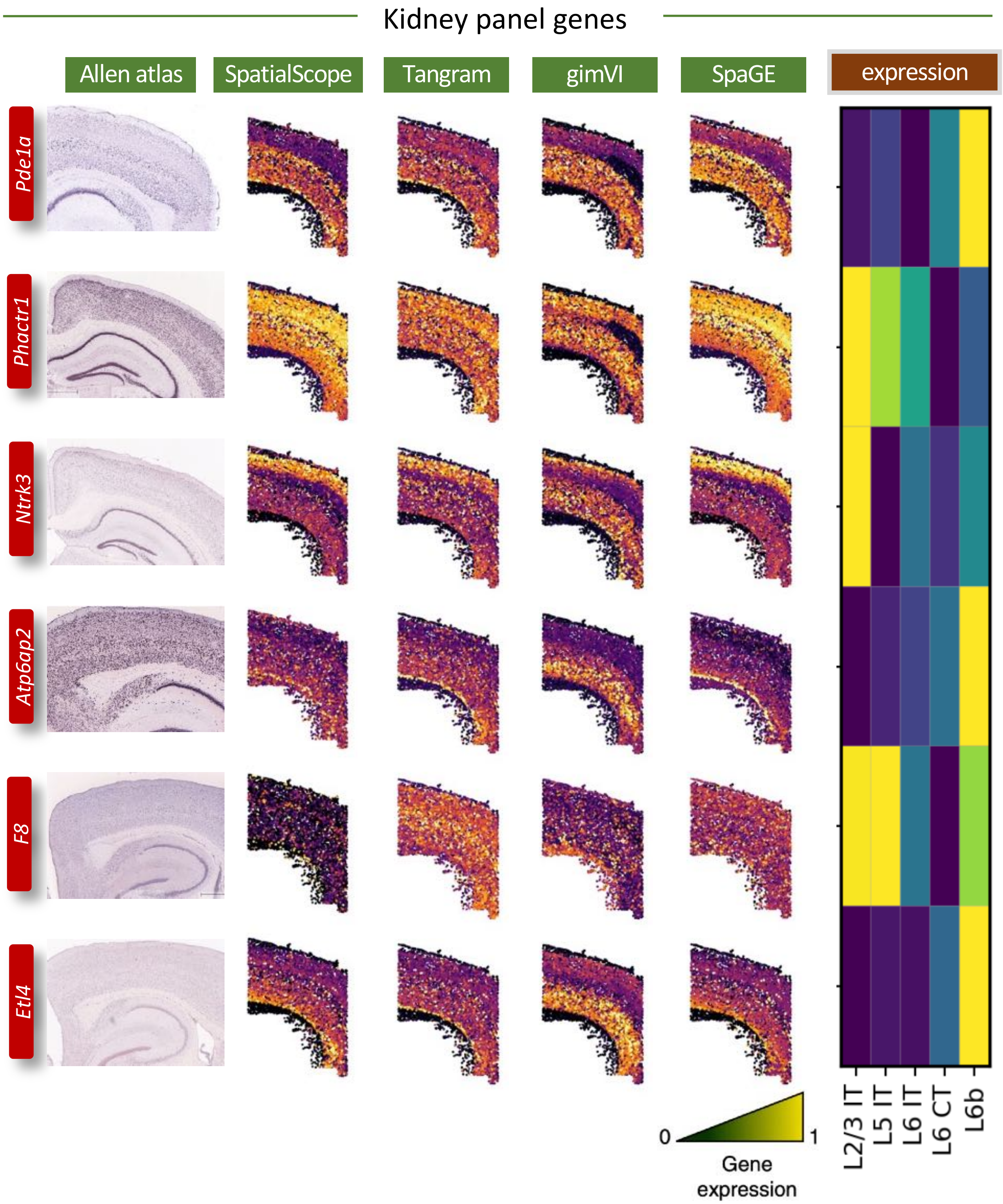

Figure S71: Measured and imputed expressions of non-MERFISH kidney panel genes. Each row corresponds to a single gene. The first column from the left displays the ISH images from the Allen Brain Atlas, while the second to fifth columns show the corresponding imputed expression patterns by SpatialScope, Tangram, gimVI, and SpaGE. The gene expression signatures in the snRNA-seq reference are depicted using a heatmap plot in the sixth column.

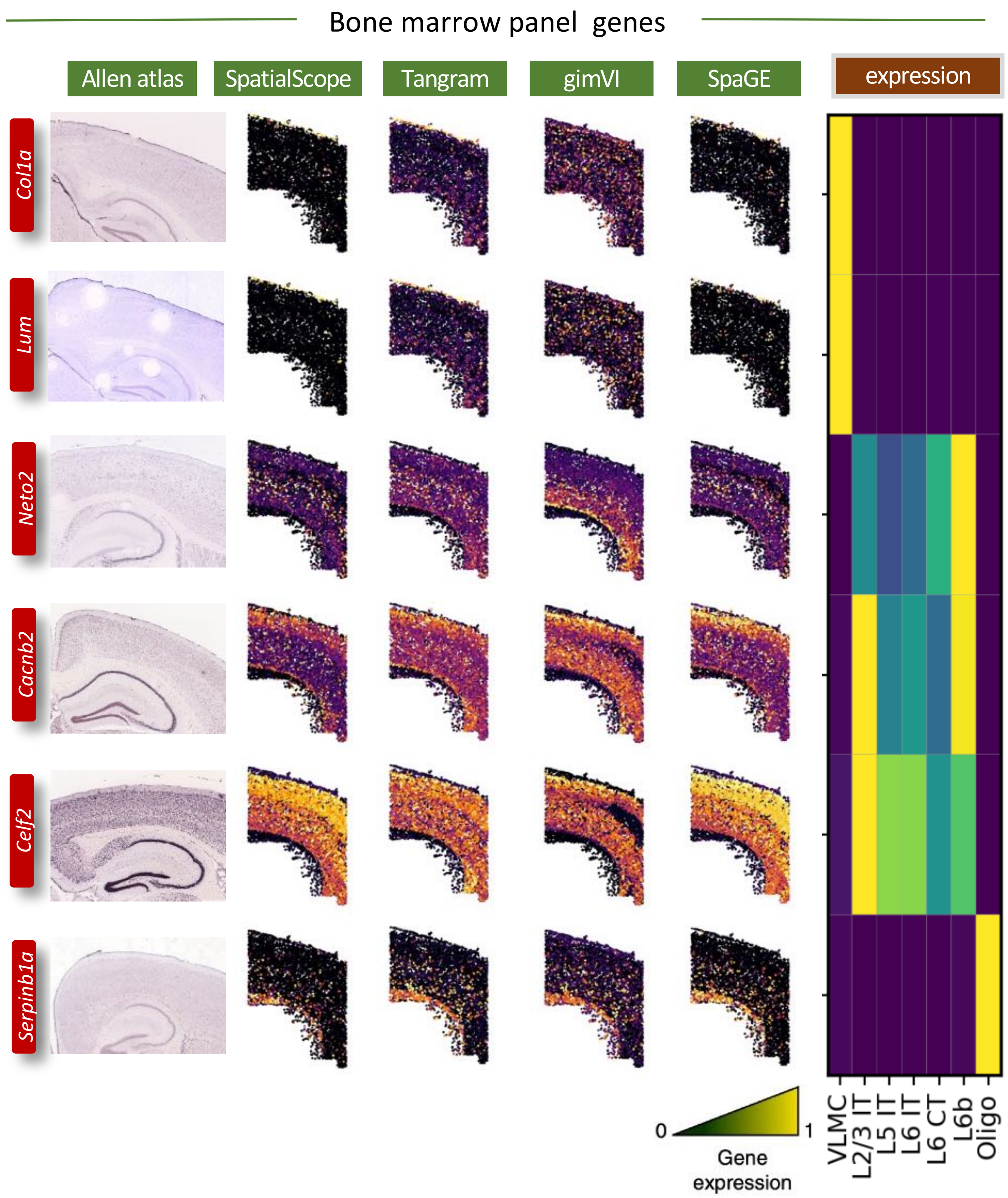

Figure S72: Measured and imputed expressions of non-MERFISH bone marrow panel genes. Each row corresponds to a single gene. The first column from the left shows the ISH images from the Allen Brain Atlas, while the second to fifth columns show the corresponding imputed expression patterns by SpatialScope, Tangram, gimVI, and SpaGE. The gene expression signatures in the snRNA-seq reference are displayed using a heatmap plot in the sixth column.

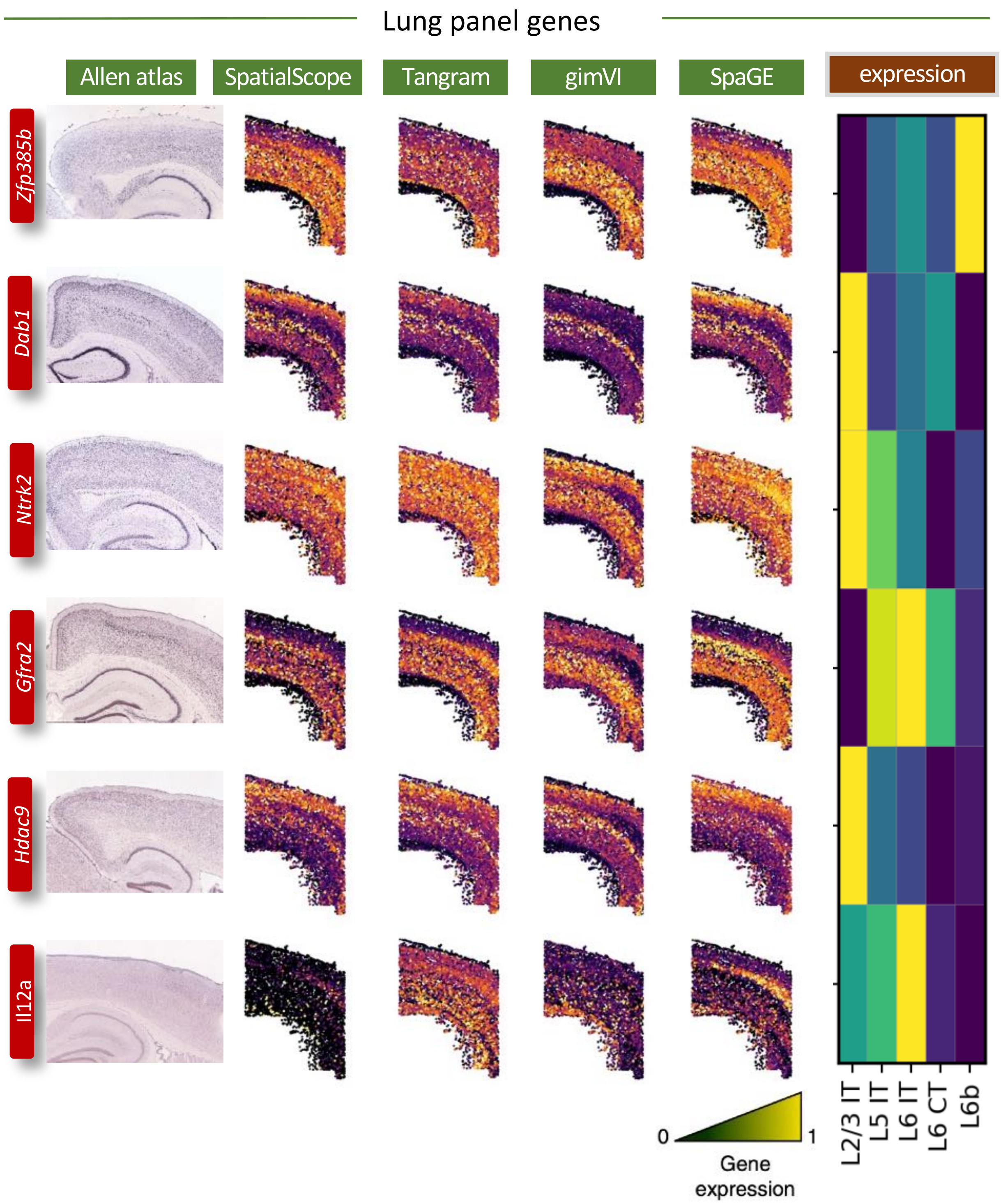

Figure S73: Measured and imputed expressions of non-MERFISH lung panel genes. Each row corresponds to a single gene. The first column from the left shows the ISH images from the Allen Brain Atlas, while the second to fifth columns show the corresponding imputed expression patterns by SpatialScope, Tangram, gimVI, and SpaGE. The gene expression signatures in the snRNA-seq reference are displayed with a heatmap plot in the sixth column.

2 Supplementary Methods

2.1 Nucleus segmentation.

Nucleus segmentation is a long-standing yet important research topic in computer vision for the medical imaging field, and several publicly available tools exist [1, 2, 3, 4]. We considered three most commonly used methods, StarDist (version 0.8.3) [1], Cellpose (version 2.2) [3] and DeepCell [4], and selected the one with better performance as a building block in SpatialScope.

These segmentation methods, namely StarDist, Cellpose, and DeepCell, are designed for different image data and different purposes. StarDist and Cellpose are designed for H&E images for nucleus segmentation, while DeepCell is designed for DAPI images for cell segmentation. When applied to applicable data, all of them can be used to count the number of cells at each spot and thus can serve as the Step 1 building block of SpatialScope. Considering 10x Visium data with H&E images is wildly used and was more discussed for the SpatialScope model, we conduct comprehensive evaluations of the compared methods for segmentation on H&E-stained images. We considered two criteria to evaluate the accuracy and performance of the compared methods.

Evaluation with benchmarking datasets We utilized two benchmarking datasets that provide manually annotated ground truth nuclei: CoNSeP [2] and Kumar [5], which are based on H&E-stained images. The CoNSeP (Counting Nuclei in Synthetic Images) dataset is a publicly available dataset specifically designed for training and evaluating algorithms for nucleus detection and counting in microscopy images. It comprises 41 microscopy images obtained from UHCW, with a total of 24,319 annotated nuclei [2]. This dataset serves as a valuable resource for assessing the accuracy and effectiveness of nucleus segmentation methods. In addition to CoNSeP, we also employed the Kumar nucleus segmentation dataset, also known as the Kumar Dataset 2017. This dataset is widely recognized and utilized in the field of computer vision and image analysis. It consists of 30 microscopy images sourced from TCGA and provides a comprehensive collection of 21,623 annotated nuclei [5]. The Kumar dataset offers a diverse set of challenging images for evaluating the performance of nucleus segmentation methods.

We used two widely used metrics, Dice's coefficient (DICE) and aggregated Jaccard index (AJI), to quantitatively evaluate the segmentation accuracy of the compared methods. These metrics provide objective measures of overlap and agreement between the segmented regions and the ground truth nuclei masks, with higher values indicating better performance. Figure S35 illustrates the results obtained. StarDist exhibited the highest performance, achieving average DICE and AJI scores of 0.85 and 0.68, respectively. Cellpose also demonstrated comparable performance, with average DICE and AJI scores of 0.83 and 0.65, respectively. This aligns with our previous findings in the two 10X Visium H&E stained images, where Cellpose tended to miss more nuclei compared to StarDist (Fig. S37). In contrast, DeepCell's performance was not good, as it was trained solely on DAPI images rather than H&E stained images. Furthermore, the conventional segmentation method, Watershed, exhibited inferior performance in nucleus segmentation for H&E stained images. To validate the robustness of our findings, we conducted similar evaluations using the Kumar benchmarking dataset (Fig. S36). The results were consistent, further affirming the reliability and robustness of StarDist as the best off-the-shelf nucleus segmentation tool for H&E-stained histological images.

Evaluation with 10x Visium real datasets Next, we evaluated the compared methods in two real 10x Visium datasets with H&E-stained histological images (Fig. S37). Since the 10x Visium data lack manually annotated ground truth nuclei, we assessed the accuracy of segmentation results by comparing segmented region and visible nuclei in images through naked eye. In the first 10x human heart data, StarDist located 1,797 single cells while Cellpose only found 1,301 cells. Clearly, Cellpose performed worse as a result of substantial missing cells, especially in the zoom-in region. For the second 10x mouse brain cortex dataset, we observed similar results that StarDist (n=1,563) segments more cells than Cellpose (n=1,250).

In summary, based on our comprehensive analysis, StarDist emerges as the top-performing nucleus segmentation tool for H&E-stained histological images. Its superior performance, as indicated by high DICE and AJI scores, establishes its robustness and reliability in accurately segmenting nuclei in such images. When considering nucleus segmentation on H&E images, we choose StarDist as the building block of SpatialScope for cell counting.

2.2 Cell type identification.

Suppose we have K cell types in single-cell reference data. The expression counts of G genes have been measured to capture the whole transcriptome in the scRNA-seq data. Let $k_{i,m} \in \{1, 2, \ldots, K\}$ be the cell type of the m-th cell at spot i, where $m = 1, \ldots, M_i$. Our goal is to infer the cell type vector $\mathbf{k}_i = \{k_{i,m}\}$ at spot i by integrating scRNA-seq and ST data.

2.2.1 Model setting

In this section, we revisit the probabilistic model for cell type identification in spatial transcriptomics (ST) data by incorporating scRNA-seq reference data. Inspired by RCTD [6], cell type means $\mu_{k,g}$ for cell type $k \in K$ and gene $g \in G$ are first estimated from annotated single-cell reference data:

$$\hat{\mu}_{k,g} \equiv \frac{1}{I_k} \sum_{n=1}^{I_k} \frac{x_{n,k,g}}{N_{n,k}}, \tag{1}$$

where I_k is the number of cells in reference of cell type k, $N_{n,k}$ is the number of UMIs of cell n and cell type k in single-cell reference data, and $x_{n,k,g}$ is observed counts of gene g in this cell.

Next, we build a probabilistic model for each unique molecular identifier (UMI) in spots. For each UMI, the source cell is first probabilistically determined. Second, the gene that the UMI belongs to is determined based on the cell type of that cell. Formally, for each spot $1 \leq i \leq I$ and for each read $1 \leq r \leq N_i$, the probability that the read belongs to cell $1 \leq \theta_{r,i} \leq M_i$ and gene $1 \leq z_{r,i} \leq G$ are:

$$P\left(\theta_{r,i} = m | M_i, \mathbf{k}_i\right) = \frac{1}{M_i}, \quad P\left(z_{r,i} = g | \theta_{r,i}, \gamma, \varepsilon\right) \propto \delta_{i,\theta_{r,i},g}. \tag{2}$$

Here, $\delta_{i,m,g}$ is defined as following

$$\log\left(\delta_{i,m,g}\right) = \alpha_i + \log\left(\hat{\mu}_{k_{i,m},g}\right) + \gamma_g + \varepsilon_{i,g}, \tag{3}$$

where $\varepsilon_{i,g} \sim \mathcal{N}\left(0, \sigma_\varepsilon^2 \mathbf{I}\right)$ is a random effect to account for additional noise, and $\mu_{k,g}$ is defined as Equation (1). Both γ_g and α_i are designed to address the batch effect between single-cell

reference and ST data. More specifically, $\gamma_g \sim \mathcal{N}(0, \sigma_\gamma^2 \mathbf{I})$ represents a gene-specific random effect of accounting for expression differences of a gene g between single-cell and ST platforms, and α_i is the spot-specific effect to account for differences of a gene set across platforms (See section 2.2.2 and section 2.2.3).

By combining formula (2)(3) and integrating θ out, the following holds,

$$\lambda_{i,g} \equiv P\left(z_{r,i} = g | \alpha_i, \gamma_g, \varepsilon_{i,g}, M_i, \mathbf{k}_i\right) \propto \frac{1}{M_i} \sum_{m=1}^{M_i} \hat{\mu}_{k_{i,m},g}. \tag{4}$$

Consider $y_{i,g}$, the observed gene expression counts of gene g at spot i in ST data, as the sum of the reads that belong to gene g in spot i, $y_{i,g} = \sum_{r=1}^{N_i} \mathbb{I}\left(z_{r,i} = g\right)$. Then we have

$$y_{i,1}, y_{i,2}, \ldots, y_{i,G} | \lambda_{i,1}, \lambda_{i,2}, \ldots, \lambda_{i,G} \sim \text{Multinomial}\left(N_i, \lambda_{i,1}, \lambda_{i,2}, \ldots, \lambda_{i,G}\right). \tag{5}$$

Following RCTD, this distribution can be approximated by the Poisson distribution. Assuming that N_i follows a Poisson distribution, $N_i \sim \text{Poisson}(\mu_i)$, and $y_{i,1}, y_{i,2}, \ldots, y_{i,G} \mid N_i \sim \text{Multinomial}\left(N_i, \lambda_{i,1}, \lambda_{i,2}, \ldots, \lambda_{i,G}\right)$. This induces $y_{i,g} \overset{\text{ind}}{\sim} \text{Poisson}\left(\mu_i \cdot \lambda_{i,g}\right)$. We can estimate $\hat{\mu}_i = N_i$ when μ_i is large. In practice, We filter spots with low UMIs and only consider spots with $N_i \geq 100$ to ensure that we are working in the regime of large μ_i. Finally, we can model the counts $y_{i,g}$ as following,

$$\begin{aligned} y_{i,g} | \lambda_{i,g} &\overset{\text{ind}}{\sim} \text{Poisson}\left(N_i \lambda_{i,g}\right), \\ \log\left(\lambda_{i,\mathrm{g}}\right) &= \alpha_i + \log\left(\frac{1}{M_i} \sum_{m=1}^{M_i} \hat{\mu}_{k_{i,m},g}\right) + \gamma_{\mathrm{g}} + \varepsilon_{i,\mathrm{g}}. \end{aligned} \tag{6}$$

2.2.2 Spot-specific effect α_i accounting for platform differences

Parameter α_i is a free parameter that accounts for the difference in the total probability of observing a gene in the gene set $G = \{1, 2, \ldots, G\}$ between scRNA-seq and spot i. For example, highly expressed genes selected in scRNA-seq or snRNA-seq datasets may not be detected or less expressed in spot i. The spot index i in α_i allows us to model this gene-set-level batch effect spot-by-spot, as the various cell type compositions across spatial spots may be associated with the gene-set-level batch effect.

2.2.3 Gene-specific effect γ_g accounting for platform difference

The platform batch effect characterized by the gene expression shift between scRNA-seq and spatial transcriptomics data limits the cell type knowledge transfer between the two RNA sequencing technologies. Therefore, following RCTD, we estimate and then correct the batch effect by summarizing the spatial transcriptomics data as a single pseudo-bulk measurement S_g:

$$S_g \equiv \sum_{i=1}^{I} y_{i,g} \sim \text{Poisson}\left(\sum_{i=1}^{I} N_i \lambda_{i,g}\right). \tag{7}$$

Next we derive $N_i\lambda_{i,g}$. Plug Equation (6) into Equation (7), We have $\forall i, g$,

$$\begin{aligned}\sum_{i=1}^{I} N_i\lambda_{i,g} &= \sum_{i=1}^{I}\sum_{m=1}^{M_i}\frac{1}{M_i}N_i\mu_{k_{i,m},g}e^{\gamma_g+\alpha_i+\varepsilon_{i,g}} = \sum_{i=1}^{I}\sum_{k=1}^{K}N_i\frac{M_i^k}{M_i}\mu_{k,g}e^{\gamma_g+\alpha_i+\varepsilon_{i,g}} \\ &= e^{\gamma_g}\sum_{k=1}^{K}\mu_{k,g}\sum_{i=1}^{I}N_i\frac{M_i^k}{M_i}e^{\alpha_i}e^{\varepsilon_{i,g}} \\ &= Ie^{\gamma_g}\bar{N}\sum_{k=1}^{K}\mu_{k,g}B_{k,g},\end{aligned} \tag{8}$$

where M_i^k is the number cells in spot i that belongs to cell type k and

$$\bar{N} = \frac{1}{I}\sum_{i=1}^{I}N_i, \quad B_{k,g} = \frac{1}{I}\sum_{i=1}^{I}\frac{N_i}{N}\frac{M_i^k}{M_i}\exp\left(\alpha_i+\varepsilon_{i,g}\right). \tag{9}$$

Notice that $B_{k,g}$ only related to g though $\varepsilon_{i,g}$. To get the point estimate of γ_g, we use the mean of $\varepsilon_{i,g}$ to replace ε and define $W_k = \frac{1}{I}\sum_{i=1}^{I}\frac{N_i}{N}\frac{M_i^k}{M_i}e^{\alpha_i}e^{\sigma_\varepsilon^2/2}$ as a new unconstrained parameter representing the average bulk cell type proportion of cell type k. Then we have

$$S_g|\gamma_g \sim \text{ Poisson }\left(I\bar{N}e^{\gamma_g}\sum_{k=1}^{K}\mu_{k,g}W_k\right), \quad \gamma_g \sim \text{Normal}\left(0, \sigma_\gamma^2\right). \tag{10}$$

When I is large, the bulk Poisson mean is large for most genes. Therefore, we can approximate S_g by it's mean:

$$\bar{S}_g \approx e^{\gamma_g}\sum_{k=1}^{K}\mu_{k,g}W_k \Longrightarrow \gamma_g|\hat{W} \approx \log\left(\bar{S}_g\right) - \log\left(\sum_{k=1}^{K}\mu_{k,g}\hat{W}_k\right) \equiv \hat{\gamma}_g, \tag{11}$$

where $\bar{S}_g = S_g/(I\bar{N})$ and W_k is estimated by solving the optimization problem $\hat{W}_k = \arg\min_{W_k}\frac{1}{2}\|\log\left(\bar{S}_g\right) - \sum_{k=1}^{K}\hat{\mu}_{k,g}W_k\|^2$.

2.2.4 Cell type identification

After estimating platform effects, we treat the estimates $\hat{\gamma}_g$ and also $\hat{\mu}_{k,g}$ as fixed and then use SpatialScope to obtain the MAP estimate of cell type label $k_{i,m}$, where $i = 1, 2, \ldots, I$, and $m = 1, 2, \ldots, M_i$. Before the estimation, we first discuss the initialization of $\{k_{i,m}\}$. A warm start of RCTD will be applied to make the model more efficient. The major cell type of each spot estimated by RCTD will serve as the initial cell type for all cells in that spot. Besides, the platform effect α_i will also be estimated in the warm start, and we denote it as $\hat{\alpha}_i$. Next, we apply an iterative algorithm to identify cell type label $\{k_{i,m}\}$ and σ_ε. Recall that the MAP estimate for $\{k_{i,m}\}$ when given σ_ε is as Equation (6) in the main text method, where we update two labels $k_{i,m}, k_{i,\tilde{m}}$ at a time. The prior of $\{k_{i,m}\}$ is given as Equation (4).

To make Equation (6) in the main text feasible, we have the following derivation for $\log p\left(y_{i,g}|\hat{\boldsymbol{\theta}}_c, k_{im}, k_{i\tilde{m}}, k_{-\{(i,m),(i,\tilde{m})\}}\right)$ and $\log p\left(k_{im}, k_{i\tilde{m}}|k_{-\{(i,m),(i,\tilde{m})\}}\right)$. First, we derive

$\log p\left(y_{i,g}|\hat{\boldsymbol{\theta}}_c, k_{im}, k_{i\tilde{m}}, k_{-\{(i,m),(i,\tilde{m})\}}\right)$. Define $w_{k,i} = \frac{1}{M_i} M_i^k e^{\hat{\alpha}_i}$, $\mathbf{w}_i = \{w_{k,i}\}_{k=1}^K$ and

$$\bar{\lambda}_{i,g}(\mathbf{w}_i) = \sum_{k=1}^K w_{k,i}\hat{\mu}_{k,g} e^{\hat{\gamma}_g} = \sum_{k=1}^K w_{k,i}\bar{\mu}_{k,g}. \tag{12}$$

Based on Equation (6), we have following holds:

$$y_{i,g}|\bar{\lambda}_{i,g} \sim \text{Poisson}\left(e^{\varepsilon_{i,g}} N_i \bar{\lambda}_{i,g}(\mathbf{w}_i)\right), \quad \varepsilon_{i,g} \sim \text{Normal}\left(0, \hat{\sigma}_\varepsilon^2\right). \tag{13}$$

By integrating out $\varepsilon_{i,j}$, we derive $\log p\left(y_{i,g}|\hat{\boldsymbol{\theta}}_c, k_{im}, k_{i\tilde{m}}, k_{-\{(i,m),(i,\tilde{m})\}}\right) = \log p\left(y_{i,g}|\bar{\lambda}_{i,g}\right)$ as following,

$$\begin{aligned} p\left(y_{i,g}|\bar{\lambda}_{i,g}\right) &= \int_{-\infty}^{\infty} p_\sigma(z) p\left(y_{i,j}|\lambda_{i,g} = \bar{\lambda}_{i,g} e^z\right) \mathrm{dz} \\ &= \int_{-\infty}^{\infty} p_\sigma(z) e^{-\bar{\lambda}_g N_i e^z} \frac{\left(N_i e^z \bar{\lambda}_g\right)^{y_{i,g}}}{y_{i,g}!} \mathrm{dz} \\ &= Q_{y_{i,g}}\left(\bar{\lambda}_{i,g}\right). \end{aligned} \tag{14}$$

Here, p_σ is the probability density function of ε. When $\hat{\sigma}_\varepsilon$ is obtained, $p_\sigma(z) = p_{\hat{\sigma}_\varepsilon}(z) = \frac{1}{\hat{\sigma}_\varepsilon\sqrt{2\pi}} e^{-\frac{1}{2}\left(\frac{z}{\hat{\sigma}_\varepsilon}\right)^2}$. The probability in Equation (14) is only related to the values of $y_{i,g}$ and $\bar{\lambda}_{i,g}$. To make the algorithm more efficient, a value table of the integration values in Equation (14) with respect to the values of $y_{i,g}$ and $\bar{\lambda}_{i,g}$ will be prepared in advance and $p\left(y_{i,g}|\bar{\lambda}_{i,g}\right)$ is calculated by searching the table in the algorithm.

Next, we derive the prior distribution $\log p\left(k_{im}, k_{i\tilde{m}}|k_{-\{(i,m),(i,\tilde{m})\}}\right)$. Recall that the prior of $\mathbf{K}$ is defined as Equation (4) in the main text. To simplify the notation, omit subscript i and use $k_m, k_{\tilde{m}}$ to denote the cell types of the cells (i,m) and $(i,\tilde{m})$ respectively. We aim to calculate $p\left(k_{im}, k_{i\tilde{m}}|k_{-\{(i,m),(i,\tilde{m})\}}\right) = p(k_m, k_{\tilde{m}})$, where we omit $k_{-\{(i,m),(i,\tilde{m})\}}$ conditioning. To make the notation simple, we also omit $k_{-\{(i,m),(i,\tilde{m})\}}$ in the following derivation but keep in mind that all the probability in the following derivation of $p\left(k_{im}, k_{i\tilde{m}}|k_{-\{(i,m),(i,\tilde{m})\}}\right)$ are conditional on $k_{-\{(i,m),(i,\tilde{m})\}}$. Notice that $p(k_m, k_{\tilde{m}})$ can be rewrite as,

$$p(k_m, k_{\tilde{m}}) = p(k_{\tilde{m}}|k_m)p(k_m). \tag{15}$$

To simplify the notation, denote $v_{m\tilde{m}} = p(k_m|k_{\tilde{m}}), v_{\tilde{m}m} = p(k_{\tilde{m}}|k_m)$. Since $p(k_{\tilde{m}})$ is a probability density function, it satisfies

$$\sum_j \frac{p(k_m)v_{\tilde{m}m}}{v_{m\tilde{m}}} = \sum_j p(k_{\tilde{m}}) = 1. \tag{16}$$

Using this condition, we can rewrite $p(k_m)$ as $\frac{1}{\sum_j \frac{v_{\tilde{m}m}}{v_{m\tilde{m}}}}$. Plug $p(k_m) = \frac{1}{\sum_j \frac{v_{\tilde{m}m}}{v_{m\tilde{m}}}}$ into Equation (15), we have

$$p(k_m, k_{\tilde{m}}) = v_{\tilde{m}m} \cdot p(k_m) = v_{\tilde{m}m} \frac{1}{\sum_j \frac{v_{\tilde{m}m}}{v_{m\tilde{m}}}}. \tag{17}$$

After taking logarithms on both sides of Equation (17):

$$\log p(k_m, k_{\tilde{m}}) = \log v_{\tilde{m}m} - \log \sum_j \frac{v_{\tilde{m}m}}{v_{m\tilde{m}}}. \tag{18}$$

Because both $v_{\tilde{m}m}$ and $v_{m\tilde{m}}$ can be calculated by Equation (4) in the main text, the prior term $\log p\left(k_{im}, k_{i\tilde{m}} | k_{-\{(i,m),(i,\tilde{m})\}}\right)$ in Equation (6) in the main text now becomes feasible by using Equation (18).

Finally, plug Equation (14) and Equation (18) into Equation (6) in the main text and then we can obtain MAP estimate of $k_{i,m}, k_{i,\tilde{m}}$ by maximizing the posterior distribution Equation (6) in the main text . By finding the MAP estimate, we not only use information from gene expression levels $y_{i,g}$ to determine the cell type labels $k_{i,m}$, but also incorporate information from its neighbors.

Algorithm We iteratively perform the following two steps: finding MAP estimate of $\{k_{i,m}\}$ and finding MLE for σ_ε. When finding MAP for $\{k_{i,m}\}$ given σ_ε, we maximize Equation (6) in the main text one cell pair in a spot at a time and then iterate over all cell pairs. For each spot, the five cell types with the largest proportion are selected from the warm start results as candidate cell types for all cell pairs at that spot. When maximizing Equation (6) in the main text , we search for all possible combinations of the two cell types among the five candidate cell types for computational efficiency. The combination that has the largest posterior Equation (6) in the main text will be chosen as the MAP estimate for $k_{i,m}, k_{i,\tilde{m}}$.

Next, we compute MLE for σ_ε. When assume other parameters $\hat{\mu}_{k,g}, \hat{\gamma}_g, \hat{\alpha}_i, \hat{\mathbf{K}}$ are fixed, the log-likelihood of σ_ε is given by Equation (14). The difference is that now we maximize Equation (14) with respect to σ_ε. Inspired by RCTD, we initialize $\sigma_\varepsilon = 1$. For each iteration, we randomly choose 500 spots, and then the MLE estimate of σ_ε is calculated over these 500 spots. We maximize Equation (14) with respect to σ_ε by searching 16 neighbors of previous σ_ε value and the values of Equation (14) are obtained according to the pre-calculated table. The new σ_ε value is chosen with the largest log-likelihood.

"Smoothness" hyper-parameters In cell type identification, spatial information of spatial transcriptomic data is used to determine the labels by incorporating a smoothing prior, as shown in Equation (4) in the main text. This is based on the intuition that cells that are close to each other are more likely to have the same cell type. The "smoothness" hyper-parameters allow us to incorporate spatial information and make our model more robust over the noise. There are two hyper-parameters that are related to "smoothness". One is ν in Equation (4) in the main text, and the other is $\mathcal{N}_{i,m}$, the neighbor for each cell. Both of them are tunable parameters. In practice, 1-norm distance is used to find neighbors. Ten neighbors are assigned to each cell in the default setting. Based on simulation studies, we set $\nu = 10$ to add moderate smoothing. Users can decide to increase both $\mathcal{N}_{i,m}$ and ν, which will result in a smoother spatial distribution of single-cell level cell types.

2.3 Score-based generative models

We briefly review score-based generative models and then show how to leverage a conditional score-based generative model for SpatialScope in section 2.4. Let $\mathbf{x}$ be the log-scale single-cell expression level in single-cell reference data, following distribution $\mathbf{x} \sim p(\mathbf{x})$. The goal of score-based generative modeling is to obtain $p(\mathbf{x})$ by learning the score function: $\nabla_{\mathbf{x}} \log p(\mathbf{x})$ of probability density $p(\mathbf{x})$. Recall that multiple levels of Gaussian noise are added to the data. Let

$\{\sigma_l\}_{l=1}^{L}$ be a sequence of positive noise level that satisfies $\sigma_L > \sigma_{L-1} > \cdots > \sigma_1 \approx 0$, and $\mathbf{x}^{(l)}$ be a sample perturbed by the noise level σ_l^2 with distribution $p_{\sigma_l}\left(\mathbf{x}^{(l)}\right) = \int p(\mathbf{x})\mathcal{N}\left(\mathbf{x}^{(l)}|\mathbf{x}, \sigma_l^2\mathbf{I}\right) d\mathbf{x}$. We aim to train a score network $s_\theta\left(\mathbf{x}^{(l)}, \sigma_l\right)$ to jointly learn all the *scores* of perturbed data distribution $\nabla_{\mathbf{x}^{(l)}} \log p_{\sigma_l}\left(\mathbf{x}^{(l)}\right), \forall l$. Formally, we consider the following objective function, which is called Explicit Score Matching (ESM) [7]:

$$\mathbb{E}_{p(\mathbf{x}^{(l)})}\left[\frac{1}{2}\left\|s_\theta\left(\mathbf{x}^{(l)}, \sigma_l\right) - \nabla_{\mathbf{x}^{(l)}} \log p_{\sigma_l}\left(\mathbf{x}^{(l)}\right)\right\|^2\right]. \tag{19}$$

However, since $\nabla_{\mathbf{x}^{(l)}} \log p_{\sigma_l}\left(\mathbf{x}^{(l)}\right)$ cannot be computed, we consider the following denoising score matching (DSM) objective [7],

$$\ell(\boldsymbol{\theta}; \sigma_l) \triangleq \frac{1}{2}\mathbb{E}_{p(\mathbf{x})}\mathbb{E}_{\mathbf{x}^{(l)}\sim\mathcal{N}\left(\mathbf{x}, \sigma_l^2 I\right)}\left[\left\|\mathbf{s}_{\boldsymbol{\theta}}(\mathbf{x}^{(l)}, \sigma_l) - \nabla_{\mathbf{x}^{(l)}} \log p_{\sigma_l}\left(\mathbf{x}^{(l)}|\mathbf{x}\right)\right\|_2^2\right]. \tag{20}$$

Note that

$$\nabla_{\mathbf{x}^{(l)}} \log p_{\sigma_l}(\mathbf{x}^{(l)}|\mathbf{x}) = -\frac{\mathbf{x}^{(l)} - \mathbf{x}}{\sigma_l^2}. \tag{21}$$

After plugging Equation (21) into Equation (20), the denoising score matching objective Equation (20) is,

$$\ell(\boldsymbol{\theta}; \sigma_l) \triangleq \frac{1}{2}\mathbb{E}_{p(\mathbf{x})}\mathbb{E}_{\mathbf{x}^{(l)}\sim\mathcal{N}\left(\mathbf{x}^{(l)}, \sigma_l^2 I\right)}\left[\left\|\mathbf{s}_{\boldsymbol{\theta}}(\mathbf{x}^{(l)}, \sigma_l) + \frac{\mathbf{x}^{(l)} - \mathbf{x}}{\sigma_l^2}\right\|_2^2\right]. \tag{22}$$

From the above, we can clearly see the intuition of Equation (20). The denoising process is to recover clean data from the data that is corrupted by the noise. The direction in Equation (21) is exactly from the noisy data to the clean data. Also, this is what we learned in the neural network Equation (22).

Then we combine Equation (22) for all noise level $\{\sigma_l\}_{l=1}^{L}$ to get the final objective,

$$\mathcal{L}\left(\boldsymbol{\theta}; \{\sigma_l\}_{l=1}^{L}\right) \triangleq \frac{1}{L}\sum_{l=1}^{L} \lambda\left(\sigma_l\right)\ell\left(\boldsymbol{\theta}; \sigma_l\right). \tag{23}$$

Because we learn all the scores at the same time, we need to add the coefficient $\lambda\left(\sigma_l\right)$ to balance each term and make sure that we learn all the scores successfully. Notice that $\left|\frac{\mathbf{x}^{(l)}-\mathbf{x}}{\sigma_l^2}\right| \propto \frac{1}{\sigma_l}$. To make sure that the outputs of the score network have the same scale for different noise levels, we choose $\lambda_l\left(\sigma_l\right)$ to be σ_l^2.

Then we run annealed Langevin dynamics [8] (see Algorithm 1) to generate new samples from $p(\mathbf{x})$. First, we initialize $\mathbf{x}^{(0)}$ randomly and apply Langevin dynamics with the score network estimated at the largest noise level: $\mathbf{s}_{\boldsymbol{\theta}}(\mathbf{x}, \sigma_L) \approx \nabla_{\mathbf{x}^{(L)}} \log p_{\sigma_L}\left(\mathbf{x}^{(L)}\right)$. Then gradually annealed down the noise level from $l = L$ to $l = 1$ with initialization $\mathbf{x}^{(l,t=1)} = \mathbf{x}^{(l+1,t=T)}$. At the same time, the step size η is also reducing:

$$\mathbf{x}^{(l,t+1)} = \mathbf{x}^{(l,t)} + \eta\mathbf{s}_{\boldsymbol{\theta}}\left(\mathbf{x}^{(l,t)}, \sigma_l\right) + \sqrt{2\eta}\boldsymbol{\varepsilon}^{(l,t)}. \tag{24}$$

Finally, with the noise level and the step size becoming smaller and smaller, we obtain samples from $p_{\sigma_1}(\mathbf{x})$ which is close to the real clean data distribution $p(\mathbf{x})$ when $\sigma_1 \approx 0$.

Algorithm 1 Annealed Langevin dynamics

Require: $\{\sigma_l\}_{l=1}^{L}, \eta_0, T$

Initialize $\mathbf{x}^{(0)}$

for $l = L, L-1, \ldots, 1$ **do**

$\eta = \eta_0 \cdot \sigma_l^2/\sigma_1^2$

for t = 1,2,...,T **do**

Draw $\boldsymbol{\varepsilon}^{(l,t)} \sim \mathcal{N}(\mathbf{0}, \mathbf{I})$,

$$\mathbf{x}^{(l,t+1)} = \mathbf{x}^{(l,t)} + \eta \mathbf{s}_{\boldsymbol{\theta}}(\mathbf{x}^{(l,t)}, \sigma_l) + \sqrt{2\eta}\boldsymbol{\varepsilon}^{(l,t)}. \tag{25}$$

end for

$\mathbf{x}^{(0)} = \mathbf{x}^{(T)}$

end for

2.4 SpatialScope: a conditional score-based generative model for single-cell reference data

Conditional generative models have been long time studied in the generative model field [9, 10, 11, 12, 13]. Many works choose to incorporate the conditioning information into the network for generative models. When the conditional information is discrete variables, one of the challenges is how to match the dimension of the discrete variable and some middle layer of the network, and at the same time, the conditioning information is well incorporated. Discrete conditioning information is always embedded in high dimensional space first, then use strategies like concatenating. For example, the embedding methods include a learnable map [12], or sinusoidal positional encoding [14]. Here, to encode the cell type conditioning information, we propose to learn the score function $\mathbf{s}_{\boldsymbol{\theta}}\left(\mathbf{x}^{(l)}, \sigma_l, \boldsymbol{\mu}_k\right)$ which takes the mean expression level of cell type k as input. The benefits are two-fold. First, $\boldsymbol{\mu}_k$ provides precise information about cell type k. Second, $\boldsymbol{\mu}_k \in \mathbb{R}^G$ has the same dimension of $\mathbf{x}^{(l)}$ such that it will not be ignored. With this key idea, we can design a novel network architecture to learn the score function $\mathbf{s}_{\boldsymbol{\theta}}\left(\mathbf{x}^{(l)}, \sigma_l, \boldsymbol{\mu}_k\right)$ (See network architecture for details). Besides, as empirically noted in [15], $\|\mathbf{s}_{\boldsymbol{\theta}}(\mathbf{x}, \sigma, \boldsymbol{\mu}_k)\| \propto 1/\sigma$ for trained score fuction on real data. We also find that incorporating the noise information by rescaling the score function is more stable and more widely applicable. Therefore, we use noise unconditional score network [16] and inject the information of the noise level by scaling, *i.e.* $\mathbf{s}_{\boldsymbol{\theta}}(\mathbf{x}, \sigma, \boldsymbol{\mu}_k) \approx \mathbf{s}_{\boldsymbol{\theta}}(\mathbf{x}, \boldsymbol{\mu}_k)/\sigma$.

2.5 Network Architectures

The UNet architecture [17] is widely used in image diffusion models and has shown outstanding performance [18, 19]. Here we also use UNet architecture for our conditional score function and found it works well for learning gene expression distribution of single-cell reference data. There are two main differences between our architecture and image diffusion models. First, image data is two dimensional while single-cell data are one dimensional and therefore dilated 1 dimensional convolution is used as the smallest block in our network. Second, as we said before, we use a conditional score function where we take the cell type means $\boldsymbol{\mu}_k, k = 1, 2, \ldots, K$ as

the actual input for score function instead of some embedding of cell type k. More specifically, we add another UNet (Fig. S41) taking $\boldsymbol{\mu}_k$ as input and in the middle of which produces both scale and bias vectors for feature-wise affine transformation applying on feature vector at some middle layer of main UNet that taking $\mathbf{x}$ as input.

The network architecture is shown in Fig. S41. There are two UNets taking $\mathbf{x}$ and $\boldsymbol{\mu}_k$ as input, respectively. In each UNet, three main blocks (MBlock) or conditional blocks (CBlock) are applied to gradually downsample the gene dimension by factors $3, 4, 5$ with the number of channels of $128, 256$, 512, respectively. Then another three MBlocks or CBlocks, which are symmetric to the downsampling process, are applied to gradually upsample the gene dimension. The downsampling process and upsampling process are connected by additional MBlocks or CBlocks without downsampling or upsampling and yield a U-shaped architecture. The MBlock is illustrated in Fig. S40a. Each MBlock includes two residual blocks and four convolutional layers with dilation factors $1, 2, 1, 2$. Each CBlock (Fig. S40b) only includes one residual block and the dilation factors of three convolutional layers inside are $1, 2, 4$. Inspired by [20], the feature-wise linear modulation (FiLM) (Fig. S41, S40c) module is applied to produce feature-wise affine parameters, scale $\mathbf{W}$ and bias $\mathbf{b}$ vectors, to add cell type information in score function. Formally,

$$\mathbf{x}_{\text{mid}} = \mathbf{W}(\boldsymbol{\mu}_k) \odot \mathbf{x}_{\text{mid}} + \mathbf{b}(\boldsymbol{\mu}_k), \tag{26}$$

where $\mathbf{W}$ and $\mathbf{b}$ correspond to the scaling and shift vectors produced by the FiLM module, $\mathbf{x}_{\text{mid}}$ is the corresponding middle layer output from MBlock.

2.6 Hyper-parameters

Here we give the hyperparameters for training the score function and the decomposition process. As suggested by [16], we determine the values of $L, T, \{\sigma_l\}_{l=1}^{L}$, and η_0 as follows. In our default setting, we selected around $2,000$ marker genes. We set $L = 232, T = 5$, and chose $\{\sigma_l\}_{l=1}^{L}$ to be a geometric progression:

$$c = \frac{\sigma_L}{\sigma_{L-1}} = \cdots = \frac{\sigma_2}{\sigma_1} > 1, \tag{27}$$

where c is a constant. Further, σ_L is chosen to be as large as the maximum Euclidean distance between all pairs of different cells from single-cell reference data (See Table S1). We provide detailed hyperparameters setting in Table S1. We use the Adam optimizer [21] for all models with a learning rate 0.0001. Exponential moving average(EMA) is used in training, and the averaged parameters are used when sampling.

We find that the relative order of magnitude between the posterior $\nabla_{\mathbf{X}_i} \log p\left(\mathbf{y}_i|\mathbf{X}_i^{(t)}\right) + \nabla_{\mathbf{X}_i} \log p\left(\mathbf{X}_i^{(t)}|\mathbf{k}_i\right)$ and the injected noise $\sqrt{2\eta}\boldsymbol{\varepsilon}^{(t)}$ will affect the results of decomposition. Therefore, we use a little bit larger η_0 in decomposition (See Table S1) and let $\sigma_{yl} = \sigma_l^{0.5}$ in main text method Algorithm 1.

2.7 Correction of the batch effects between single-cell reference and ST data

Recall that the batch effects between ST and single-cell reference data will hinder gene expression decomposition. To correct the batch effects, we adjust the gene-specific cross-platform effects

Dataset	training dimension	σ_1	σ_L	η_0	η_0 (decomposition)	downsampling channel dimension
Heart	2195	0.002	50	3e-7	1e-6	$128, 256, 512$
MOp	1938	0.01	100	6.6e-6	1e-5	$128, 256, 512$
VISp	2027	0.01	100	6.6e-6	1e-5	$128, 256, 512$
Cerebellum	2394	0.01	100	6.6e-6	1e-5	$128, 256, 512$
Hippocampus	1923	0.002	50	3e-7	1e-6	$128, 256, 512$
MERIFISH	254	0.002	50	3e-7	1e-6	$64, 128, 256$

Table S1: Hyperparameters of SpatialScope for different datasets.

using

$$\mathbf{y}_i = [y_{i,1}/\exp(\hat{\gamma}_1), \ldots, y_{i,G}/\exp(\hat{\gamma}_G)], \tag{28}$$

where $y_{i,g}$ are the observed expression counts of gene g at spot i and $\hat{\gamma}_g$ is the batch effect of gene g estimated under model Equation (6). Next, we account for the difference in sequencing depth by normalizing the total count of $\mathbf{y}_i$ to the mean of the total transcript counts of individual cells from single-cell reference data:

$$\mathbf{y}_i \leftarrow \frac{\mathbf{y}_i}{\sum_g y_{i,g}} \cdot \left(\frac{1}{N_{sc}} \sum_{n=1}^{N_{sc}} \sum_g x_{n,g} \right), \tag{29}$$

where N_{sc} is the total cell number of single-cell reference data and $x_{n,g}$ is the count data of cell n and gene g from single-cell reference data.

2.8 The comparison between SpatialScope and RCTD

We compared SpatialScope over RCTD in terms of method utility, model, algorithm and downstream applications.

From the method utility standpoint, RCTD is a powerful method for cell type deconvolution in spatial transcriptomics (ST) data analysis. It inputs low-resolution ST data (e.g., 10X Visium) and single-cell reference data, and outputs cell-type proportions at each spatial spot. It only offers the average gene expression pattern of a given cell type. For low-resolution ST data, SpatialScope can infer (i) the number of cells, (ii) their corresponding cell types, and (iii) the gene expression of individual cells at each spot. Beyond low-resolution ST data analysis, SpatialScope can also impute gene expression for image-based high-resolution ST data (e.g., MERFISH data). In summary, SpatialScope can provide transcriptome-wide expression levels at single-cell resolution, while RCTD only offers cell type proportions at the spot level. The single-cell resolution ST data produced by SpatialScope not only enables clear visualization of fine-grained cellular gradients, but also enables the detection of spatially resolved cellular communication, revealing meaningful biological processes and inter-cellular dynamics in space.

From the perspective of model design, “Step 3: gene expression decomposition” plays a crucial role in SpatialScope model. This step is essential for obtaining a spatially resolved cellular transcriptomic landscape by effectively integrating ST data and single-cell reference data using deep generative models. In this step, SpatialScope first learns the expression patterns

of different cell types from single-cell reference data as the prior distribution. By combining the prior information with the likelihood term of the observed ST data, SpatialScope then formulates a posterior sampling to perform gene expression decomposition via the Langevin dynamics. "Gene expression decomposition" enables SpatialScope to infer transcriptome-wide expression levels at single-cell resolution. On the other hand, RCTD does not have its model design for the gene expression decomposition.

To achieve the desired performance of gene expression decomposition, a major challenge comes from learning the prior distribution of different cell types from single-cell reference data. While it has been very successful to learn a score function with natural images, it is highly non-trivial to learn the conditional score function with single-cell datasets. Let $\mathbf{s}_{\boldsymbol{\theta}}(\mathbf{x}^{(l)}, \sigma_l, k)$ be the conditional score function, where $\mathbf{x}^{(l)} \in \mathbb{R}^G$ is the log-scale expression levels of G genes at the l-th noise level σ_l, and k is the cell type label. In our experiment, we find that the learning process often tends to largely ignore the cell type information because the neural network naturally focuses on the vector $x^{(l)} \in \mathbb{R}^G$ rather than the scalar k. To successfully incorporate cell type information, we embed cell type information in a vector whose dimension is comparable to $\mathbf{x}^{(l)}$. Therefore, we propose to learn the score function $\mathbf{s}_{\boldsymbol{\theta}}(\mathbf{x}^{(l)}, \sigma_l, \boldsymbol{\mu}_k)$ which takes the mean expression level of cell type k as input. The benefits are twofold. First, $\boldsymbol{\mu}_k$ provides precise information about cell type k. Second, $\boldsymbol{\mu}_k \in \mathbb{R}^G$ has the same dimension as $\mathbf{x}^{(l)}$ so it will not be ignored. With this key idea, we have designed a novel network architecture to learn the conditional score function $\mathbf{s}_{\boldsymbol{\theta}}(\mathbf{x}^{(l)}, \sigma_l, \boldsymbol{\mu}_k)$. Further details regarding the model design and network architecture can be found in Section 2.5.

As SpatialScope provides transcriptome-wide expression levels at single-cell resolution, the downstream analysis enables the detection of cellular communication by identifying ligand-receptor interactions from seq-based ST data, which is not supported by the RCTD results. For image-based ST data (e.g., MERFISH data), SpatialScope accurately imputes expression levels of unmeasured genes and further allows the identification of more spatially differentially expressed genes, gaining biological insights from the downstream analysis.

Overall, SpatialScope offers advancements over RCTD as highlighted above. We also use Table S2 to summarize these key points.

	▼ RCTD	▼ SpatialScope
Method Utility	▼ Seq-based data only<br>► Cell type proportions at each spot<br>► Average gene expression levels of cell types	▼ Seq-based data (e.g., 10 X Visium)<br>► The number of Cells at each spot<br>► Cell type labels of individual cells<br>► Expression levels of individual cell<br>▼ Image-based ST data (e.g., MERFISH)<br>► Imputation of gene expression<br>**In summary, SpatialScope provides transcriptome-wide expression levels at single-cell resolution.**
Model design	Poisson model with single cell reference data	• Deep Generative model (prior distribution learning with reference data)<br>• Langevin dynamics (posterior sampling) with observed spatial transcriptomics data
Architecture and algorithm	• Methods of moments<br>• Likelihood-based method for parameter estimation	A novel deep neural network structure to learn the conditional score function from single-cell reference data
Downstream analysis	Spatially differentially expressed (DE) genes for seq-based ST data	➤ Detection of cellular communication by identifying ligand-receptor interactions from seq-based ST data<br>➤ Spatial DE for seq-based ST data<br>➤ Identification of more spatial DE genes for image-based ST data

Table S2: The comparison between SpatialScope and RCTD.

2.9 Simulation design

2.9.1 Benchmarking datasets

To provide a better comparison between SpatialScope and other related methods, we redesigned our benchmarking study. Following the idea of the benchmarking paper [22], we utilized real MERFISH and STARmap datasets to generate simulation datasets, leveraging their high single-cell resolution capabilities. As the cell type labels and gene expression levels are known at single-cell level, it is straightforward for us to utilize this information to establish the ground truth for evaluation. Our simulated datasets (Fig. S4) in the benchmarking study consist of four single-slice datasets (Dataset 1- Dataset 4) and two multiple-slice datasets (Datasets 5 and 6), as detailed below.

Dataset 1: MERFISH MOp
The MERFISH MOp dataset consists of 254 genes and approximately 300,000 single cells obtained from 64 mouse brain MOp slices belonging to 12 different samples [23]. For our study, we focused on the "mouse1_slice180" from the "mouse1_sample4" and used it to construct a simulation dataset (Figure S4a). The selected slice, "mouse1_slice180", contains 5,551 cells and exhibits a horizontally structured multi-layer pattern. To obtain the single-cell reference data, we vertically partitioned this dataset into two parts. The right part, comprising approximately 4,000 cells, served as the paired single-cell reference data. The left part, containing around 1,000 cells, was used to generate low-resolution ST data by aggregating the cells on uniform grids, creating simulated spots. We generated simulated spots with a grid size of 34×30 μm, resulting in 1-5 cells within each simulated spot. To study the robustness of the compared methods to data quality, we downsampled unique molecular identifier counts (UMIs), specifically using values of 130, 260, and 520, which corresponded to 0.5, 1, and 2 cell UMIs in the raw MERFISH data of this slice.

Dataset 2: MERFISH Mouse brain section 1
The MERFISH Mouse brain section 1 dataset was obtained from the mouse frontal cortex and striatum regions provided by the Allen dataset [24]. This dataset consisted of approximately 0.3 million single cells derived from multiple slices of juvenile and old mice. For our study, we selected tissue slice 0 from donor ID 12 as the MERFISH Mouse brain section 1 dataset. This particular dataset comprised expression values of 374 genes across 17,462 single cells after preprocessing. To simulate ST data and single-cell reference data, we divided these cells into two groups. The first subset consisted of a cropped region (containing 3,489 cells) which was utilized to generate pseudo-spots by aggregating cells within each grid. The remaining cells served as the paired scRNA-seq reference. Using the same simulation pipeline, we generated simulated spots with a grid size of 32×32 μm. resulting in 1-6 cells within each simulated spot (Fig. S4b). In addition, we introduced variability by subsampling UMIs, specifically using values of 53, 107, and 214, which corresponded to 0.5, 1, and 2 cell UMIs in the raw MERFISH frontal cortex and striatum data of this slice.

Dataset 3: MERFISH Mouse brain section 2
The MERFISH Mouse brain section 2 dataset was obtained from the mouse frontal cortex and striatum regions provided by the Allen dataset[24]. This dataset consisted of approximately

0.3 million single cells obtained from multiple slices of juvenile and old mice. For our study, we utilized tissue slice 1 from donor ID 8 as the MERFISH Mouse brain section 2 dataset, which contained expression values of 374 genes across 12,133 single cells after preprocessing. The right bottom region (cell number=1,768) was cropped to make pseudo-spots by aggregating the cells within each grid. Similarly, we applied the same simulation pipeline to generate simulated spots with grid size equal 31×34 μm, leading to 1-5 cells within the simulated spots (Fig. S4c). We varied the subsampled UMIs as 63, 127 and 255, corresponding to 0.5, 1, and 2 cell UMIs in the raw MERFISH frontal cortex and striatum data of this slice.

Dataset 4: MERFISH Mouse brain section 3
The MERFISH Mouse brain section 3 dataset was obtained from the mouse frontal cortex and striatum regions from the Allen dataset [24]. This dataset imaged about 0.3 million single cells from multiple slices of juvenile and old mice. We used the tissue slice 1 from donor ID 12 as MERFISH Mouse brain section 3 dataset, which contains expression values of 374 genes on 15,675 single cells after preprocessing. The top middle region (cell number=2,829) was cropped to make pseudo-spots by aggregating the cells within each grid. Similarly, we applied the same simulation pipeline to generate simulated spots with grid size equal 34×34 μm, leading to 1-7 cells within the simulated spots (Fig. S4d). We varied the subsampled UMIs as 53, 106 and 212, corresponding to 0.5, 1, and 2 cell UMIs in the raw MERFISH frontal cortex and striatum data of this slice.

Dataset 5: STARmap PLUS Hippocampus 3D
The STARmap PLUS Hippocampus 3D dataset was obtained from the mouse cortical and hippocampal regions as provided by Zeng [25]. This dataset encompassed approximately 2,766 genes observed in 72,165 single cells across eight slices from both TauPS2APP and control mice at 8 and 13 months of age. We selected two slices from the control group that exhibited similar cell type distribution patterns and used the recently developed tool, PASTE [26], to compute a pairwise slice alignment between these two slices, which allowed us to construct an aligned 3D ST data (Fig. S4e). The alignment process resulted in a 3D-aligned ST dataset with 9,428 cells in slice 1 and 9,803 cells in slice 2. To generate paired scRNA-seq reference data, we considered cells from an additional slice in the control group. We employed the same simulation pipeline to simulate spots for evaluation and generated simulated spots with a grid size of 33×33 μm. This resulted in simulated 3D aligned spots containing 1-16 cells within each spot.

Dataset 6: MERFISH MOp 3D
The MERFISH MOp dataset was obtained using an image-based spatial transcriptomics (ST) approach with single-cell resolution [23]. This dataset consists of 254 genes and approximately 300,000 single cells located in 64 mouse brain MOp slices derived from 12 different samples. For our analysis, we selected three adjacent slices, namely "mouse1_slice162", "mouse1_slice170", and "mouse1_slice180" from "mouse1_sample4", to construct a 3D aligned ST dataset (Fig. S4f). After preprocessing and cropping, the three slices contained 972, 950, and 1024 cells, respectively. For the single-cell reference data, here we used the same reference dataset as in Dataset 1. We applied the same simulation pipeline to generate simulated spots with a grid size of 36×36 μm. This resulted in simulated 3D aligned spots containing 1-7 cells within each spot (Fig. S4f).

2.9.2 Different utilities between SpatialScope (Cell type identification) and RCTD.

We perform a simulation study to compare SpatialScope (after steps 1 and 2) and RCTD. For SpatialScope (after steps 1 and 2), it outputs the cell types of detected cells at each spot (single-cell resolution). For RCTD, it outputs the cell type proportions at each spot (spot-level resolution). As their outputs have different resolutions, we first visualize their results of Dataset 1, Dataset 2, Dataset 3, and Dataset 4 in Figure S42. As shown in Figure S42, the overall patterns of SpatialScope and RCTD are quite consistent with each other. Their major difference lies in the resolution of their output, where SpatialScope can output the cell type labels for individual cells within the spots and RCTD only outputs the cell type proportions at each spot.

2.9.3 The baseline method "StarDist+RCTD"

The details of designing and implementing the baseline method StarDist+RCTD are described as following.

(i) In the cell type identification task, we used StarDist to detect the cell number in each spot, the same as in Step 1 of SpatialScope. Based on the number of cells detected at each spot, we directly discretized the cell type proportion estimated by RCTD to obtain the distribution of cell type labels for the detected cells (Fig. S43). For example, suppose a spot had 4 cells. If the cell type proportions estimated by RCTD were [0.23, 0.46, 0.21] for cell types A, B, and C, respectively, after discretization ($4 \times [0.23, 0.46, 0.21] \approx [1, 2, 1]$), the "StarDist+RCTD" method output 1 cell, 2 cells, and 1 cell of cell types A, B, and C, respectively, in the given spot. Finally, we randomly assigned the cell type labels to the detected cells at the spot and used it as the final output of the "StarDist+RCTD" method.

(ii) In the gene expression decomposition task, we directly assigned the average expression of cell types learned from single-cell RNA-seq data as the inferred gene expression for each single cell (Fig. S44). Using the same example, suppose that StarDist+RCTD had detected 1 cell, 2 cells, and 1 cell of cell types A, B, and C in the given spot. The inferred gene expression for the cell type A cell was the average expression of cell type A learned from the single-cell reference data. The average expressions of cell types B and C were assigned to the detected locations of cells B and C, respectively. It should be noted that the two cells of type B had the same inferred gene expression level, both being the average expression of cell type B, by StarDist+RCTD.

2.9.4 Leveraging spatial information

To better demonstrate the effectiveness of spatial smoothness constraint imposed by the Potts model, we initially assessed SpatialScope (after steps 1 and 2) and the "StarDist+RCTD" method based on Datasets 1, 2, 3, and 4 (Fig. S4). We varied the smoothness hyperparameter ν from 0 to 1,000 in increments of 5 for the four single-slice datasets. We computed the error rate for the identified single-cell types at each cell location for both SpatialScope and "StarDist+RCTD". The error rates are depicted in Fig. S45. The error rate trends with different ν values remained consistent across the four datasets. When $\nu = 0$, indicating no smoothness constraint, we anticipated that the error rate of SpatialScope would be comparable to that of the "StarDist+RCTD" method. Conversely, for very large ν values (e.g., $\nu = 1,000$), the error

rate increased significantly due to excessive smoothing. By incorporating spatial information within the range of $\nu = 10 \sim 50$, SpatialScope demonstrated substantial improvement in accurately identifying single cell types at each cell location.

We also calculated the Pearson correlation coefficient (PCC) and root-mean-square error (RMSE) of the cell type proportions estimated by SpatialScope and RCTD at the spot level, based on the four simulated datasets. We varied ν to observe the impact of spatial smoothness. The results are shown in Figures S46 and S47. When ν is small, SpatialScope demonstrates higher PCC and lower RMSE for most of the simulated data compared to RCTD and the "StarDist+RCTD" method. This indicates the effectiveness of incorporating spatial information in SpatialScope.

Next, we applied SpatialScope, RCTD, and "StarDist+RCTD" on two multiple-slices ST data: Dataset 5 from STARMAP PLUS Hippocampus, and Dataset 6 from MERFISH MOp (Fig. S4e,f). The visualizations of the generated single-cell resolution cell-type landscapes obtained from different methods are depicted in Fig. S48b,d. It can be observed that the estimates produced by "StarDist+RCTD" exhibit greater heterogeneity, particularly noticeable in Dataset 5 with higher cell density. In contrast, SpatialScope can generate smoother and more accurate results by effectively incorporating spatial information. By varying the parameter ν and quantifying the performance of cell type identification using the error rate metric, the results align with those obtained from the four single-slice data scenarios (Fig. S49). Notably, when the smoothness constraint is appropriately incorporated (with a small ν value), SpatialScope demonstrates improved performance in terms of cell type identification, surpassing the performance of StarDist+RCTD. Moreover, the integration of multiple slices in SpatialScope leads to enhanced accuracy, as it effectively leverages the information available in neighboring slices. Additionally, when evaluating the spot-level performance of SpatialScope compared to RCTD using metrics PCC and RMSE, similar trends are observed as those observed in the error rate computations (Fig. S50, S51). This further supports the efficacy of the spatial smoothness constraint in the SpatialScope model and its advantages over RCTD.

2.9.5 Missing cell types in single-cell reference

We performed additional simulations to evaluate the impact of missing cell types in reference. Specifically, following the benchmarking analysis of Dataset 1 in the main text, we removed the L6b and Lamp5 cells from the single-cell reference. Then we evaluated the effect of missing cell type on the cell type identification for SpatialScope and the compared methods. Overall, SpatialScope achieved the highest robustness over the compared methods by predicting the cells as the most transcriptionally similar cell type in the reference when the ground truth cell types were missing (Fig. S52). For example, most L6b cells were predicted to be L6 CT cells by SpatialScope as L6 CT is the closest cell type for L6b, and most Lamp5 cells were still classified as GABAergic neurons: Pvalb or Vip. In contrast, substantial L6b and Lamp5 cells were classified as L6 IT Car3 and L4/5 IT by Tangram, respectively. CytoSPACE unreasonably misclassified most Lamp5 cells as Endo, which is a non-neuronal cell type.

2.9.6 Inconsistent cell number in gene expression decomposition task

Although deep nucleus segmentation tools have shown great success in identifying cells in microscopy images, the segmentation result is not 100 percent accurate. For example, some cells were missing due to the weak signal, and some noise pixels may be misidentified as cells. Therefore, it is necessary to evaluate the performance of SpatialScope and the compared method when the ground truth cell number is inconsistent with the estimated cell number in the spots. Following the benchmarking analysis of Dataset 1 in the main text, we created additional simulations in the presence of inconsistent cell numbers. Specifically, based on the original ST data (the second part of the partitioned MERFISH MOp benchmarking dataset, Fig. S53a), we randomly selected some cells as missing cells whose gene expressions still contribute to the simulated spot-level expression profile, but they are not observed in the new ST data. The remaining cells existing in the original ST data (denoted as existing cells) are still observed and contribute to the spot-level gene expressions. Besides, we further randomly added some cells as mis-added cells in this new ST data to mimic the misidentified cells due to the noise pixels. Those mis-added cells have no gene expressions and thus cannot contribute to the spot-level expression profiles, but they are observed in the new ST data (Fig. S53b). Next, following the same analysis pipeline as in the main text, we aggregated all observed cells on uniform grids to generate simulated spots with the grid size: 34×30(μm) and varied subsampled UMIs ranging from 130 to 520. Then we used the paired single-cell reference (the first part of the partitioned MERFISH MOp benchmarking dataset) to perform cell type identification and gene expression decomposition. Of note, we only used the gene expression profiles for the existing cells within the simulated spots as ground truth because the missing cells are not observed, and the mis-added cells have no ground truth.

Fig. S53c shows the overall gene expression decomposition results for this new ST data when considering the spots with missing or mis-added cells. Clearly, SpatialScope achieved significantly higher accuracy of decomposition in all settings. The mean cosine similarity between the ground truth and predicted single-cell level gene expressions by SpatialScope is as high as 0.886 when the subsample UMIs count is 260 and the cell type labels are correctly identified, while the alignment-based methods, Tangram and CytoSPACE, only achieved 0.747 and 0.791 mean cosine similarities, respectively. As some concrete examples of spots with missing cells (Fig. S54), spot 108 contains three cells from L6 CT, Lamp5 and SMC, but the SMC cell was missing. SpatialScope successfully identified the remaining two ground truth cells with the highly matched transcriptional profiles, while the compared methods failed in both cell type identification and gene expression decomposition; Spot 54 contains three cells from L4/5 IT, Endo and L5 ET, but the L5 ET cell was missing. Although SpatialScope misidentified the L4/5 IT cell as L5 IT, the cosine similarity between the predicted and ground truth gene expression is still as high as 0.94 for L4/5 IT cell. This is because L4/5 IT and L5 IT are transcriptionally similar cell types in the reference and the L4/5 IT cell in this spot is very close to L5 IT cluster in the UMAP plot, which leads to the misidentified L5 IT cell by SpatialScope. Nevertheless, with the gene expression distribution approximated by the score-based generative model, SpatialScope can still find the best-matched transcriptional profile as much as possible even though the cell type label is misidentified. For spots with mis-added cells, we also provided four examples in Fig. S55. Spot 463 contains four existing

cells from L4/5 IT and Endo, and one mis-added cell. SpatialScope successfully identified three of the four ground truth cells with a mean cosine similarity of 0.82, and the mis-added cell was identified as the major cell type in this spot: L4/5 IT. Spot 8 contains three existing cells from L4/5 IT and L2/3 IT, and two mis-added cells. SpatialScope successfully identified two of the three ground truth cells with a mean cosine similarity of 0.89, and the two mis-added cells were also identified as the major cell type in this spot.

2.9.7 Hyperparameters sensitivity analysis for training the score-based generative model

One of the unique features and strengths of SpatialScope lies in its utilization of a score-based generative model to accurately approximate the distribution of gene expressions from the scRNA-seq reference data. Then SpatialScope ran the Langevin dynamics to perform posterior sampling for gene expression decomposition at each spot. This unique approach sets SpatialScope apart from other methods and contributes to its superiority. By systematically assessing the hyperparameters in "Step 3: Gene expression decomposition", we can gain insights into SpatialScope's stability and robustness across various datasets and experimental conditions.

We listed several key hyperparameters we tested in our model, including the hyperparameters *epoch*, L, T and σ_{yl}. These hyperparameters cover a wide range and are crucial for understanding the behavior and performance of our model. To facilitate understanding of the experimental results and our model, we provide intuitive explanations for each hyperparameter's role. This will help readers grasp the significance of the hyperparameters and interpret the experimental outcomes effectively. It is important to note that while we focused on evaluating these specific hyperparameters, other hyperparameters in the original score-based generative model have been extensively discussed in the original paper [16]. Therefore, we have omitted those hyperparameters in our evaluation as they have already been addressed in the literature. Throughout our experiments, we utilize Dataset 1 in the benchmarking study. When testing each hyperparameter, we maintain the other hyperparameters at their default values in SpatialScope. This ensures a consistent and fair comparison across different hyperparameter settings.

Hyperparameters *epoch*

We first investigate the performance of SpatialScope under score-based generative models with different training epochs. Precisely, we followed the benchmarking analysis of Dataset 1 in the main text to evaluate the gene expression decomposition performance but varied the training epochs of the score-based generative model saved at 500, 1,500, 7,500, 12,500, and 25,000 epochs. Among them, 7,500 epoch was used in the simulation analysis of the main text. To fairly compare the performance of models with different training epochs, we only considered cells with the correct estimated cell type label. In the simple case when the simulated spots only contain one cell (Fig. S56a), increasing the number of epochs improves the performance significantly when the subsample UMIs count is low. This result suggests that the score-based generative model can better approximate the gene expression distribution with the increase in the number of epochs. As expected, when considering the spots with cell numbers larger than one (Fig. S56b), the gene expression decomposition performance improved steadily as the number of epochs increased for all settings. These results indicate that the score-based

generative model can benefit from increasing the training epochs in most cases. However, due to the trade-off between the performance and the time cost, we recommend that the number of epochs ranges from 5,000 to 10,000, as the improvement after the 10,000 epoch is minor.

Hyperparameters L and T

Intuitively, the score-based generative model learns data distribution by perturbing data with various levels of noise. In the training process, the perturbed data distributions with noise level σ_l is given as $p_{\sigma_l}\left(\mathbf{x}^{(l)}\right) = \int p(\mathbf{x})\mathcal{N}\left(\mathbf{x}^{(l)}|\mathbf{x}, \sigma_l^2\mathbf{I}\right)d\mathbf{x}$, where $\mathbf{x}^{(l)}$ represents a sample perturbed by the noise level $\sigma_l^2, \sigma_L > \sigma_{L-1} > \cdots > \sigma_1 \approx 0$. In this process, we use the network $\mathbf{s}_{\boldsymbol{\theta}}\left(\mathbf{x}^{(l,t)}, \sigma_l\right)$ to train the score of perturbed data distribution $\nabla_{\mathbf{x}^{(l)}} \log p_{\sigma_l}\left(\mathbf{x}^{(l)}|\mathbf{x}\right)$. In the sampling process, Langevin dynamics is used to take samples from the learned data distribution. From $l = L$ to $l = 1$ we run:

$$\mathbf{x}^{(l,t+1)} = \mathbf{x}^{(l,t)} + \eta \mathbf{s}_{\boldsymbol{\theta}}\left(\mathbf{x}^{(l,t)}, \sigma_l\right) + \sqrt{2\eta}\boldsymbol{\varepsilon}^{(l,t)}, \tag{30}$$

where $\boldsymbol{\varepsilon}^{(l,t)} \sim \mathcal{N}(\mathbf{0}, \mathbf{I})$. For each noise level σ_l, we obtain samples $\mathbf{x}^{(l,t)}$ approximately follow the perturbed data distribution $p_{\sigma_l}\left(\mathbf{x}^{(l)}\right)$. This process can also be called the denoising process because the noise level is progressively reduced, and perturbed data distribution gradually approximates target data distribution.

From above that L represents how many noise levels we set, and T is the number of steps when computing (Fig. 30) at a specific noise level. Intuitively, the more extensive the grid of noise levels $\{\sigma_l\}_{l=1}^{L}$, the better for learning (i.e., the larger L, the better). For the sampling step T, similarly, the larger T, the better. However, the larger L and T mean more expensive computational resources. There is a trade-off between the performance and computational cost.

We used Dataset 1 in "Benchmarking datasets" to test the effect of L and T on model performance. We test $L = 10, 40, 80, 150, 232, 500$ and $T = 1, 3, 5, 10$ and evaluate SpatialScope's performance on gene expression decomposition. The cosine similarities between the ground truth and decomposed single-cell level gene expression profiles under different L and T are calculated and compared (Fig. S57). We can see that SpatialScope works very well in a wide range of parameter settings. Therefore, we suggested the default setting of SpatialScope as $L = 232, T = 5$ according to the dimension of single-cell gene expression profiles.

Hyperparameters σ_{yl}

The hyperparameter σ_{yl} shows in Algorithm 1 in the main text, and it is related to the the distribution we assign to the count-scale spot level gene expression profile $\mathbf{y}|\mathbf{x}_1, \mathbf{x}_2, \ldots, \mathbf{x}_M$, where $\mathbf{x}_m, m = 1, 2, \ldots, M$ represents the true count-scale gene expression levels of cells in the spot, and M is the number of cells in that spot. We assign Gaussian distribution to $\mathbf{y}|\mathbf{x}_1, \mathbf{x}_2, \ldots, \mathbf{x}_M \sim \mathcal{N}\left(\sum_{m=1}^{M}\mathbf{x}_m, \sigma_{yl}^2\right)$. The hyperparameter σ_{yl}^2 is the variance of $\mathbf{y}$ corresponding to the perturbed data distribution at the noise level σ_l. In the sampling process, σ_{yl} decreases as σ_l decreases. Formally, we set $\sigma_{yl} = \sigma_l^{\frac{power}{2}}$. We evaluate the gene expression decomposition accuracy under $power = 0.5, 0.8, 1.0, 1.3, 1.5, 1.8, 2.0, 2.3$, and 2.5 (Fig. S58). The performance is quite stable when $power < 2$ and gets the best performance around 1.0. In the default setting of SpatialScope, we set $power = 1$ for all real data analysis.

2.9.8 The comparison of conditional and unconditional score function

As illustrated in Section 2.3 (Gene expression decomposition), we learned conditional score-based generative models from single-cell reference data by additionally embedding the cell type mean gene expression as inputs, which increases the flexibility of our model to accommodate the heterogeneity across cell types. More specifically, the unconditional network requires learning the gene expression distribution of all cell types with one shared network. By contrast, embedding the cell-type information in the conditional network allows capturing cell-type specific information and retaining modeling flexibility. To see this, we trained an unconditional score-based generative model using the paired single-cell reference from the simulation dataset and compared the results with those of the corresponding conditional network. Unsurprisingly, the cells sampled from the distribution learned by the conditional network (Fig. S59a) gradually overlapped with the original single-cell reference data with training epoch increases, while cells generated by the unconditional network continuously showed strong evidence of deviation from the reference, especially for L4/5 IT and L5 IT cell types (Fig. S59b). We further applied the kBET to quantitatively assess how well the single-cell reference and generated cells mixed. kBET hypothesized that the proportions of the batch labels in any neighborhood do not differ from the global distribution in the absence of batch-effect, and it used the rejection rates to quantify the degree of mixing: low rejection rates imply well-mixed datasets, a low rejection rate of 0.2 roughly corresponds to 1% of biased genes (mean gene expression was varied) between the two datasets while a high rejection rate of 0.8 corresponds to 20% of biased genes. [27]. We observed that the kBET rejection rates decreased from 0.771 to 0.296 as the number of epochs increased from 500 to 7500 for the conditional network, while kBET rejection rates of the unconditional network only reduced a little (from 0.809 to 0.759).

Next, we used benchmarking Dataset 1 to evaluate the gene expression decomposition performance between conditional and unconditional networks. We used the checkpoints saved in 7500 epochs for both networks. As shown in Fig. S59c, in the naïve case when the simulated spots only contain one cell, the conditional network is slightly better than the unconditional network when the subsampling UMIs count is as low as 130, and two kinds of networks are comparable when subsampling UMIs larger than 130. However, when considering the spots with cell number larger than one (Fig. S59d), the conditional network shows better performance over the unconditional network in all settings, indicating that the conditional network can improve the gene expression decomposition by learning the gene expression distribution of different cell types in some sense specifically.

2.9.9 Unbalanced cell types in single-cell reference data

Due to the large variation in the proportions of different cell types, we created additional simulations to compare the performance of gene expression decomposition in different cell types using the benchmarking Dataset 1. In the simulated single-cell reference data, L4/5 IT and Vip have the highest abundance (12.5%) and lowest abundance (0.5%), respectively. We first trained a neural network to approximate the conditional score function, such that we learned the distribution of expression patterns of different cell types based on the single-cell reference data. Then we ran the Langevin dynamics to perform posterior sampling for gene expression decomposition at each spot. We used the posterior means as the inferred expression levels of

individual cells. After that, we calculated the cosine similarity between the inferred expression levels and the observed expression to quantify the accuracy of gene expression decomposition.

As shown in Figure S60a, we can see that the accuracy of gene expression decomposition is not largely affected by the cell type abundance. For example, the decomposition accuracies of two cell types L4/5 IT (highest abundance, 12.5%) and Vip (lowest abundance 0.5%) are more or less the same. This indicates that our conditional score function can be well-trained even with a relatively small number of cells. However, we observe that the decomposition accuracy is more related to the heterogeneity of a cell type. Here the heterogeneity of a cell type is measured by the average cosine similarity of expression levels between two randomly chosen cells from the given cell types, using the single-cell reference data. The higher the cosine similarity, the lower the heterogeneity of the given cell type. Figure S60b shows that the decomposition result is more accurate when the expression levels are similar within a cell type. The linear regression results (Fig. S60c) also verify this point, where R square is only 0.11 when regressing the decomposition accuracy to cell type proportion, and R square becomes 0.71 when regressing the decomposition accuracy on cell type heterogeneity. This indicates the decomposition accuracy is more related to the heterogeneity within a cell type rather than cell type proportion in the single-cell reference data.

2.9.10 Unbalanced cell numbers within the spots

To evaluate the robustness of SpatialScope in handling unbalanced cell numbers within the spots, we quantitatively measured the performance of gene expression decomposition by separately evaluating spots with different cell numbers and compared SpatialScope with Tangram and CytoSPACE. We use Dataset 1 (Fig. S4) to illustrate cell numbers' effect on inferring each single cell's gene expression levels. We examined two simulation settings, where either the paired single-cell reference data (produced from the same sample as the ST data, in this case, the right part of Figure S1) or the unpaired single-cell reference data (independently generated scRNA-seq reference of the same tissue type, in this case, an external mouse Primary visual (VISp) scRNA-seq data [28]) are used for gene expression decomposition.

We first explored the setting where we used paired single-cell reference data for gene expression decomposition. As shown in Figure S61, the performance of all compared methods (measured by cosine similarity) degrades as the cell number increases. This is because more cells in a spot means more components introduce more uncertainty in the decomposition. Nevertheless, it is important to note that our method SpatialScope achieved the best performance in different scenarios of cell numbers and UMI subsample rates in the construction of simulated spots.

When an independently generated scRNA-seq reference of the same tissue type was used (Fig. S62), we observed consistent patterns regarding different cell numbers within spots, and SpatialScope outperformed other methods in all settings. As a comparison, the performances of alignment-based methods, Tangram and CytoSPACE, decrease dramatically when the reference data is unpaired (batch effects exist across platforms).

2.9.11 Performance under different grid sizes

To further explore the performance of SpatialScope in various scenarios, we systematically varied the grid size to manipulate the number of cells within each spot (Fig. S3a,c) and compared the performance with RCTD using a single-slice dataset and a multiple-slice dataset. The first dataset is a single-slice MERFISH dataset (Fig. S3a) and is obtained from the mouse frontal cortex and striatum regions provided by the Allen dataset [24], which is Dataset 3 in benchmarking datasets. After preprocessing, it included expression values from 12,133 single cells and 374 genes. The second dataset was derived from multiple slices of the mouse cortex and hippocampus regions, as provided by Zeng [25] (Fig. S3c). This dataset is Dataset 5 included in our benchmarking datasets. The aligned 3D ST data encompassed 19,231 cells and 2,766 genes. Performance was assessed by computing the error rate at the single-cell level and the Pearson correlation coefficient (PCC) at the spot level for cell type identification task and cosine similarity at single-cell lever for gene expression decomposition task. The grid size varies from 50×50 µm to 10×10 µm. The largest size 50×50 µm, emulates the spot size of the Visium spatial transcriptomic technology, allowing for the presence of one to dozens of cells within a spot. The smallest size 10×10 µm, emulates the spot size of the Slide-seq spatial transcriptomic technology, allowing for the presence of one to two cells within a spot. It is worth noting that the error rate of the cell type identification in Step 2 and the accuracy of gene expression decomposition in Step 3 are the key focuses of our method. These metrics reflect how the model performs at the single-cell resolution, which is the primary goal of our method - to overcome the limitations and deficiencies of existing spatial transcriptomics technologies in terms of resolution.

Based on the error rate metric, our method exhibits a distinct superiority over RCTD across almost all grid sizes (Fig. S3b,d left), particularly under large grid sizes, as it effectively exploits the advantages of spatial information. This advantage is further amplified in the multiple-slice dataset due to borrowing information from the adjacent slice (Fig. S3d left). For the inference of single-cell gene expression, SpatialScope exhibits a substantial advantage over RCTD (Fig. S3b,d right), with the magnitude of this advantage becoming more pronounced as the grid size decreases. This evidence demonstrates the major improvement of SpatialScope over RCTD at the single-cell resolution. Regarding cell type deconvolution accuracy at the spot level, SpatialScope achieves comparable performance to RCTD based on the PCC metric in the single-slice dataset (Fig. S3b middle). However, SpatialScope can outperform RCTD in the multiple-slice dataset (Fig. S3d middle) because spatial smoothness offers more advantages in the presence of multiple slices with higher cell density in the data.

In summary, by exploring different scenarios, we can obtain a more comprehensive understanding of the performance and behavior of the SpatialScope and RCTD methods across various settings.

2.9.12 The impact of abundance and variability for gene expression imputation

We investigated the imputation performance of SpatialScope for genes with different abundance and variability. Specifically, we used benchmarking Dataset 1, where the expression profiles for 254 genes were measured in 5,551 single cells in a mouse brain slice from the primary motor cortex (MOp) [23] and droplet-based snRNA-seq profiles from mouse MOp as the reference

dataset [29], in the benchmarking study. We compared SpatialScope with other imputation methods such as Tangram, gimVI, and SpaGE.

We categorized genes into low, medium, and high abundant groups based on their expression levels. Additionally, we categorized genes into low, medium, and high variable groups using the p-values obtained from spatial differential expression analysis. Specifically, genes with expression levels falling within the $0 \sim 10$, $45 \sim 55$, and $90 \sim 100$ quantiles of all gene expression levels were assigned to the low, medium, and high abundant groups, respectively. Moreover, genes with p-values falling within the $0 \sim 10$, $45 \sim 55$, and $90 \sim 100$ quantiles of all gene p-values generated by SPARK-X [30] were selected as high, medium, and low variable genes, respectively.

We randomly selected 25 genes from each group to assess the imputation performance for testing purposes. These genes were excluded from the training set, while the remaining genes were used as training genes. The expression profiles of the 25 testing genes measured in the dataset were considered as the ground truth for evaluation. We utilized a metric called relative MAE to quantify the imputation performance, which is defined as follows:

$$\text{Relative MAE} = \frac{\sum_g \left\| x_g^{\text{Impute}} - x_g^{\text{GT}} \right\|}{\sum_g x_g^{\text{GT}}}, \tag{31}$$

where x_g^{Impute} represents the imputed value of gene g, and x_g^{GT} denotes the ground truth value of gene g. Both x_g^{Impute} and x_g^{GT} are normalized from the count scale to the range $(0, 1)$. The relative MAE reflects that less abundant genes are more challenging to predict, and the performance of all methods deteriorates as the gene's abundance level decreases (Fig. S63a).

Slightly different from expectation, the performance of all methods becomes better when the gene expression level is highly variable (Fig. S63b). This is because if the gene is more variable, it tends to show a specific spatial pattern, or it's more likely a marker gene of a cell type (Fig. S64). With a more obvious spatial pattern for the gene, capturing the pattern from the data for all methods is easier. For example, for SpatialScope, it's easy to learn the pattern of cell type marker genes from single cell reference data. On the contrary, the genes with less abundant or less variable are hard to see spatial patterns (Fig. S64) or even opposite to the pattern in single cell reference data (e.g., gene *Adam2* in "low abundant genes" in Figure S64). The low measurement quality and randomness degrade the performance of all methods. For example, SpatialScope predicts the expressions of gene *Adam2* mainly in the upper layer, which is consistent with the gene expression signatures in snRNA-seq reference (Fig. S64b). However, the expression of gene *Adam2* in MERFISH data is more random, and it expresses in both upper and lower layers, contrary to the expression pattern in snRNA-seq reference. This also explains SpatialScope's results for "Low Abundant Genes" (Fig. S63, red arrow).

Overall, SpatialScope outperformed other methods in almost all gene groups (Fig. S63). For low abundant or low variable genes, whose expression levels measured in the MERFISH data are very low and have little spatial pattern, SpatialScope can still predict expressions that are consistent with the gene expression signatures in snRNA-seq reference. For example, the measured gene *B4galnt3* that belongs to low variable genes shows little spatial pattern (Fig. S64e, the first column). However, SpatialScope predicts its relatively high expression in L6 CT and L6b layer (Fig. S64e, the second column), which is in accordance with the signatures in snRNA-seq reference (Fig. S64e, the last column). Other methods overestimate its expression

in the upper layer. For medium and high abundant genes or medium and high variable genes, the spatial expression pattern of genes becomes more obvious. For these genes, SpatialScope can accurately predict genes' spatial patterns, which are consistent with the measured spatial patterns and the signatures in the snRNA-seq reference. Tangram sometimes overestimates the expression or gives the wrong spatial pattern, like gene *Syndig1* (Fig. S64d) or gene *Brinp3* (Fig. S64g). Methods gimVI and SpaGE missing some spatial patterns in the prediction. For example, gimVI misses the high expression of gene *Lsp1* (Fig. S64f) in L6b and SpaGE misses overestimates the expression of gene *Slc30a3* (Fig. S64g) in L6 CT and L6b layer. Overall, the metric Relative MAE for evaluating performance illustrates the high prediction accuracy of SpatialScope.

2.9.13 The generalization ability of gene expression imputation for gene panels from diverse tissues

We investigated the SpatialScope's imputation performance of genes not present in the MERFISH data. We utilized a publicly available marker gene database called CellMarker 2.0 [31]. This database offers a manually curated collection of experimentally supported markers for various cell types in different human and mouse tissues. It encompasses a total of 35,197 tissue-cell type-marker triplets and 9,616 unique markers. To focus specifically on non-brain tissue markers, we excluded all markers related to brain tissue or those present in the MERFISH data, resulting in a selection of 4,806 unique markers for further analysis. Subsequently, we identified three non-brain tissues with the highest number of unique markers and evaluated the imputation accuracy of their respective gene panels. Since ground truth data for these non-MERFISH genes is unavailable, we utilized the Allen ISH dataset [32] for validation purposes. The Allen ISH dataset provides a valuable resource for assessing the accuracy of gene expression patterns, allowing us to validate the imputation performance of the non-brain tissue gene panels. By leveraging the Allen ISH dataset as a validation set, we can assess the imputation accuracy of the non-MERFISH genes and compare the imputed expression patterns with the actual gene expression patterns observed in the Allen ISH dataset. This approach enables us to evaluate the reliability and effectiveness of the imputation process for non-brain tissue markers.

Figure S71 displays the imputation performance of the compared methods for the gene panel in kidney tissue. As an example, we examined the marker gene *Pde1a*, which is associated with duct intercalated cells in the kidney. The Allen ISH dataset reveals that *Pde1a* tends to exhibit higher expression in the bottom cortical layers of the MOp region, consistent with the imputation results obtained by SpatialScope. Conversely, other imputation methods tend to overestimate the spatial expression of *Pde1a* in the upper cortical layers (Fig. S71, first row). Similarly, we considered the marker gene *Ntrk3*, associated with podocyte cells in the kidney. SpatialScope is the only method capable of successfully recovering the observed spatial expression pattern in the Allen ISH dataset, where higher expression is observed in the L2/3 IT layer and lower expression in other layers (Fig. S71, third row). To demonstrate the robustness of our method, we further evaluated the imputation performance of gene panels from bone marrow (Fig. S72) and lung (Fig. S73) tissues. For instance, both *Col1a* and *Lum* show expression solely in the top cortical layer of the MOp region in the Allen ISH dataset (Fig.

S72), consistent with the imputation results generated by SpatialScope. In summary, these findings provide evidence supporting the ability of SpatialScope to reasonably impute non-brain tissue gene panels, even when using a limited number of markers designed for brain tissue.

References

[1] Uwe Schmidt, Martin Weigert, Coleman Broaddus, and Gene Myers. Cell detection with star-convex polygons. In International Conference on Medical Image Computing and Computer-Assisted Intervention, pages 265–273. Springer, 2018.

[2] Simon Graham, Quoc Dang Vu, Shan E Ahmed Raza, Ayesha Azam, Yee Wah Tsang, Jin Tae Kwak, and Nasir Rajpoot. Hover-net: Simultaneous segmentation and classification of nuclei in multi-tissue histology images. Medical Image Analysis, 58:101563, 2019.

[3] Carsen Stringer, Tim Wang, Michalis Michaelos, and Marius Pachitariu. Cellpose: a generalist algorithm for cellular segmentation. Nature methods, 18(1):100–106, 2021.

[4] Dylan Bannon, Erick Moen, Morgan Schwartz, Enrico Borba, Takamasa Kudo, Noah Greenwald, Vibha Vijayakumar, Brian Chang, Edward Pao, Erik Osterman, et al. Deepcell kiosk: scaling deep learning–enabled cellular image analysis with kubernetes. Nature methods, 18(1):43–45, 2021.

[5] Neeraj Kumar, Ruchika Verma, Sanuj Sharma, Surabhi Bhargava, Abhishek Vahadane, and Amit Sethi. A dataset and a technique for generalized nuclear segmentation for computational pathology. IEEE transactions on medical imaging, 36(7):1550–1560, 2017.

[6] Dylan M Cable, Evan Murray, Luli S Zou, Aleksandrina Goeva, Evan Z Macosko, Fei Chen, and Rafael A Irizarry. Robust decomposition of cell type mixtures in spatial transcriptomics. Nature Biotechnology, 40(4):517–526, 2022.

[7] Pascal Vincent. A connection between score matching and denoising autoencoders. Neural computation, 23(7):1661–1674, 2011.

[8] Max Welling and Yee W Teh. Bayesian learning via stochastic gradient langevin dynamics. In Proceedings of the 28th international conference on machine learning (ICML-11), pages 681–688. Citeseer, 2011.

[9] Mehdi Mirza and Simon Osindero. Conditional generative adversarial nets. arXiv preprint arXiv:1411.1784, 2014.

[10] Kihyuk Sohn, Honglak Lee, and Xinchen Yan. Learning structured output representation using deep conditional generative models. Advances in neural information processing systems, 28, 2015.

[11] Andrew Brock, Jeff Donahue, and Karen Simonyan. Large scale gan training for high fidelity natural image synthesis. arXiv preprint arXiv:1809.11096, 2018.

[12] Takeru Miyato and Masanori Koyama. cgans with projection discriminator. arXiv preprint arXiv:1802.05637, 2018.

[13] Augustus Odena, Christopher Olah, and Jonathon Shlens. Conditional image synthesis with auxiliary classifier gans. In International conference on machine learning, pages 2642–2651. PMLR, 2017.

[14] Matthew Tancik, Pratul Srinivasan, Ben Mildenhall, Sara Fridovich-Keil, Nithin Raghavan, Utkarsh Singhal, Ravi Ramamoorthi, Jonathan Barron, and Ren Ng. Fourier features let networks learn high frequency functions in low dimensional domains. Advances in Neural Information Processing Systems, 33:7537–7547, 2020.

[15] Yang Song and Stefano Ermon. Generative modeling by estimating gradients of the data distribution. Advances in Neural Information Processing Systems, 32, 2019.

[16] Yang Song and Stefano Ermon. Improved techniques for training score-based generative models. Advances in neural information processing systems, 33:12438–12448, 2020.

[17] Olaf Ronneberger, Philipp Fischer, and Thomas Brox. U-net: Convolutional networks for biomedical image segmentation. In International Conference on Medical image computing and computer-assisted intervention, pages 234–241. Springer, 2015.

[18] Jonathan Ho, Ajay Jain, and Pieter Abbeel. Denoising diffusion probabilistic models. Advances in Neural Information Processing Systems, 33:6840–6851, 2020.

[19] Prafulla Dhariwal and Alexander Nichol. Diffusion models beat gans on image synthesis. Advances in Neural Information Processing Systems, 34:8780–8794, 2021.

[20] Nanxin Chen, Yu Zhang, Heiga Zen, Ron J Weiss, Mohammad Norouzi, and William Chan. Wavegrad: Estimating gradients for waveform generation. arXiv preprint arXiv:2009.00713, 2020.

[21] Diederik P Kingma and Jimmy Ba. Adam: A method for stochastic optimization. arXiv preprint arXiv:1412.6980, 2014.

[22] Bin Li, Wen Zhang, Chuang Guo, Hao Xu, Longfei Li, Minghao Fang, Yinlei Hu, Xinye Zhang, Xinfeng Yao, Meifang Tang, et al. Benchmarking spatial and single-cell transcriptomics integration methods for transcript distribution prediction and cell type deconvolution. Nature Methods, pages 1–9, 2022.

[23] Meng Zhang, Stephen W Eichhorn, Brian Zingg, Zizhen Yao, Kaelan Cotter, Hongkui Zeng, Hongwei Dong, and Xiaowei Zhuang. Spatially resolved cell atlas of the mouse primary motor cortex by merfish. Nature, 598(7879):137–143, 2021.

[24] William E Allen, Timothy R Blosser, Zuri A Sullivan, Catherine Dulac, and Xiaowei Zhuang. Molecular and spatial signatures of mouse brain aging at single-cell resolution. Cell, 186(1):194–208, 2023.

[25] Hu Zeng, Jiahao Huang, Haowen Zhou, William J Meilandt, Borislav Dejanovic, Yiming Zhou, Christopher J Bohlen, Seung-Hye Lee, Jingyi Ren, Albert Liu, et al. Integrative in situ mapping of single-cell transcriptional states and tissue histopathology in a mouse model of alzheimer's disease. Nature Neuroscience, 26(3):430–446, 2023.

[26] Ron Zeira, Max Land, Alexander Strzalkowski, and Benjamin J Raphael. Alignment and integration of spatial transcriptomics data. Nature Methods, 19(5):567–575, 2022.

[27] Maren Büttner, Zhichao Miao, F Alexander Wolf, Sarah A Teichmann, and Fabian J Theis. A test metric for assessing single-cell rna-seq batch correction. Nature methods, 16(1):43–49, 2019.

[28] Bosiljka Tasic, Zizhen Yao, Lucas T Graybuck, Kimberly A Smith, Thuc Nghi Nguyen, Darren Bertagnolli, Jeff Goldy, Emma Garren, Michael N Economo, Sarada Viswanathan, et al. Shared and distinct transcriptomic cell types across neocortical areas. Nature, 563(7729):72–78, 2018.

[29] Tommaso Biancalani, Gabriele Scalia, Lorenzo Buffoni, Raghav Avasthi, Ziqing Lu, Aman Sanger, Neriman Tokcan, Charles R Vanderburg, Åsa Segerstolpe, Meng Zhang, et al. Deep learning and alignment of spatially resolved single-cell transcriptomes with tangram. Nature methods, 18(11):1352–1362, 2021.

[30] Jiaqiang Zhu, Shiquan Sun, and Xiang Zhou. Spark-x: non-parametric modeling enables scalable and robust detection of spatial expression patterns for large spatial transcriptomic studies. Genome biology, 22(1):1–25, 2021.

[31] Congxue Hu, Tengyue Li, Yingqi Xu, Xinxin Zhang, Feng Li, Jing Bai, Jing Chen, Wenqi Jiang, Kaiyue Yang, Qi Ou, et al. Cellmarker 2.0: an updated database of manually curated cell markers in human/mouse and web tools based on scrna-seq data. Nucleic Acids Research, 51(D1):D870–D876, 2023.

[32] Ed S Lein, Michael J Hawrylycz, Nancy Ao, Mikael Ayres, Amy Bensinger, Amy Bernard, Andrew F Boe, Mark S Boguski, Kevin S Brockway, Emi J Byrnes, et al. Genome-wide atlas of gene expression in the adult mouse brain. Nature, 445(7124):168–176, 2007.
