# Supplementary material for: Integrating spatial and single-cell transcriptomics data using deep generative models with SpatialScope
Source: Nat Commun. 2023 Nov 29;14:7848. doi: 10.1038/s41467-023-43629-w (PMC10687049; doi:10.1038/s41467-023-43629-w)
Supplement: Supplementary file 4 — Reporting Summary [file 41467_2023_43629_MOESM4_ESM.pdf]

nature portfolio

Corresponding author(s): Can Yang

Last updated by author(s): Sep 1, 2023

Reporting Summary

Nature Portfolio wishes to improve the reproducibility of the work that we publish. This form provides structure for consistency and transparency in reporting. For further information on Nature Portfolio policies, see our Editorial Policies and the Editorial Policy Checklist.

Statistics

For all statistical analyses, confirm that the following items are present in the figure legend, table legend, main text, or Methods section.

n/a	Confirmed	
☐	☒	The exact sample size (*n*) for each experimental group/condition, given as a discrete number and unit of measurement
☐	☒	A statement on whether measurements were taken from distinct samples or whether the same sample was measured repeatedly
☒	☐	The statistical test(s) used AND whether they are one- or two-sided<br>*Only common tests should be described solely by name; describe more complex techniques in the Methods section.*
☐	☒	A description of all covariates tested
☐	☒	A description of any assumptions or corrections, such as tests of normality and adjustment for multiple comparisons
☐	☒	A full description of the statistical parameters including central tendency (e.g. means) or other basic estimates (e.g. regression coefficient) AND variation (e.g. standard deviation) or associated estimates of uncertainty (e.g. confidence intervals)
☐	☒	For null hypothesis testing, the test statistic (e.g. *F*, *t*, *r*) with confidence intervals, effect sizes, degrees of freedom and *P* value noted<br>*Give P values as exact values whenever suitable.*
☐	☒	For Bayesian analysis, information on the choice of priors and Markov chain Monte Carlo settings
☐	☒	For hierarchical and complex designs, identification of the appropriate level for tests and full reporting of outcomes
☐	☒	Estimates of effect sizes (e.g. Cohen's *d*, Pearson's *r*), indicating how they were calculated

Our web collection on statistics for biologists contains articles on many of the points above.

Software and code

Policy information about availability of computer code

Data collection

No software was used for data collection.

Data analysis

We used the newly developed Python package SpatialScope for data analysis. SpatialScope is in the Methods section and deposited at GitHub (https://github.com/YangLabHKUST/SpatialScope). The source code is released under the GNU General Public License version 3 (GPL>=3). All analysis codes for reproducing the results of the present study are publicly available at https://spatialscope-tutorial.readthedocs.io/

We used the following packages for downstream analysis:
Giotto [https://github.com/drieslab/Giotto] (v1.1.2)
C-SIDE [https://github.com/dmcable/spacexr] (v2.1.0)
SPARK-X [https://github.com/xzhoulab/SPARK] (v1.0.0)

In addition, we used the following software packages for comparative analysis:
Tangram [https://github.com/broadinstitute/Tangram] (v1.0.4)
CytoSPACE [https://github.com/digitalcytometry/cytospace] (v1.0.2)
SpatialDWLS [https://github.com/drieslab/Giotto] (v1.1.2)
RCTD [https://github.com/dmcable/spacexr] (v2.1.0)
Cell2location [https://github.com/BayraktarLab/cell2location] (v0.0.6)
CARD [https://github.com/YingMa0107/CARD] (v1.0.0)
SpaOTsc [https://github.com/zcang/SpaOTsc] (v1.0.0)
novoSpaRc [https://github.com/rajewsky-lab/novosparc] (v0.4.3)

DestVI [https://github.com/scverse/scvi-tools] (v0.8.0)
STRIDE [https://github.com/DongqingSun96/STRIDE](v0.0.1)
SPOTlight [https://github.com/MarcElosua/SPOTlight](v0.1.0)
DSTG [https://github.com/Su-informatics-lab/DSTG](v1.0.0)
gimVI [https://github.com/scverse/scvi-tools] (v0.8.0)
SpaGE [https://github.com/tabdelaal/SpaGE] (v1.0.0)
stPlus [https://github.com/xy-chen16/stPlus] (v0.0.5)
Seurat [https://satijalab.org/seurat] (v4.0.5)

For manuscripts utilizing custom algorithms or software that are central to the research but not yet described in published literature, software must be made available to editors and reviewers. We strongly encourage code deposition in a community repository (e.g. GitHub). See the Nature Portfolio guidelines for submitting code & software for further information.

Data

Policy information about availability of data

All manuscripts must include a data availability statement. This statement should provide the following information, where applicable:

- Accession codes, unique identifiers, or web links for publicly available datasets
- A description of any restrictions on data availability
- For clinical datasets or third party data, please ensure that the statement adheres to our policy

For the benchmarking datasets, the MERFISH MOp data were downloaded from the brain image library (https://doi.brainimagelibrary.org/doi/10.35077/g.8), the MERFISH Mouse brain data were downloaded from the project page (https://cellxgene.cziscience.com/collections/31937775-0602-4e52-a799-b6acdd2bac2e), the STARmap PLUS Hippocampus data were downloaded from the single cell portal project (https://singlecell.broadinstitute.org/single_cell/study/SCP1375).
For real data analysis, the 10x human heart and mouse brain cortex datasets were downloaded from the 10x official website (https://www.10xgenomics.com/resources/datasets), and the paired human heart and mouse brain cortex scRNA-seq reference are available from the project page (https://www.heartcellatlas.org/v1.html) and (https://celltypes.brain-map.org/rnaseq/mouse/v1-alm), respectively. Both Mouse cerebellum Slide-seq V2 dataset and the paired scRNA-seq reference were download from single cell portal project (https://singlecell.broadinstitute.org/single_cell/study/SCP948).

Human research participants

Policy information about studies involving human research participants and Sex and Gender in Research.

Reporting on sex and gender	N/A
Population characteristics	N/A
Recruitment	N/A
Ethics oversight	N/A

Note that full information on the approval of the study protocol must also be provided in the manuscript.

Field-specific reporting

Please select the one below that is the best fit for your research. If you are not sure, read the appropriate sections before making your selection.

[x] Life sciences   [ ] Behavioural & social sciences   [ ] Ecological, evolutionary & environmental sciences

For a reference copy of the document with all sections, see nature.com/documents/nr-reporting-summary-flat.pdf

Life sciences study design

All studies must disclose on these points even when the disclosure is negative.

Sample size	No sample size calculation was performed. SpatialScope was evaluated across four publicly available spatially resolved transcriptomics datasets in real data applications using as many samples as possible in these datasets, including human heart (spot sample size = 3,813) and mouse brain cortex data (spot sample size = 1,606) from 10x Visium datasets, mouse cerebellum data from Slide-seq dataset (spot sample size = 8,952), mouse MOp data from MERFISH dataset (spot sample size = 5,551).
Data exclusions	Following standard quality control practice, we retained genes with non-zero expression level on at least 10 spots and retained spots with non-zero expression for at least 50 genes for analysis, in order to avoid false positives.

Replication	We did not perform replication. Instead, we cross-validate the findings of the present study by comparing to other published molecular biology results.
Randomization	In our paper, we proposed a unified approach integrating scRNA-seq reference data and ST data using deep generative models. All data are public available and we do not perform any randomized controlled trial, so randomization is not relevant to this study.
Blinding	Blinding is not relevant to this study because we don't compare any case/control groups.

Reporting for specific materials, systems and methods

We require information from authors about some types of materials, experimental systems and methods used in many studies. Here, indicate whether each material, system or method listed is relevant to your study. If you are not sure if a list item applies to your research, read the appropriate section before selecting a response.

Materials & experimental systems

n/a	Involved in the study
☒	☐ Antibodies
☒	☐ Eukaryotic cell lines
☒	☐ Palaeontology and archaeology
☒	☐ Animals and other organisms
☒	☐ Clinical data
☒	☐ Dual use research of concern

Methods

n/a	Involved in the study
☒	☐ ChIP-seq
☒	☐ Flow cytometry
☒	☐ MRI-based neuroimaging
